# LXRα agonists ameliorates acute rejection after liver transplantation via ABCA1/MAPK and PI3K/AKT/mTOR signaling axis in macrophages

**DOI:** 10.1186/s10020-025-01153-1

**Published:** 2025-03-14

**Authors:** Xiaoyan Qin, Dingheng Hu, Qi Li, Shiyi Zhang, Zheng Qin, Liangxu Wang, Rui Liao, Zhongjun Wu, Yanyao Liu

**Affiliations:** 1 Department of Hepatobiliary Surgery, The First Affiliated Hospital of Chongqing Medical University, No.1 Youyi Road, Yuzhong District, Chongqing, China; 2 Department of General Surgery and Trauma Surgery, Children’s Hospital of Chongqing Medical University, Ministry of Education Key Laboratory of Child Development and Disorders, National Clinical Research Center for Child Health and Disorders, China International Science and Technology Cooperation Base of Child Development and Critical Disorders, Chongqing Key Laboratory of Pediatrics, Chongqing, China

**Keywords:** Liver transplantation, Acute rejection, LXRα, Macrophage, M1 polarization, Inflammation

## Abstract

**Introduction:**

Liver X receptor α (LXRα) plays an important role in inflammatory immune response induced by hepatic ischemia-reperfusion injury (IRI) and acute rejection (AR). Macrophage M1-polarization play an important role in the occurrence and development of AR. Although the activation of LXR has anti-inflammatory effects, the role of LXRα in AR after liver transplantation (LT) has not been elucidated.

**Objective:**

We aimed to investigate LXRα anti-inflammatory and macrophage polarization regulation effects and mechanisms in acute rejection rat models.

**Methods:**

LXRα anti-inflammatory and liver function protective effects was initially measured in primary Kupffer cells and LT rat models. Subsequently, a flow cytometry assay was used to detect the regulation effect of LXRα in macrophage polarization. HE staining, TUNEL and ELISA were used to evaluate the co-treatment effects of TO901317 and tacrolimus on hepatic apoptosis and liver acute rejection after LT.

**Results:**

In this study, we found that LPS can inhibit the expression of LXRα and activate MAPK pathway and PI3K/AKT/mTOR. We also found that LXRα agonist (TO901317) could improve liver function and rat survival after LT by activating the level of ABCA1 and inhibiting MAPK. TO901317 could inhibit macrophage M1-polarization by activating PI3K/AKT/mTOR signal pathway to improve the liver lesion of AR rats after liver transplantation. Additionally, co-treatment with TO901317 and tacrolimus more effectively alleviated the damaging effects of AR following LT than either drug alone.

**Conclusion:**

Our results suggest that the activation of LXRα can improve liver function and rat survival after LT by regulate ABCA1/MAPK and PI3K/AKT/mTOR signaling axis in macrophages.

**Supplementary Information:**

The online version contains supplementary material available at 10.1186/s10020-025-01153-1.

## Introduction

Liver transplantation (LT) represents the only proven efficacious treatment for end-stage liver disease (ESLD) (Im et al. 2019; Mittler et al. 2022). However, acute rejection (AR) occurs in about 30% of LT patients within 12 months after operation. AR is also associated with the poor prognosis of LT patients (Levitsky et al. 2017; Montano-Loza et al. 2023). Although immunosuppressants can prevent and alleviate AR after LT, immunosuppressive agents are associated with many side effects and contraindications, such as metabolic disorders and tumor recurrence (Fan et al. 2023). Accordingly, exploring the AR pathogenesis and new therapeutic targets for improved LT prognosis is crucial.

Macrophages are key cells of the innate immune system and are essential in governing the immune microenvironment in the donor liver after LT (Ye et al. 2020). Macrophages are categorized into two types: classical M1 and alternative M2, which are distinguished based on their respective functions. M1 macrophages induce early immune responses by antigen presentation and secreting pro-inflammatory factors, including IL-1β/6 and tumor necrosis factor-alpha (TNF-α) (Yunna et al. 2020). M2 macrophages hinder the adaptive immune responses through the secretion of high concentrations of anti-inflammatory factors, including transforming growth factor β (TGFβ) and IL-10 (Witherel et al. 2021). Inhibition of M1 polarization and promotion of M2 polarization can maintain the immune homeostasis of the donor liver and induce immune tolerance following LT (Montano-Loza et al. 2023; Ye et al. 2020). Consequently, the regulation of macrophage polarization is crucial for preventing and treating AR after LT.

Liver x receptors (LXR), including LXRα and LXRβ (belonging to the nuclear receptor superfamily), play key roles in maintaining liver cholesterol homeostasis and lipid metabolism (Personnaz et al. 2022). LXRα is overexpressed in the liver, adipose tissue, and macrophages (Zhao et al. 2021). Recent studies have found that LXRα activation can enhance the prognosis of patients with inflammatory diseases, including atherosclerosis, Alzheimer's disease, and nonalcoholic fatty liver, by inducing anti-inflammatory effects (Che et al. 2021; Dib et al. 2023; Huang et al. 2018). Besides, LXRα activation can protect against myocardial and acute hepatic inflammation by inhibiting oxidative stresses and reducing mitochondrial-mediated apoptosis (He et al. 2014; Endo-Umeda and Makishima 2019). Furthermore, LXRα in macrophages can regulate lipid metabolism and the immune response (Yang et al. 2016). A previous study showed that the LXRα pathway modulates macrophage survival through inflammasome activation (Tabraue et al. 2019). Another study found that LXRα agonists can alleviate skin dermatitis and atherosclerosis. Notably, LXRα can negatively regulate inflammatory gene expression in macrophages (Joseph et al. 2003). However, only a few studies have assessed the role of LXRα in organ transplantation. Besides, the involvement and specific mechanism behind LXRα agonists in AR remains unclear. This study aimed to explore the specific mechanism by which LXRα agonists regulate the ABCA1/MAPK and PI3K/AKT/mTOR signaling axes of macrophages, lower pro-inflammatory factor release, impede macrophage M1 polarization, and relieve AR.

## Materials and methods

### Ethical statement

The experiments followed the relevant institutional licensing committee (Chongqing Medical University Ethics Committee) guidelines and regulations. Furthermore, this study followed the ARRIVE guidelines and received approval from the Chongqing Medical University Ethics Committee (NO. 2023-393).

**Table 1:** Sequences

Name	Forward primer	Reverse primer
LXRα	GAGTGTCGACTTCGCAATGG	CCTTCTTCTTGCCGTTCAGT
LXRβ	CAGGCTTGCAGGTGGAATTC	ATGGCGATAAGCAAGGCATACT
ABCA1	GGCAATGAGTGTGCCAGAGTTA	TAGTCACATGTGGCACCGTTTT
ABCG1	TCCCACCTGTAAGTAATTGCA	TCGGACCCTTATCATTCTCTACAGA
TNF-α	CTACGTGCTCCTCACCCACACCGT	ACCTCAGCGCTGAGCAGGTCCCCC
GADPH	GGTGGACCTCATGGCCTACA	CTCTCTTGCTCTCAGTATCCTTGCT

**Table 2:** Antibodies

Primary antibody	Dilution	Supplier	Code	Primary antibody	Dilution	Supplier	Code
LXRα	1:1000	abcam	ab176323	p-4E-BP	1:1000	CST	2855
ABCA1	1:1000	abcam	ab66217	4E-BP	1:1000	CST	9644
ABCG1	1:1000	abcam	ab52617	IL-6	1:1000	Proteintech	218651-1-AP
p-AKT	1:1000	abcam	ab38449	TNF-α	1:1000	Proteintech	17590-1-AP
AKT	1:500	abcam	ab8805	caspase-6	1:1000	CST	9762
p-mTOR	1:1000	abcam	ab181268	cleaved caspase-6	1:1000	CST	9761
mTOR	1:1000	abcam	ab134903	caspase-3	1:1000	CST	14220
LXRβ	1:1000	CST	13519	cleaved caspase-3	1:1000	CST	ab214430
Erk1/2	1:1000	CST	4695	caspase-9	1:1000	CST	9508
p-Erk1/2	1:1000	CST	4370	cleaved caspase-9	1:1000	abcam	ab52298
P38	1:1000	CST	8690	Bcl-2	1:1000	Proteintech	26593-1-AP
p-P38	1:1000	CST	4511	Bax	1:1000	Proteintech	50599-2-lg
JNK	1:1000	CST	9252	β-actin	1:1000	CST	4970
p-JNK	1:1000	CST	4668	iNOS	1:1000	Proteintech	18985-1-AP
p-PI3K	1:1000	CST	17366	Arg-1	1:1000	Proteintech	16001-1-AP
PI3K	1:1000	CST	4249	F4/80-FITC	1:100	Bosterbio	A0-8751
p-S6K	1:1000	CST	9204	CD86-PE	1:100	Biolegend	200307
S6K	1:1000	CST	9202

**Table 3:** Experimental groups

Groups	Intervention	Intervention time
sham group	Abdominal incision to expose the hepatic portal vein	7 days
AR group	Liver transplantation	7 days
TO90 (1 mg) group	1 mg/kg/day	7 days
TO90 (10 mg) group	10 mg/kg/day	7 days
TO90 (50 mg) group	50 mg/kg/day	7 days
TO90+ABCA1shRNA group	TO90 (50 mg/kg/day)+ABCA1shRNA	7 days
TO90+LY294002	TO90 (50 mg/kg/day)+LY294002(2 mg/kg/day)	7 days
TAC group	Tacrolimus (1 mg/kg/day)	7 days
TO90+TAC group	TO90 (50 mg/kg/day)+Tacrolimus (1 mg/kg/day)	7 days

### LT model construction and treatment

Brown Norway (BN) and Lewis (LEW) rats (both were SPF Males, 200–220 g) sourced from the Chongqing Medical University experimental animal center (Chongqing, China) were housed in a controlled environment free from pathogens. Orthotopic LT was performed employing a novel magnetic anastomosis technique, as previously described (Yang et al. 2018). The rats were randomly divided into nine groups: (1) rats in the sham group underwent surgery via an abdominal incision to expose the hepatic portal vein; (2) the rats in the acute rejection (AR) group (LEW and BN rats were donors and recipients, respectively) did not receive any treatment after liver transplantation; (3) in the TO90 (1 mg) group, the rats were given TO901317 (TO90) (1 mg/kg/day) after LT for 7 days. (4) in the TO90 (10 mg) group, the rats were given TO90 (10 mg/kg/day) after LT for 7 days. (5) in the TO90 (50 mg) group, the rats were given TO90 (50 mg/kg/day) after LT for 7 days. (6) in the TO90+ABCA1shRNA group, the rats were given TO90 (50 mg/kg/day) for 7 days and transfected with ABCA1 specific shRNA after LT. (7) in the TO90+LY294002 group, the rats were given TO90 (50 mg/kg/day) and LY294002 (2 mg/kg/day) after LT for 7 days. (8) in the TAC group, the rats were given tacrolimus (1 mg/kg/day) after LT for 7 days. (9) in the TO90+TAC group, the rats were given TO90 (50 mg/kg/day) and tacrolimus (1 mg/kg/day) after LT for 7 days (Table 3 summarizes all the experimental groups). Survival experiments were performed with 6 rats per experimental group (except TO90+ABCA1shRNA group). All drug interventions were administered to recipient rats, with the first dose given immediately after surgery and subsequent doses administered every 24 h. TO90 and LY294002 were administered via intraperitoneal injection, ABCA1shRNA via tail vein injection, and tacrolimus was administered orally. The rats were euthanized at various time intervals, then serum samples and liver tissues were obtained and kept at −80 °C.

### Isolation of liver macrophage

Initially, the rats were administered D-Hanks’ Balanced Salt Solution buffer (Solarbio) through the portal vein. A solution of Type IV collagenase (0.5 mg/mL, Solarbio) was administered via the portal vein once the liver color changed from red to yellow. The digestion process was allowed to proceed for a duration of 5 min. The aforementioned treatment was iterated 5 to 6 times till the liver surface exhibited a granular texture. The liver was thereafter removed, broken into smaller pieces, and immersed in D-Hanks' solution containing 0.1 mg/mL of type IV collagenase for a duration of 30 min. Macrophages were ultimately separated by employing the Percoll solution (Solarbio).

### In vitro transfection and lipopolysaccharide stimulation

The cells were stimulated with 1, 10, 100, and 200 ng/mL lipopolysaccharides (LPS) (Beyotime, Beijing, China) following the experimental requirements. ABCA1-specific shRNA and NC in lentivirus vectors (Genepharma, Shanghai, China) were prepared and incubated following the manufacturer's instructions for in vitro transfection. Determining the potency of ABCA1 shRNA was conducted through Western blot (WB). Then, rats were transfected with ABCA1 shRNA through tail-vein injection 14 days before LT. The transfection efficacy of animals was also determined by RT-qPCR analysis. The shRNA sequence targeting rat ABCA1 mRNA was GACCACCCTAGAAGAAATATT.

### qRT-PCR

RNA was extracted from rat liver using trizol while synthesizing cDNA from RNA through PrinmeScrip RT buff (both from Takara, Tokyo, Japan). QPCR primers were synthesized, as shown in Table 1 (Shanghai Sangon Biotech, China). qRT-PCR test was performed following the instructions of the reagent manufacturer. Every sample was evaluated three times. The data analysis was performed employing the 2−ΔΔCt method.

### Analysis of serum cytokines and liver enzymes

Serum IL-1β, IL-6 and TNF-α levels were determined with an enzyme-linked immunosorbent assay (ELISA) kit (Neobioscience, Beijing, China) according to the manufacturer’s guidelines to measure the release of inflammatory cytokines. A liver enzyme kit (Jiancheng Bioengineering Institute in Nanjing, China) was deployed to assess serum alanine aminotransferase (ALT) and aspartate aminotransferase (AST) levels per the protocols.

### Western blotting

This study utilized a combination of RIPA buffer and a protease inhibitor for total protein extraction while using a 10% SDS-PAGE gel for protein sample separation. The separated samples were transferred onto a PVDF membrane, which was then sectioned based on the molecular weight of the desired protein and incubated with primary antibody solution at 4 ℃ for 12 h (Table 2) as well as with a secondary antibody at 4 ℃ for 2 h. Finally, the grayscale value was determined through a chemiluminescence reaction using a gel imaging system. The immunoreactive bands were quantified using ImageJ.

### HE staining and TdT-mediated dUTP-biotin nick and labeling

First, the liver tissue was removed and fixed in 4% paraformaldehyde for 24 h. Then, it was sliced into 5 μM sections, which were placed in xylene for 20 min twice, followed by immersion in ethanol for 5 min twice and in 75% ethanol for 5 min. Subsequently, the sections were washed with distilled water more than three times and stained with hematoxylin–eosin (HE). AR was graded based on Banff criteria. The hepatocyte apoptosis level was determined by the use of a terminal deoxynucleotidyl transferase dUTP incision end labeling (TUNEL) kit (Beyotime, Shanghai, China). Our study employed a fluorescence microscope for imaging under different magnifications.

### Flow cytometry

Macrophages from rat liver tissues were suspended individually in PBS, followed by being subjected to staining with an antibody in darkness at 4 ℃ for 1 h and twice washing with PBS after staining. The Kupffer cells (KCs) were stained using the F4/80-FITC antibody to determine purity. Furthermore, the cells went through staining with both F4/80-FITC and CD86PE antibodies for the ratio experiment involving M1-type cells, were assessed with the CytoFLEX instrument, and were analyzed using the CytExpert 2.4.0 software. Table 2 lists the specific antibodies and their respective dilution ratios.

#### Statistical analyses

GraphPadPrism8.0 and SPSS2.0 software were used for t-test, one-way ANOVA, Mann–Whitney test, and chi-square test. Each experiment was repeated thrice. *P* < 0.05 indicated a statistically significant difference.

## Results

### LPS increases the expression of MAPK and AKT/mTOR pathway-associated proteins and downregulates LXRα and ABCA1

**Fig. 1:**
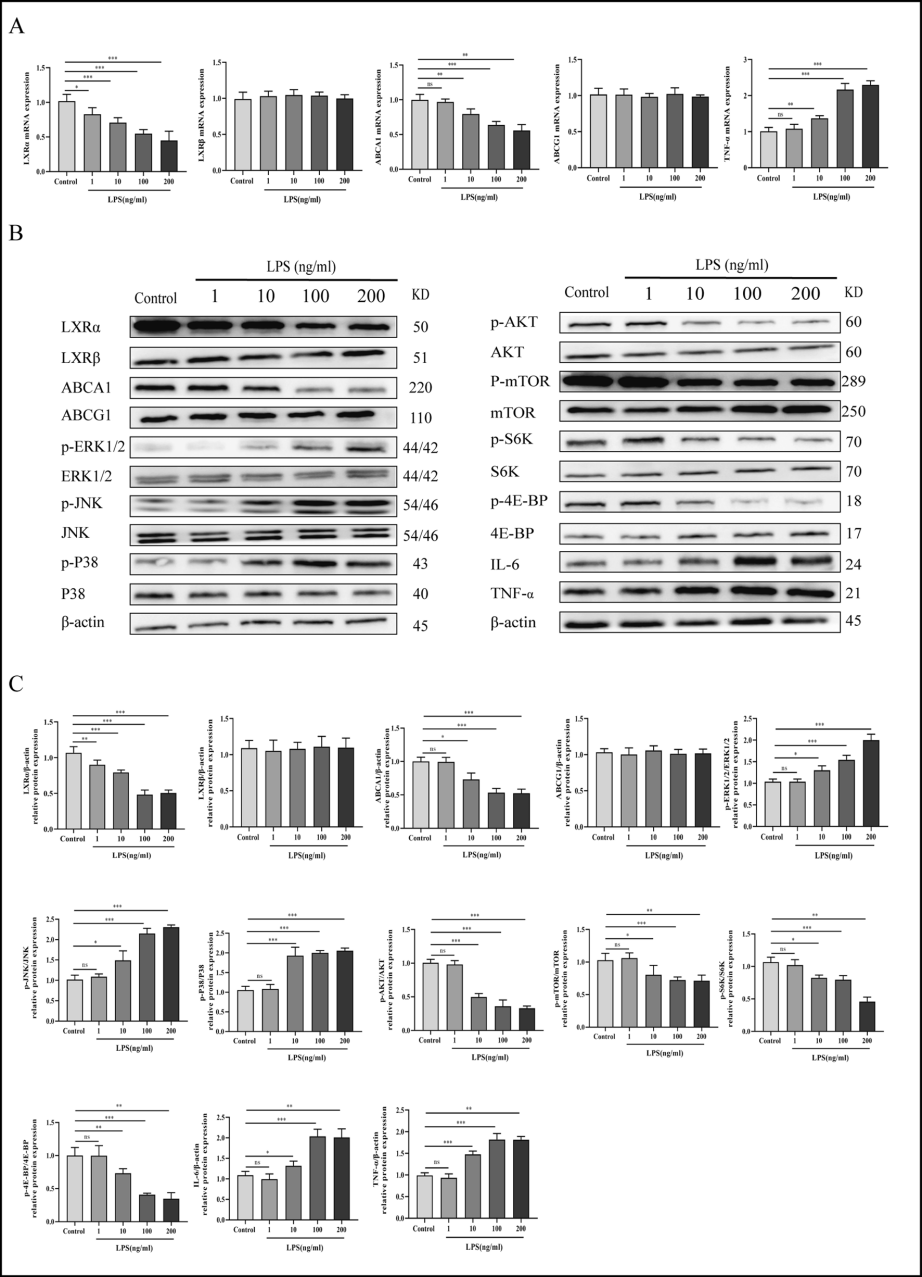
LPS inhibits the expression of LXRα and ABCA1 in vitro. **A** mRNA expression of LXRα, LXRβ, ABCA1, ABCG1 and TNF-α in kupffer cell stimulated by different concentrations of LPS (1, 10, 100, 200 ng/mL) for 24 h. **B**, **C** Protein expression of LXRα/ABCA1/MAPK and AKT/mTOR signaling pathways. One-way ANOVA was used to determine the statistical differences between groups. n = 6, ^*ns*^*P* > 0.05, *P < 0.05, **P < 0.01, ***P < 0.001

LPS-challenged macrophages, as a classic in vitro model of liver AR, were constructed to examine the role of LXR and its downstream target genes. A macrophage inflammation model was developed using LPS to determine the expression of LXR subtypes. RT-PCR results demonstrated that LPS stimulation decreased LXRα and ABCA1 mRNA levels in a concentration-dependent manner compared with the control group. However, LPS stimulation did not alter the mRNA levels of LXRβ and ABCG1. Furthermore, LPS stimulation increased the mRNA levels of TNF-α (Fig. 1A). Also, LPS stimulation decreased LXRα, ABCA1, p-AKT, p-mTOR, p-S6K, and p-4E-BP protein levels while increasing those of p-ERK1/2, JNK, p38, IL-6, and TNF-α. These changes were dependent on LPS concentration. However, LPS concentration did not alter the expression of LXRβ and ABCG1 (Fig. 1B, C). These findings indicate that LXRα may possess a possible anti-inflammatory function, as opposed to LXRβ.

### ABCA1 inhibition reverses the anti-inflammation effect of TO901317 in vitro

**Fig. 2:**
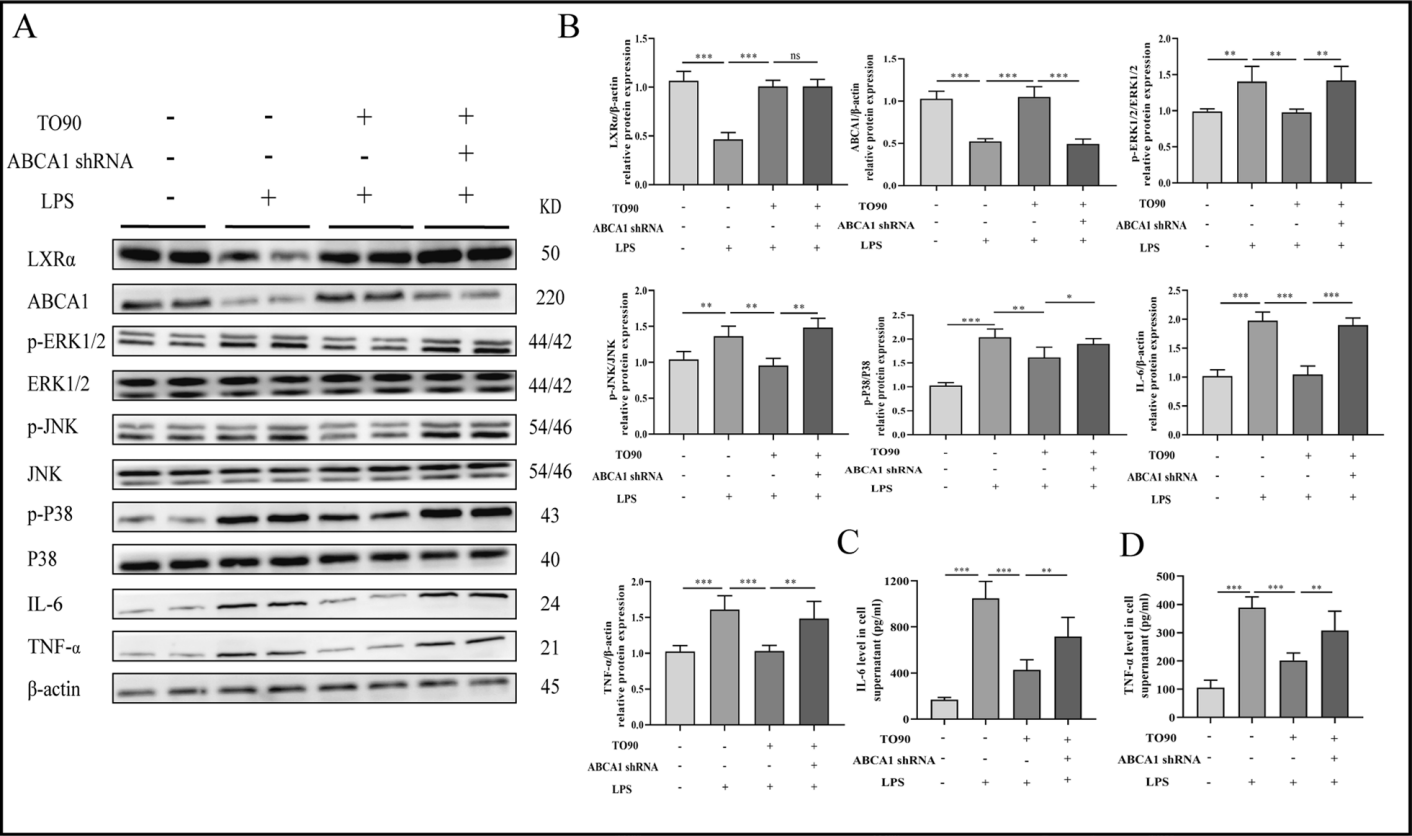
Relation of LXRα and ABCA1. **A**, **B** Protein expression levels of LXRα/ABCA1/MAPK signaling pathway in different groups (Kupffer cell stimulated by 200 ng/mL concentrations of LPS for 24 h or/and 10 μm concentrations of TO90 for 24 h). **C**, **D** Levels of IL-6 and TNF-α in cell supernatant. One-way ANOVA was used to determine the statistical differences between groups. n = 6, ^*ns*^*P* > 0.05, *P < 0.05, **P < 0.01, ***P < 0.001

TO901317 (TO90), as an LXRα agonist, can ameliorate inflammation by activating LXRα. Herein, the expression of ABCA1 in macrophages was knocked down using ABCA1 shRNA to identify anti-inflammatory mechanisms of LXRα. The WB showed that TO90 reduced the LPS-induced phosphorylation of JNK, P38, and ERK1/2 alongside IL-6 and TNF-α expression by activating LXRα/ABCA1 expression. However, ABCA1 knockdown reversed the effect of TO90 (Fig. 2A, B). Meanwhile, ELISA was deployed to detect the expression of the inflammatory factor in the culture medium. TO90 decreased LPS-induced IL-6 and TNF-α expression. Similarly, ABCA1 knockdown reversed this effect (Fig. 2C, D).

### TO901317 alleviates AR after LT

**Fig. 3:**
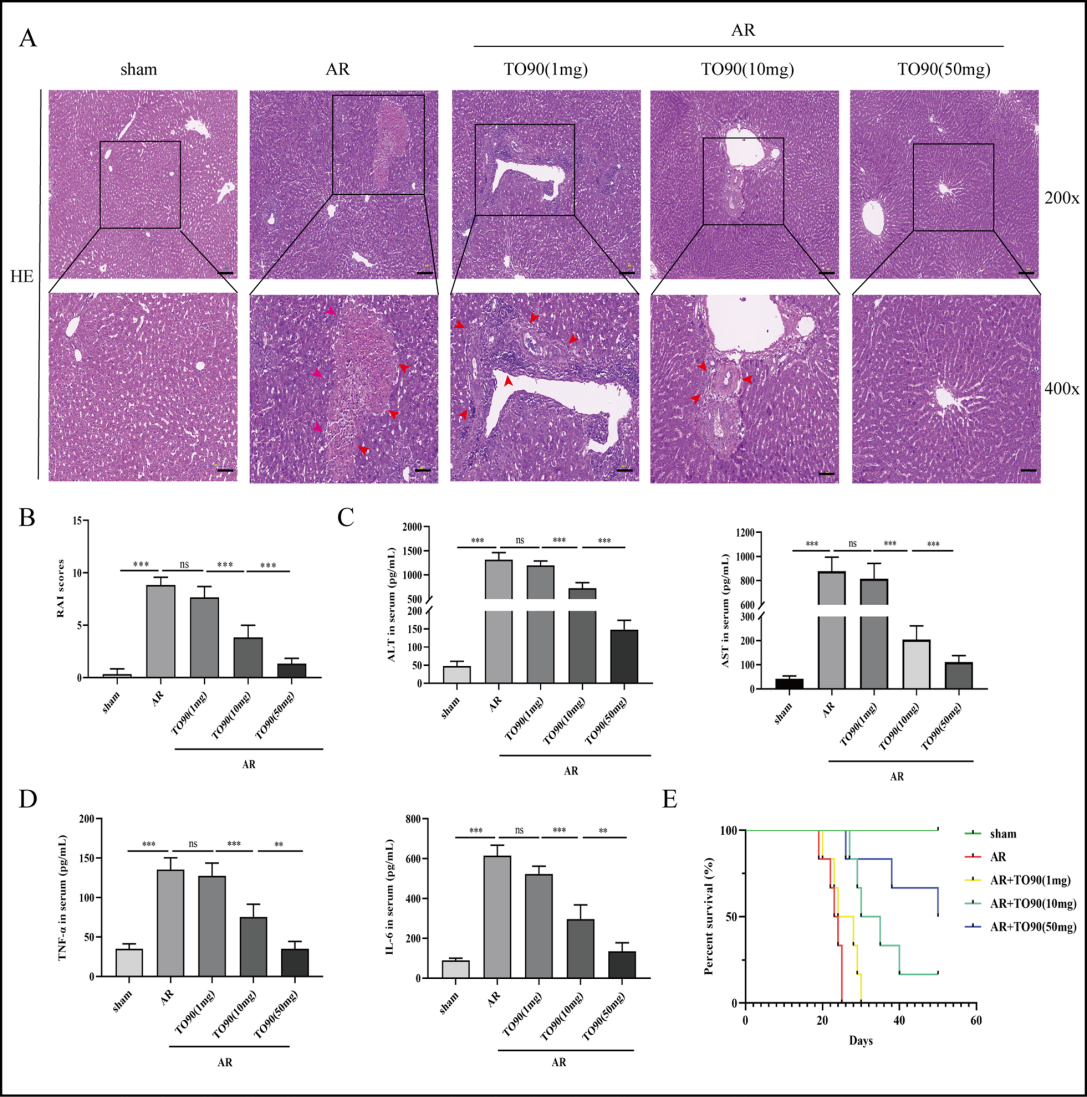
TO901317 alleviate AR in vivo. **A** Pathological changes in the liver following treatment with TO901317 (1, 10, 50 mg/kg/day) during AR (magnification, ×200/×400; scale bar = 100 μm) (The area indicated by the red arrow shows inflammatory cell infiltration in the portal area and hepatic sinusoids, cholangitis and venulitis, as well as hepatic parenchymal cell damage). **B** RAI scores was classified based on Banff patterns. **C** Serum levels of ALT and AST. **D** Serum levels of TNF-α and IL-6. **E** Survival rate of rats (each group n = 6). Statistical differences among groups were assessed by one-way ANOVA. n = 6, ^*ns*^*P* > 0.05, *P < 0.05, **P < 0.01, ***P < 0.001

The protective effect of LXRα on AR after LT was assessed using an AR rat model. HE staining showed that liver AR was characterized by bile duct injury, portal vein inflammatory cell infiltration, and endothelial inflammation. However, TO90 (1, 10, 50 mg) significantly decreased these effects in a dose-dependent manner (Fig. 3A). The RAI score also decreased with increasing TO90 concentrations (Fig. 3B). Similarly, TO90 treatment significantly decreased ALT, AST, TNF-α, and IL-6 serum levels. Furthermore, post-transplantation TO90 treatment exerted a significant improvement influence on liver functions (Fig. 3D). Also, the AR+TO90 treatment group exhibited better survival outcomes than other groups after LT (Fig. 3E).

### TO901317 inhibits hepatocellular apoptosis in AR after LT

**Fig. 4:**
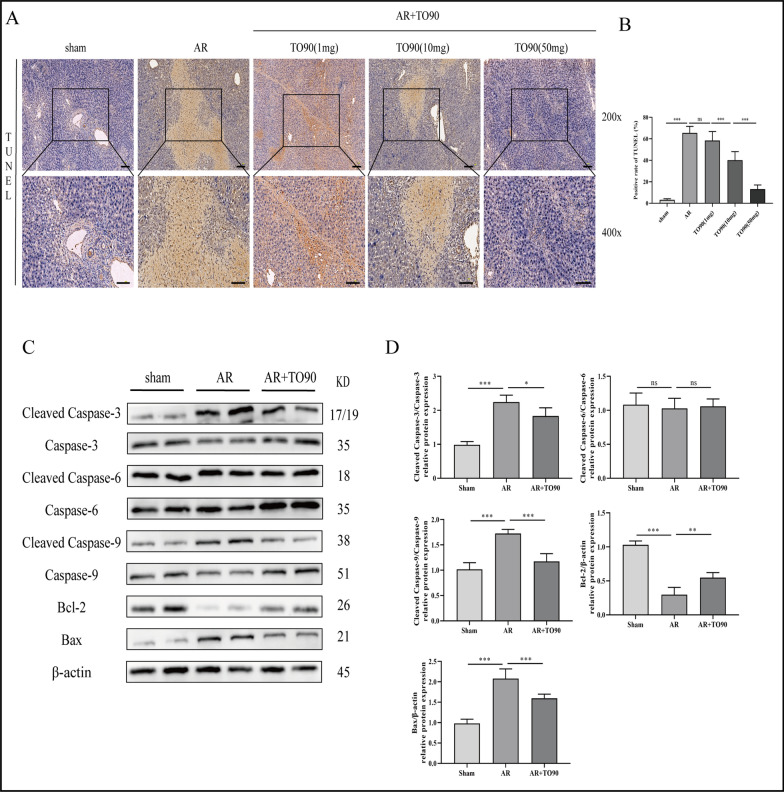
TO901317 inhibited the apoptosis of hepatocytes during AR. **A**, **B** Hepatic apoptosis following treatment with TO901317 (1, 10, 50 mg/kg/day) during AR (magnification, ×400; scale bar = 100 μm). **C**, **D** Protein expression levels of apoptosis related proteins. One-way ANOVA was used to determine the statistical differences between groups. n = 6, ^*ns*^*P* > 0.05, *P < 0.05, **P < 0.01, ***P < 0.001

The role of LXRα in AR after LT was further assessed by detecting hepatocyte apoptosis in rats after LT. TO90 significantly lowered hepatocyte apoptosis rate. Notably, the degree of therapeutic effect had a positive correlation with the dosage (Fig. 4A, B). Similarly, WB showed that TO90 reduced Cleaved Caspase-3/9 and Bax protein levels and elevated that of Bcl-2 (Fig. 4C, D). These results suggest that LXRα activation can effectively inhibit hepatocyte apoptosis in AR rats.

### ABCA1 inhibition reverses the alleviating effect of TO901317 on AR after LT

**Fig. 5:**
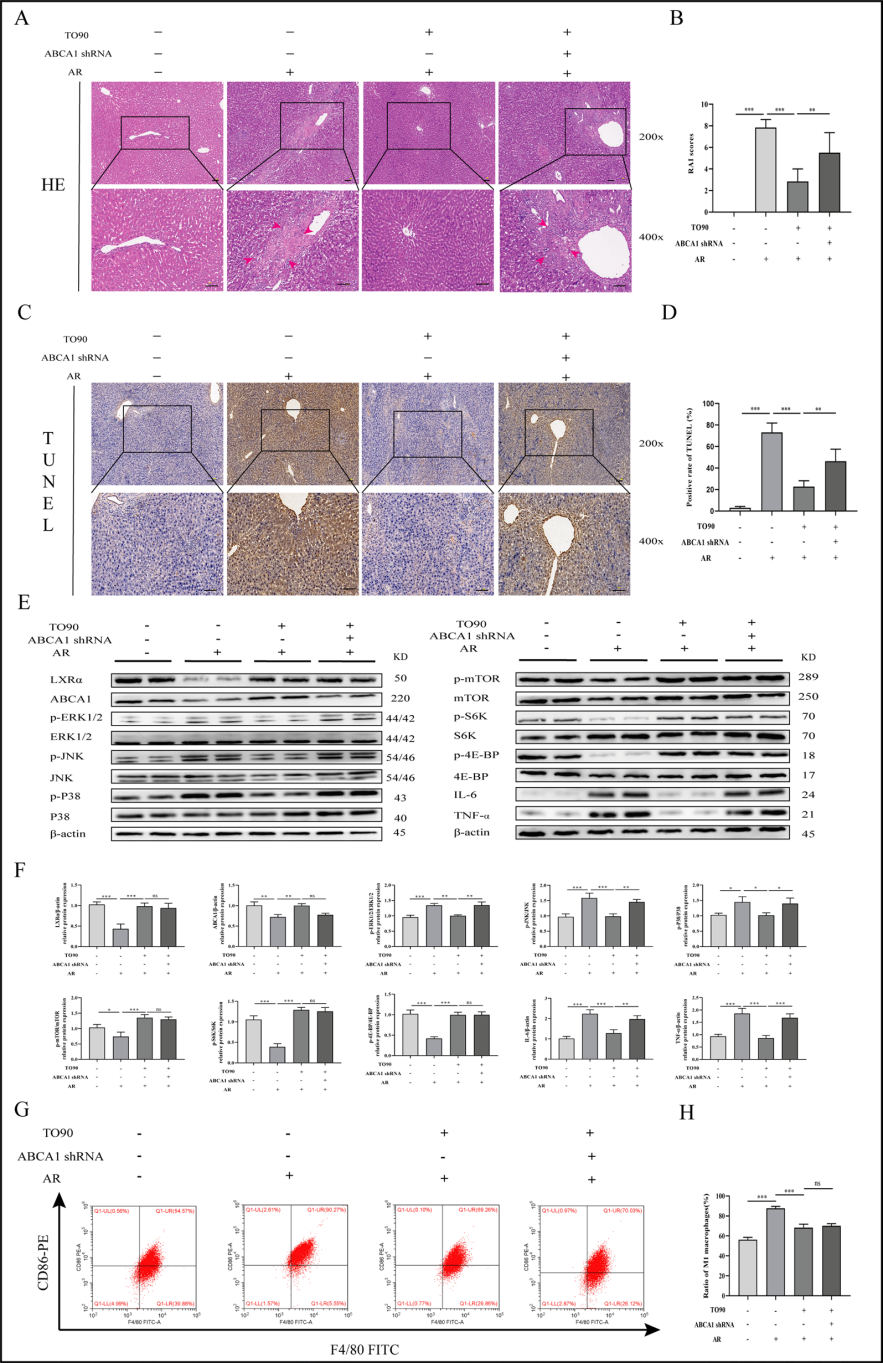
Effects of ABCA1 in vivo. **A**, **B** Pathological changes in the liver following treatment with TO901317 and ABCA1 shRNA during AR (magnification, ×200/400; scale bar = 100 μm) (The area indicated by the *red arrow* shows inflammatory cell infiltration in the portal area and hepatic sinusoids, cholangitis and venulitis, as well as hepatic parenchymal cell damage). **C**, **D** Hepatic apoptosis following treatment with TO901317 and ABCA1 shRNA during AR (magnification, ×200; scale bar = 100 mm). **E**, **F** Protein expression levels of LXRα/ABCA1/MAPK and mTOR signaling pathway factors. **E** Detection of the proportion of M1 polarization by flow cytometry, macrophages were separated with FITC-labeled F4/80, and M1-type polarization was separated with PE-labeled CD86. One-way ANOVA was used to determine the statistical differences between groups. n = 6, ^*ns*^*P* > 0.05, *P < 0.05, **P < 0.01, ***P < 0.001

ABCA1 was knocked down using ABCA1 shRNA to confirm whether LXRα regulates ABCA1's protective effect on liver injury in the rat AR model. HE staining showed that TO90 significantly alleviated pathological changes, such as bile duct injury, portal vein inflammatory cell infiltration, and endothelial inflammation in AR rats. However, ABCA1 knockdown reversed these changes (Fig. 5A, B). TO90 also alleviated hepatic apoptosis, while ABCA1 inhibition partly reversed that effect (Fig. 5C, D). The MAPK and PI3K/AKT/mTOR signal pathway expression was assessed using WB to further explore the mechanism by which LXRα alleviates liver lesions in AR rats. TO90 impeded ERK1/2, JNK, and P38 phosphorylation and IL-6 and TNF-α expression in the liver of AR rats. However, ABCA1 knockdown attenuated the inhibitory effect. In addition, TO90 increased mTOR, S6K, and 4E-BP phosphorylation in the livers of AR rats. Interestingly, ABCA1 knockdown did not reverse this effect (Fig. 5E, F). Flow cytometry revealed that TO90 caused a significant reduction in the M1-type ratio in macrophages. Similarly, ABCA1 knockdown did not affect this phenomenon (Fig. 5G, H). These results indicate that the LXRα/ABCA1 axis attenuates liver inflammation in AR rats by inhibiting the MAPK signal pathway rather than regulating macrophage M1-polarization.

### TO901317 alleviates AR after LT by inhibiting macrophage M1-polarization via PI3K/AKT/mTOR pathway

**Fig. 6:**
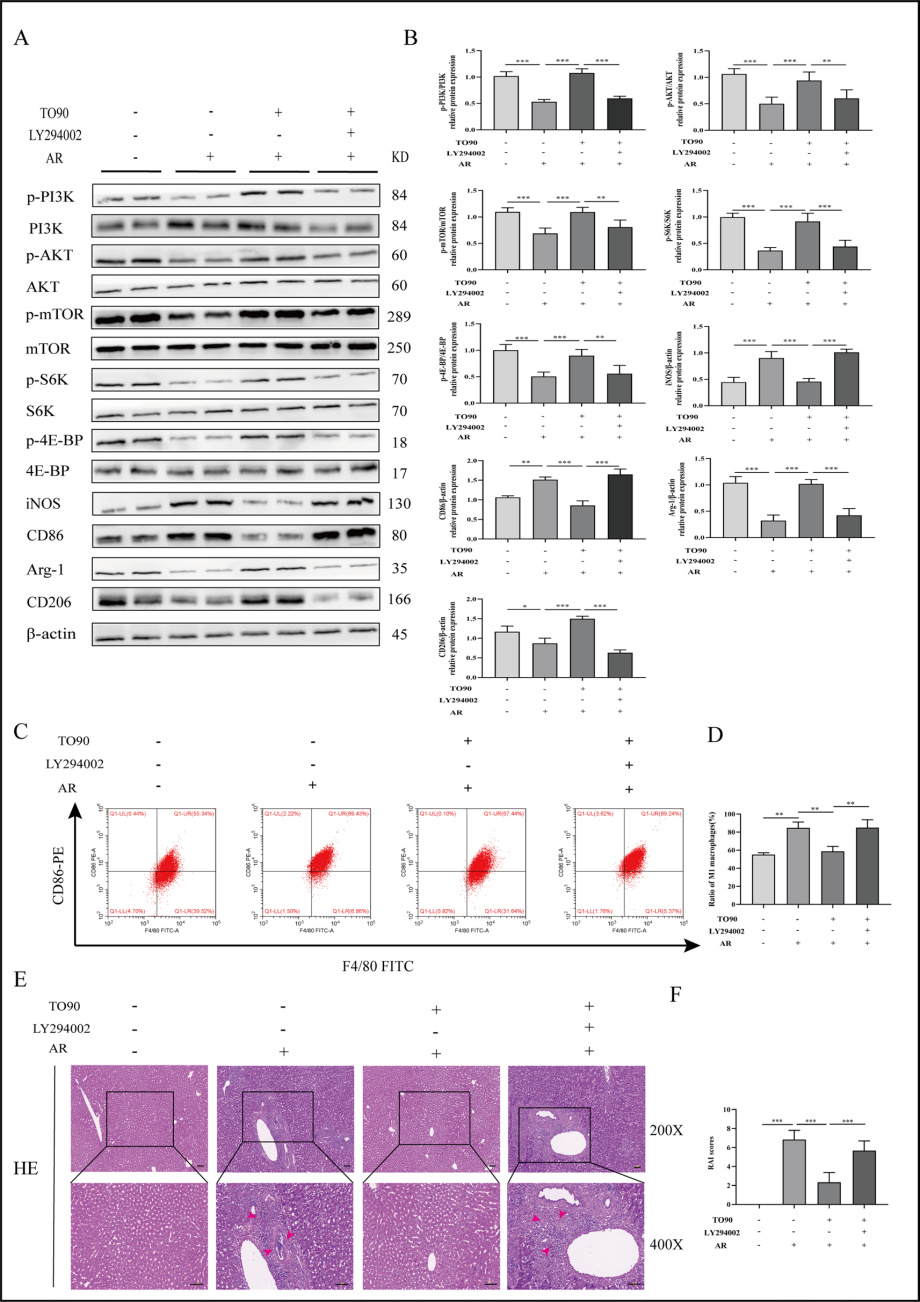
LXRα improves AR after liver transplantation by activating PI3K/AKT/mTOR pathway to regulate macrophage polarization. **A**, **B** Protein expression levels of PI3K/AKT/mTOR signaling pathway factors. **C**, **D** Detection of the proportion of M1 polarization by flow cytometry, macrophages were separated with FITC-labeled F4/80, and M1-type polarization was separated with PE-labeled CD86. **E**, **F** Pathological changes in the liver following treatment with TO901317 or LY294002 during AR (magnification, ×200; scale bar = 100 μm) (The area indicated by the red arrow shows inflammatory cell infiltration in the portal area and hepatic sinusoids, cholangitis and venulitis, as well as hepatic parenchymal cell damage). One-way ANOVA was used to determine the statistical differences between groups. n = 6, *P < 0.05, **P < 0.01, ***P < 0.001

Herein, we further investigated the role of LXRα in attenuating M1-polarization via the PI3K/AKT/mTOR pathway via LY294002 (a broad-spectrum inhibitor of PI3K to inhibit PI3K/AKT/mTOR signaling pathway) treatment, followed by employing WB to assess the protein expression of this pathway in the liver. The results indicated that TO90 increased p-PI3K and p-AKT, p-mTOR, and p-4E-BP protein levels and down-regulated iNOS, CD86, Arg-1, and CD206 (Fig. 6A, B). Similarly, LY294002 reversed TO90 inhibitory effect on macrophage M1-polarization (Fig. 6C, D). Furthermore, HE staining illustrated that LY294002 reversed TO90's protective effect on the liver of AR rats in vivo (Fig. 6E, F). These indicate that TO90 can impede macrophage M1-polarization through the activation of the PI3K/AKT/mTOR pathway, thus alleviating AR after LT.

### The effect of TO901317 combined with tacrolimus on the outcome of AR after LT

**Fig. 7:**
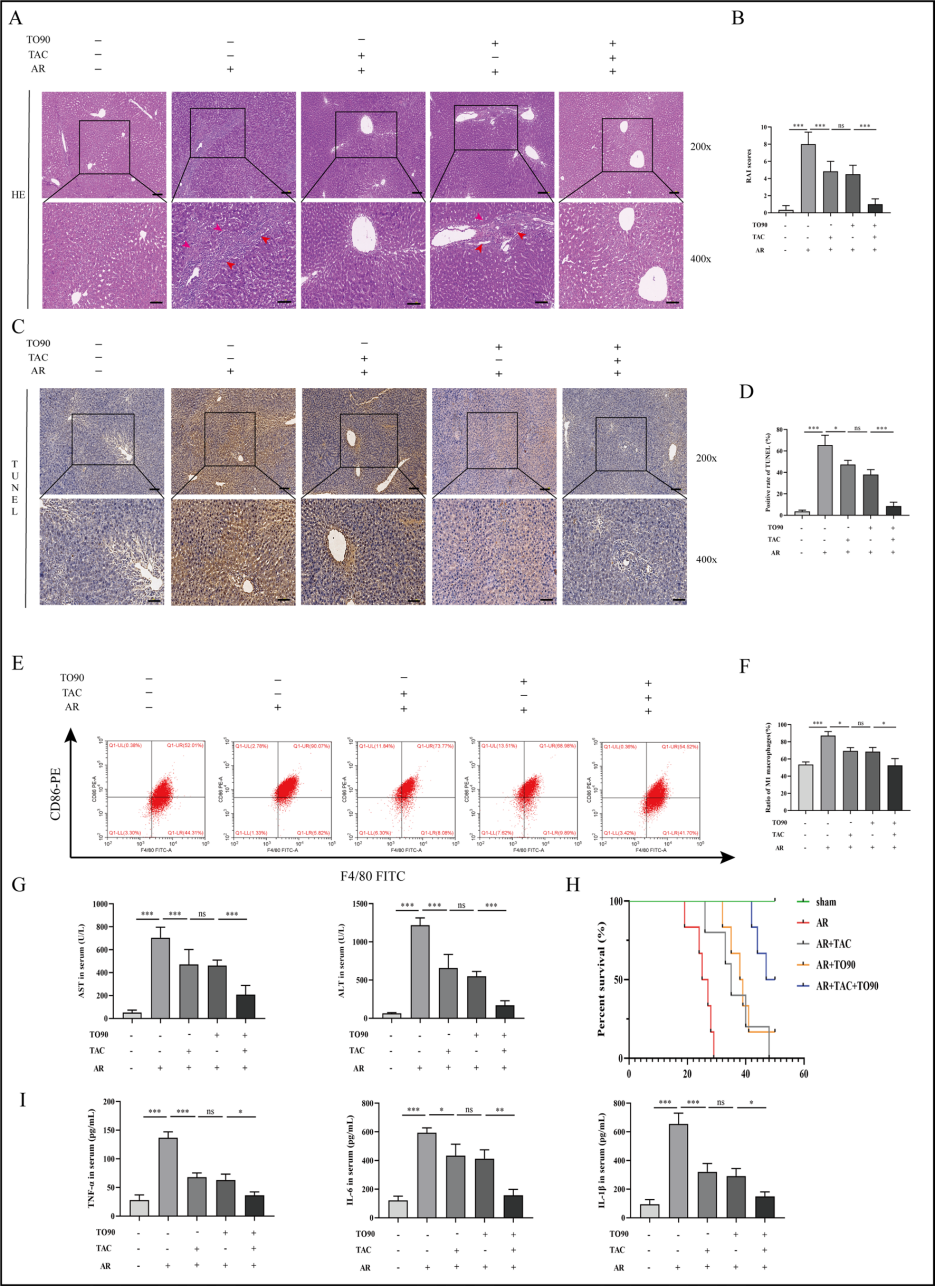
Effects of co-adjustment of TO901317 and tacrolimus on AR following liver transplantation in rats. **A**–**D** Hepatic pathological changes and apoptosis following co-adjustment with TO901317 and tacrolimus during AR (magnification, ×200; scale bar = 100 μm) (The area indicated by the red arrow shows inflammatory cell infiltration in the portal area and hepatic sinusoids, cholangitis and venulitis, as well as hepatic parenchymal cell damage). **E**, **F** The percentages of M1-type polarized macrophages in different groups were detected by flow cytometry, macrophages were separated with FITC-labeled F4/80, and M1-type polarization was separated with PE-labeled CD86. **G** ALT and AST levels in rat serum. **H** Survival rate of rats. n = 6 for each group. **I** Levels of TNF-α IL-6 and IL-1β in serum of different groups. Differences among groups were assessed by one-way ANOVA. n = 6, ^*ns*^*P* > 0.05, *P < 0.05, **P < 0.01, ***P < 0.001

The effects of TO90 and immunosuppressant tacrolimus on AR rats after LT were assessed via HE staining. TO90 and tacrolimus partially ameliorated liver injury compared with the AR group that received no treatment (Fig. 7A, B). However, combination therapy of TO90 and tacrolimus significantly improved liver injury, hepatocyte apoptosis, macrophage M1-polarization, liver function, and inflammatory factor secretion compared with individual treatments (Fig. 7A–I). The TO90+tacrolimus treatment group had better survival outcomes than other groups after LT (Fig. 7H). These findings suggest that TO90 combined with tacrolimus can significantly alleviate AR after LT.

## Discussion

Although LT represents the only effective treatment for ESLD (Bastos-Neves et al. 2019), shortage of donor livers, ischemia–reperfusion injury, AR, and even graft loss limit LT (Cvetkovski et al. 2021; Gao et al. 2023a). Prevention of AR after LT is crucial for patient prognosis (Montano-Loza et al. 2023; Lai et al. 2019). Accordingly, it is necessary to explore AR pathogenesis and innovative targets for AR treatment. Numerous studies have demonstrated that macrophage M1 polarization is a key event in AR after LT. Nonetheless, novel targets that can reduce M1 polarization of macrophages should be identified to alleviate AR and induce immune tolerance after LT (Liu et al. 2022).

LXR is a key regulator of inflammation, cholesterol metabolism, and functional regulation of macrophages. LXR can regulate macrophage function and anti-ischemia/reperfusion injury because it can modulate the expression of multiple genes (Zhao et al. 2021; Gao et al. 2023b; Glaría et al. 2020). In this study, LXRα expression in liver macrophages gradually reduced with AR aggravation. However, AR did not affect LXRβ expression, suggesting that LXRα may be a key regulator of macrophage function and AR after LT. In vitro experiments found that LXR α can inhibit the MAPK signal pathway through the ABCA1 pathway, thus reducing macrophage inflammation. Besides suppressing the inflammatory effect of macrophages, TO90 (LXRα agonist) can improve the pathological changes of AR after LT, reduce liver apoptosis, improve liver function, and prolong the survival time of AR rats.

**Fig. 8:**
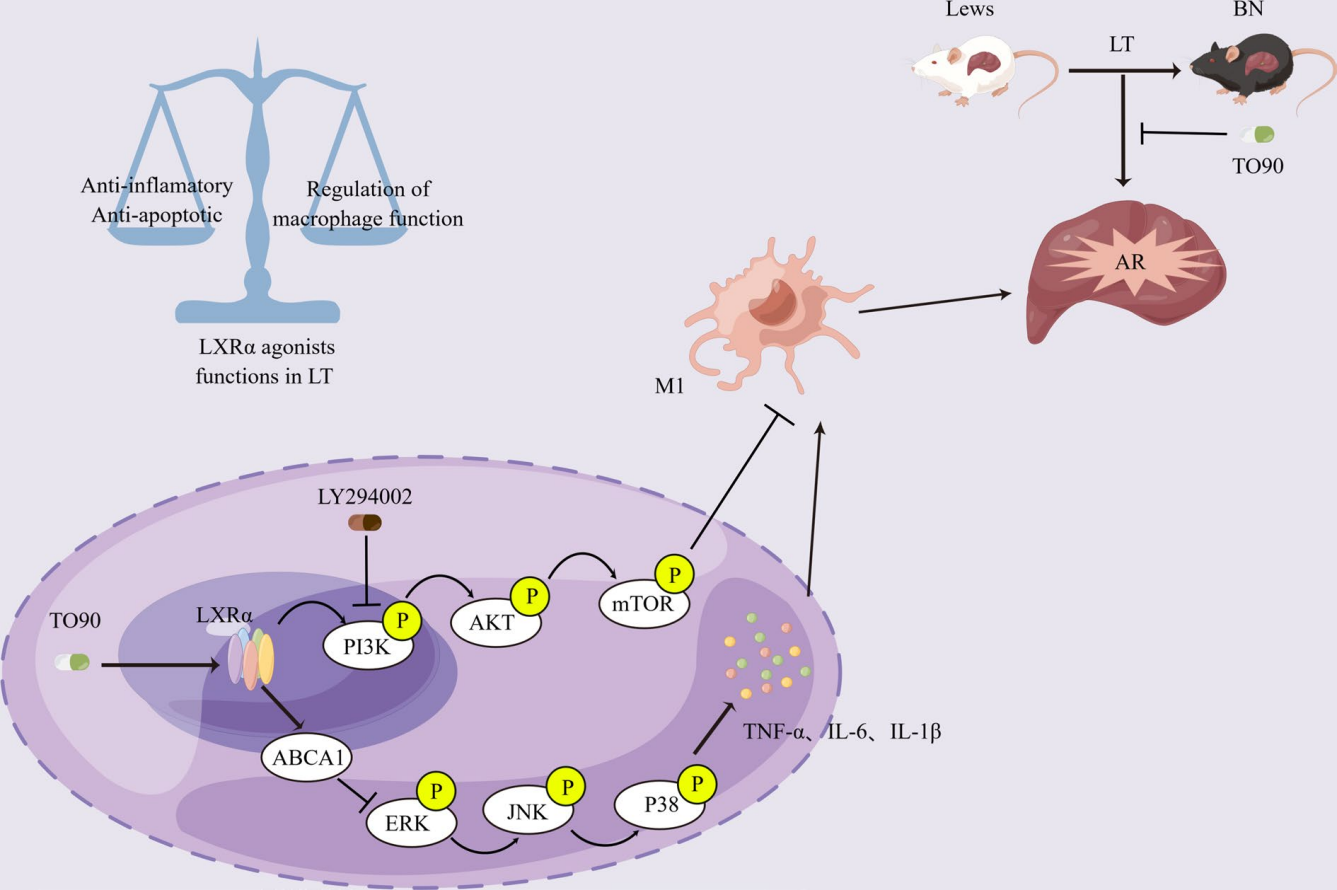
Schematic overview of mechanism. The proposed model shows that LXRα agonist (TO901317) reduce the release of pro-inflammatory factors via LXRα/ABCA1/MAPK signaling pathway and reduce M1 macrophages via activating the PI3K/AKT/mTOR signaling pathway. LXRα agonist is involved in various biological functions in LT, including anti-inflammatory, anti-apoptotic and regulation of macrophage function

Furthermore, LXRs can induce various anti-inflammatory effects in macrophages. Also, LXR activation suppresses acute hepatic inflammation through macrophage mediation (Endo-Umeda and Makishima 2019; Leussink et al. 2020). Miao et al. demonstrated that LXR agonists can repress IRF3 and NF-κB transcriptional activity and inhibit LPS-induced inflammatory response in Kupffer cells (Miao et al. 2016). Wang et al. have reported that LXR agonists can modulate posttranscriptional TNF-α and p38 mitogen-activated protein kinase activation in liver macrophages (Wang et al. 2009). Nonetheless, further studies should assess the potential signaling pathways connecting LXR and target genes responsible for governing macrophage polarization. Moreover, functional status is crucial for elucidating the precise mechanisms through which LXR regulates AR after LT. Herein, results suggested that LXRα/ABCA1/MAPK axis participates in the inflammatory response in macrophages. Moreover, the TO90 treatment alleviated AR after LT. However, ABCA1 knockdown reversed these changes. Interestingly, the LXRα agonist regulated the mTOR signal pathway and inhibited the M1 polarization of macrophages. However, ABCA1 inhibition did not affect the polarization of macrophages, suggesting that the effect of LXRα on macrophage M1 polarization may not depend on the LXRα/ABCA1 axis. Furthermore, LY294002 (PI3K inhibitor) t partially restored the inhibitory impact of TO90 on M1 polarization of macrophages and alleviated AR after LT. These findings suggest that TO90 may regulate macrophage polarization by modulating the PI3K/AKT/mTOR pathway. A concept diagram depicts our major findings (Fig. 8).

Tacrolimus, as a calcineurin inhibitor, is widely employed for preventing liver transplant rejection (Park et al. 2023; Wu et al. 2018). Nonetheless, its excessive usage is related to many serious side effects, including diabetes mellitus, chronic allograft nephropathy, neurotoxicity, and arterial hypertension. Many studies have found that using multi-target and multi-pathway methods for AR treatment after LT can reduce the dosage and time of traditional immunosuppressants. In this study, TO90 combined with tacrolimus was more effective in relieving AR after LT than either of the treatments alone. Multi-target treatment may be more effective in alleviating AR after LT. However, this study has some shortcomings. First, this study lacks validation using clinical samples. Second, the mechanism by which LXRα affects AR after LT was not assessed via integrated multi-omics analysis.

In conclusion, LXRα agonists can inhibit inflammation through the ABCA1/MAPK pathway and M1 polarization of macrophages by inhibiting PI3K/AKT/mTOR. Also, LXRα agonist can significantly reduce AR after LT. The combination of LXRα agonist and tacrolimus can alleviate AR after LT, indicating that LXRα may become an innovative target for AR diagnosis and treatment after LT.

## Supplementary Information

Additional file 1.

## Data Availability

The data that support the findings of this study are available from the corresponding author upon request.
