# Supplementary material for: LXRα agonists ameliorates acute rejection after liver transplantation via ABCA1/MAPK and PI3K/AKT/mTOR signaling axis in macrophages
Source: Mol Med. 2025 Mar 14;31:99. doi: 10.1186/s10020-025-01153-1 (PMC11908107; doi:10.1186/s10020-025-01153-1)
Supplement: Supplementary file 1 — Additional file 1. [file 10020_2025_1153_MOESM1_ESM.docx]

**Uncropped original western blots**

**Fig. 1B liver macrophage**

**

LXRα**

**
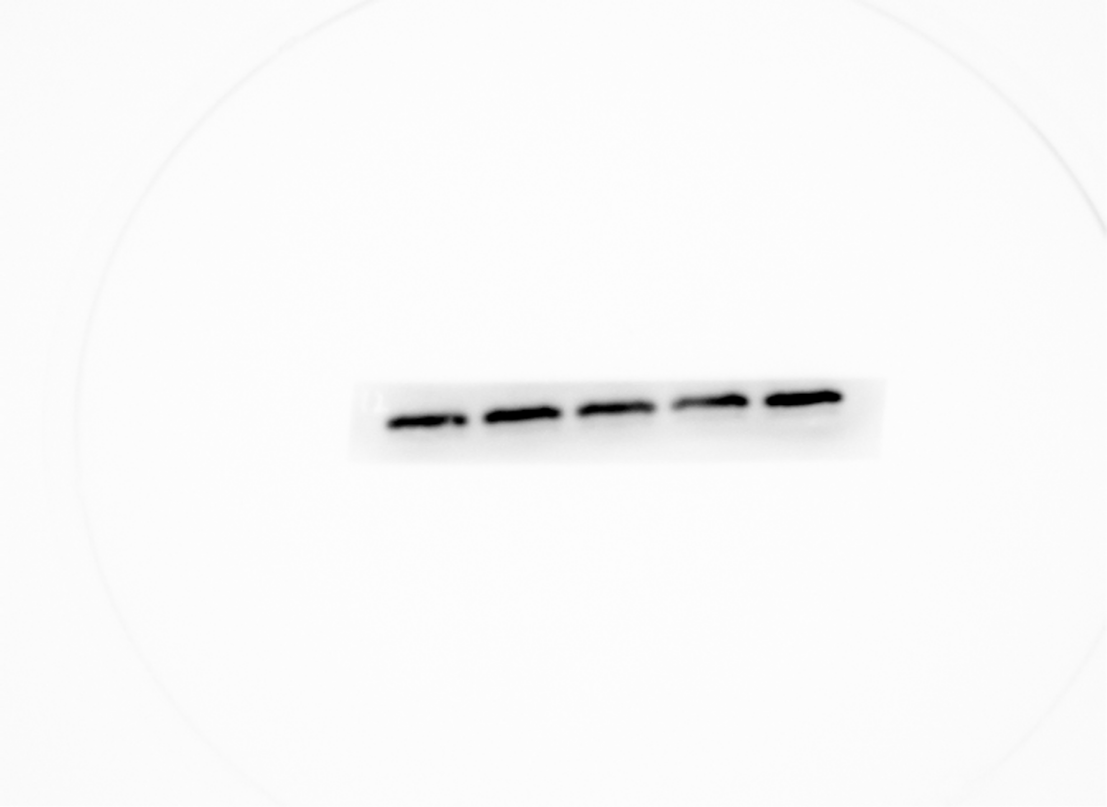
LXRβ**

**

ABCA1**

**

ABCG1**

**
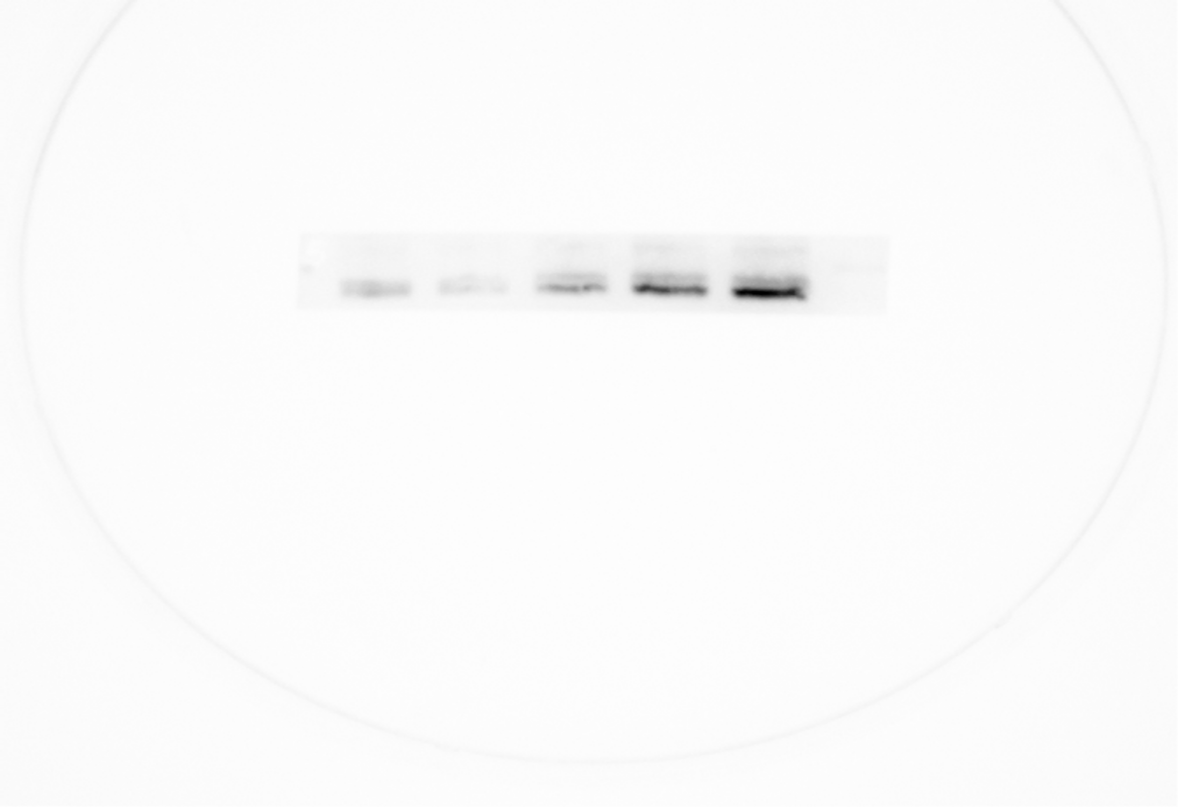
P-ERK1/2**

**
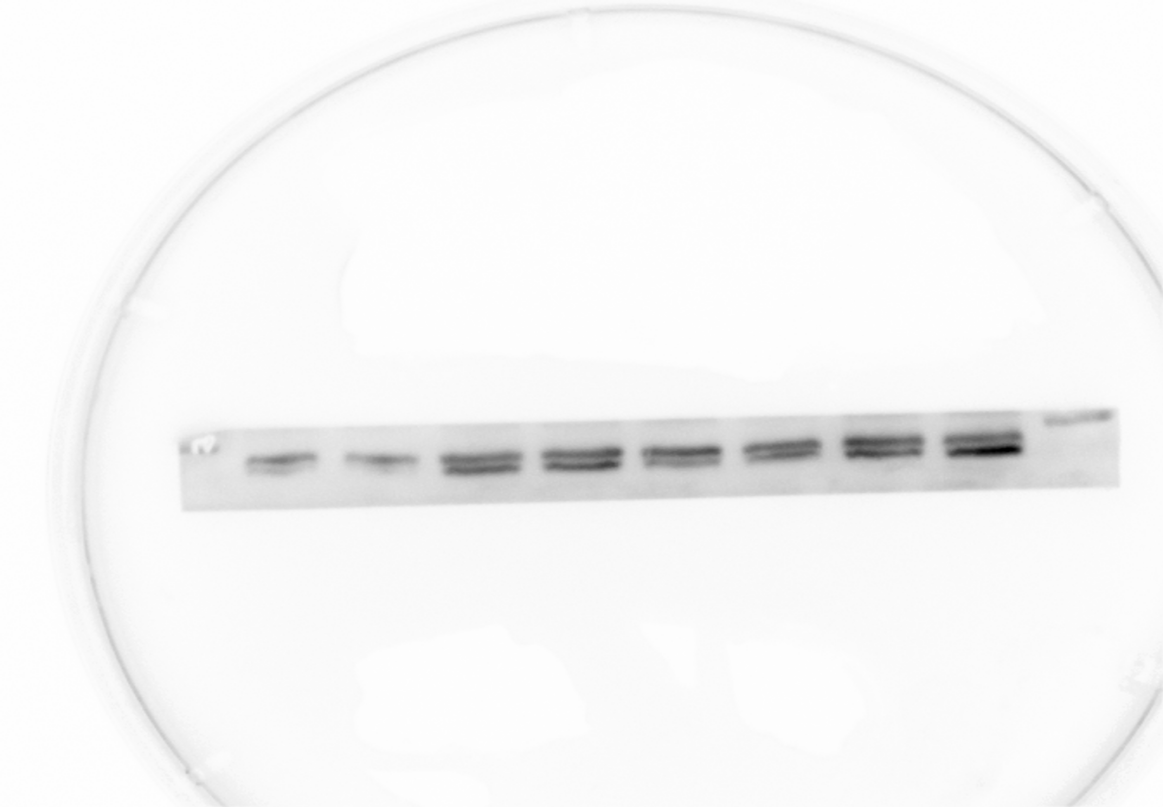
ERK1/2**

**

P-JNK**

**
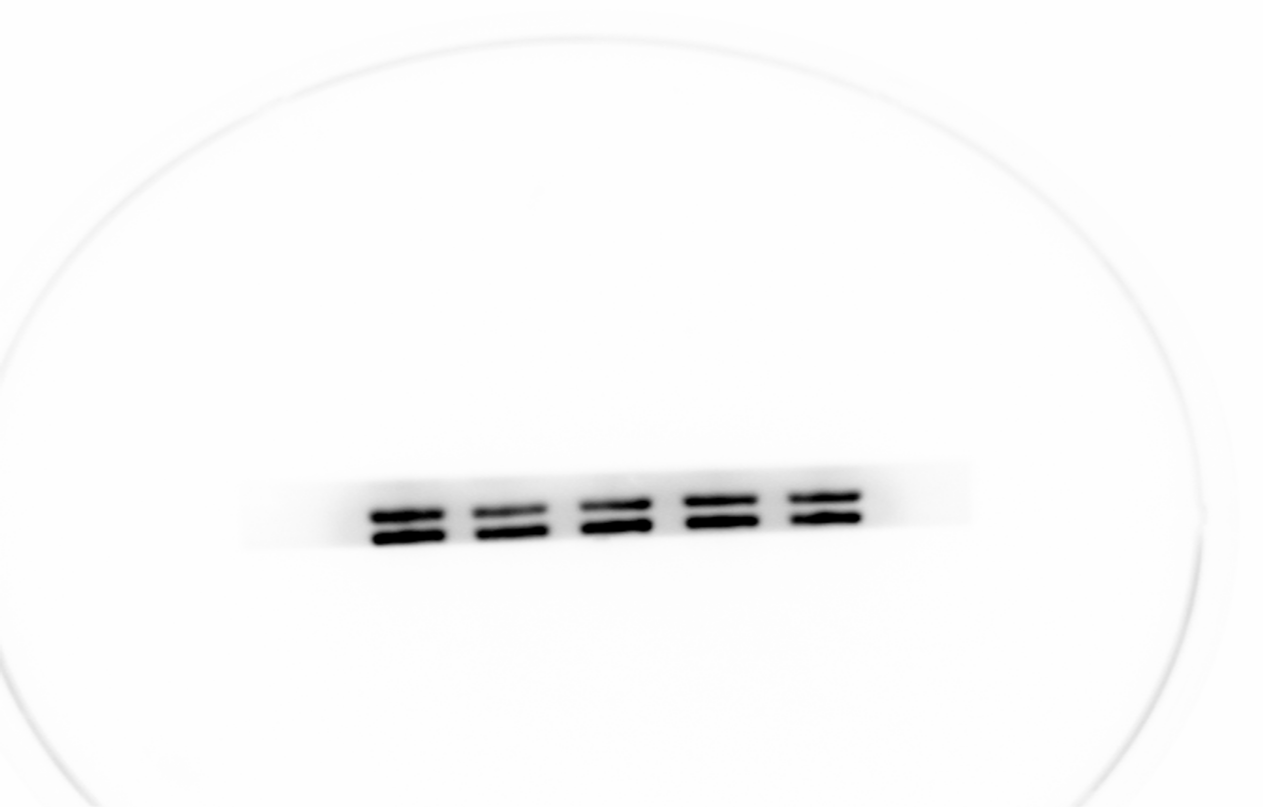
JNK**

**
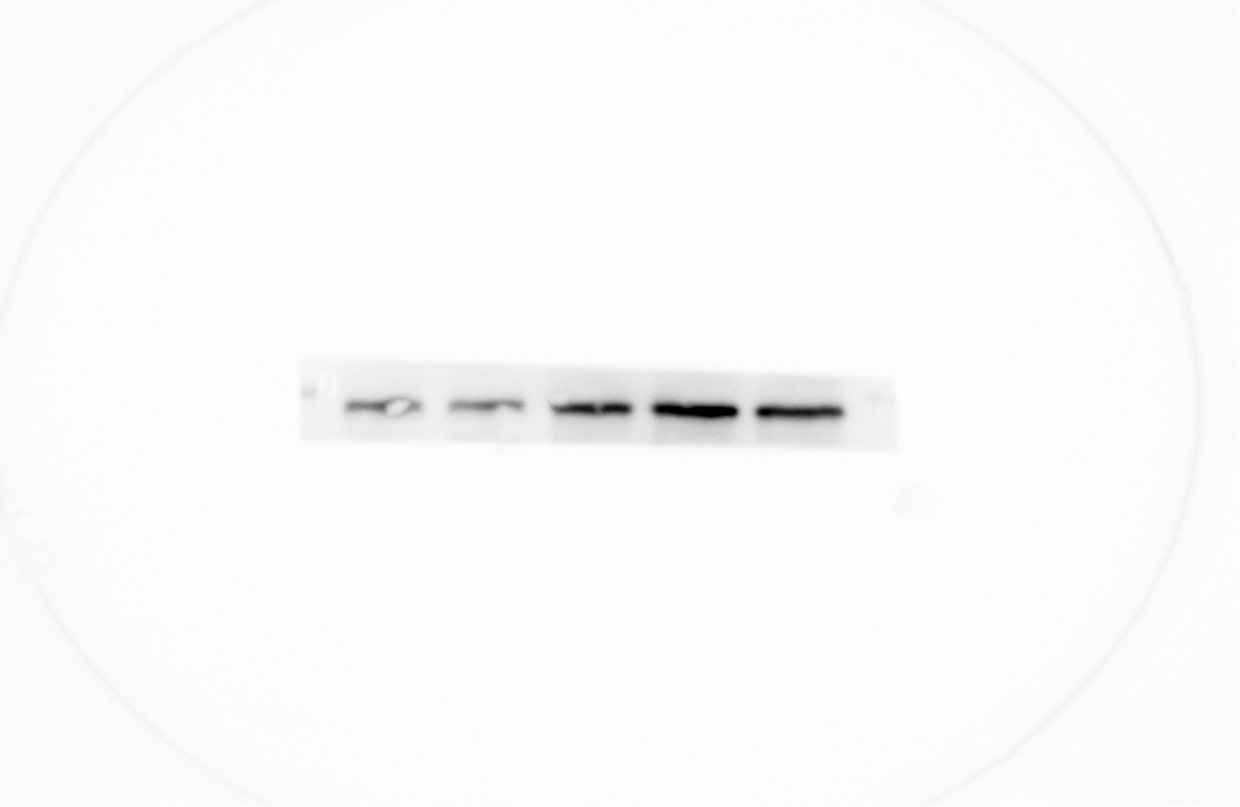
P-P38**

**
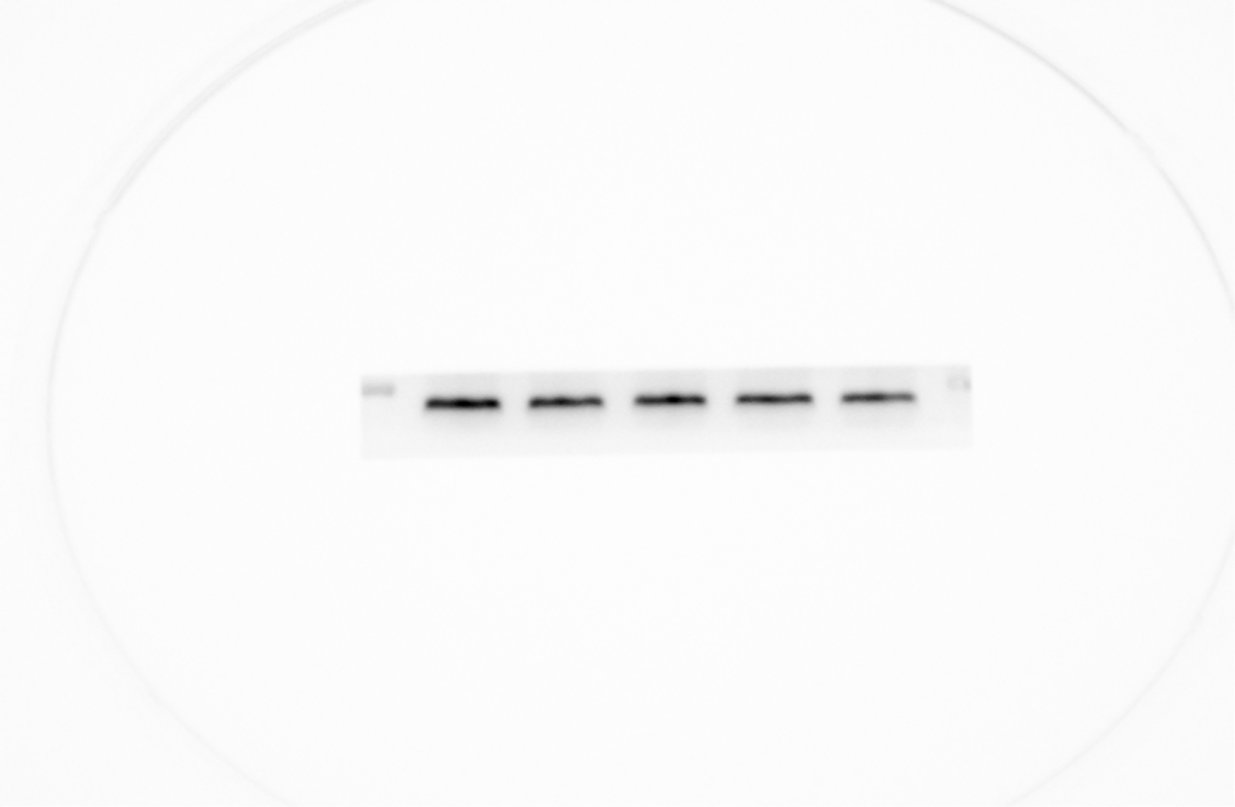
P38**

**
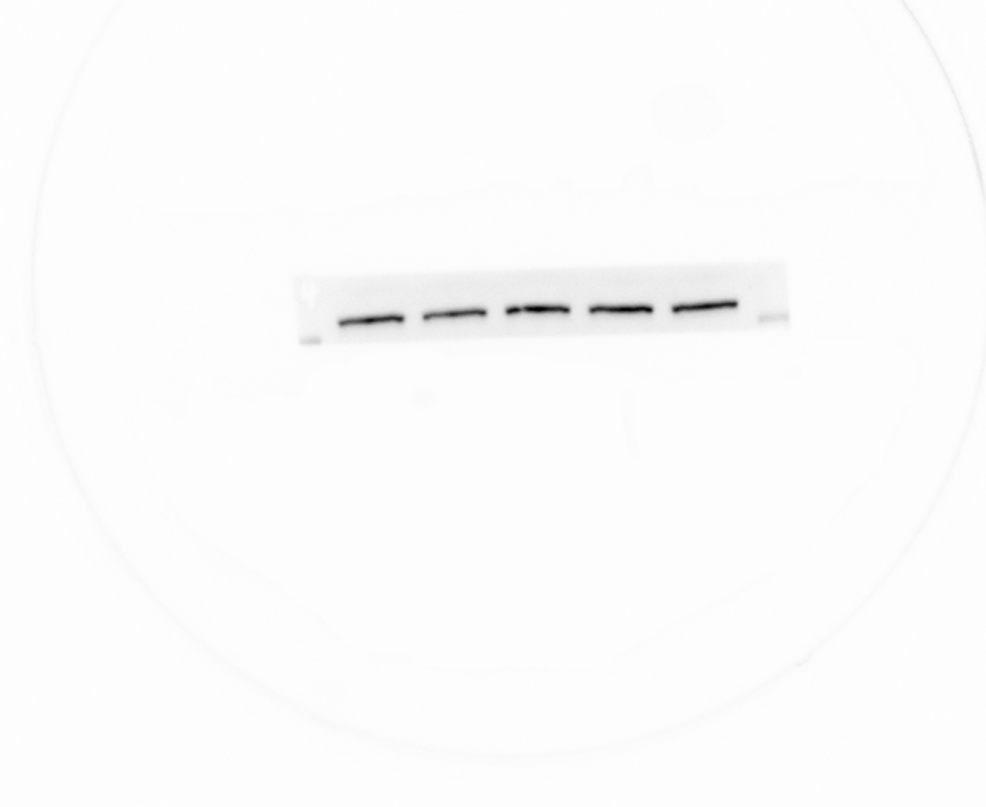
β-actin**

**
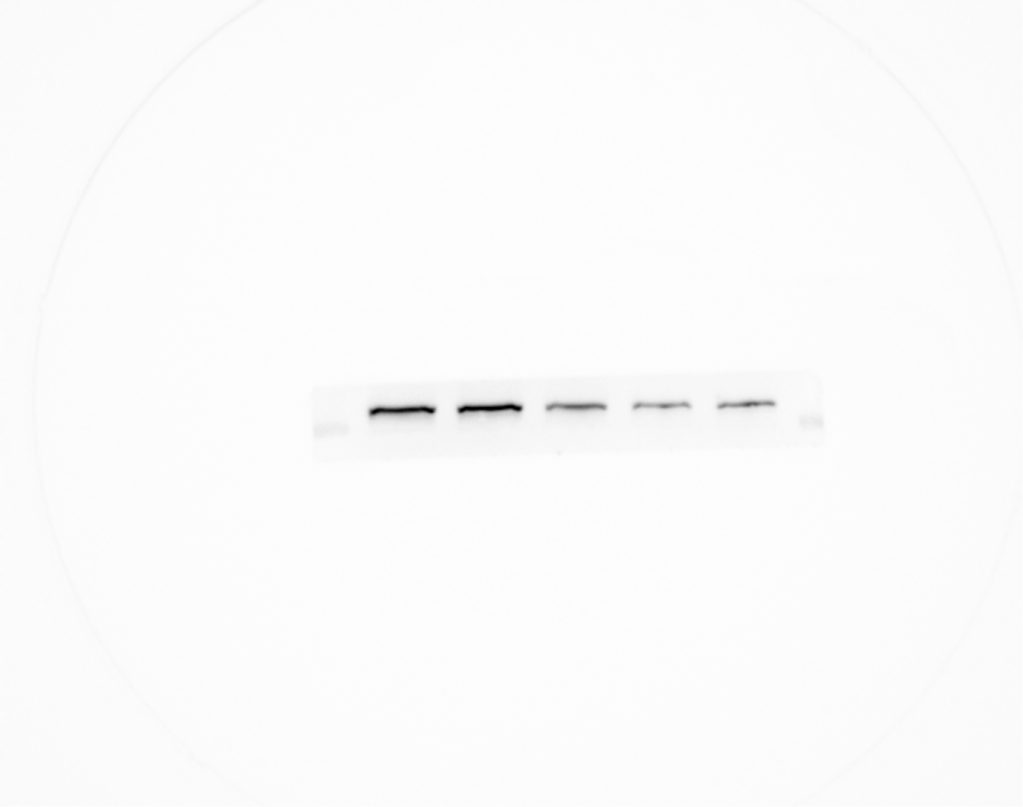
P-AKT**

**
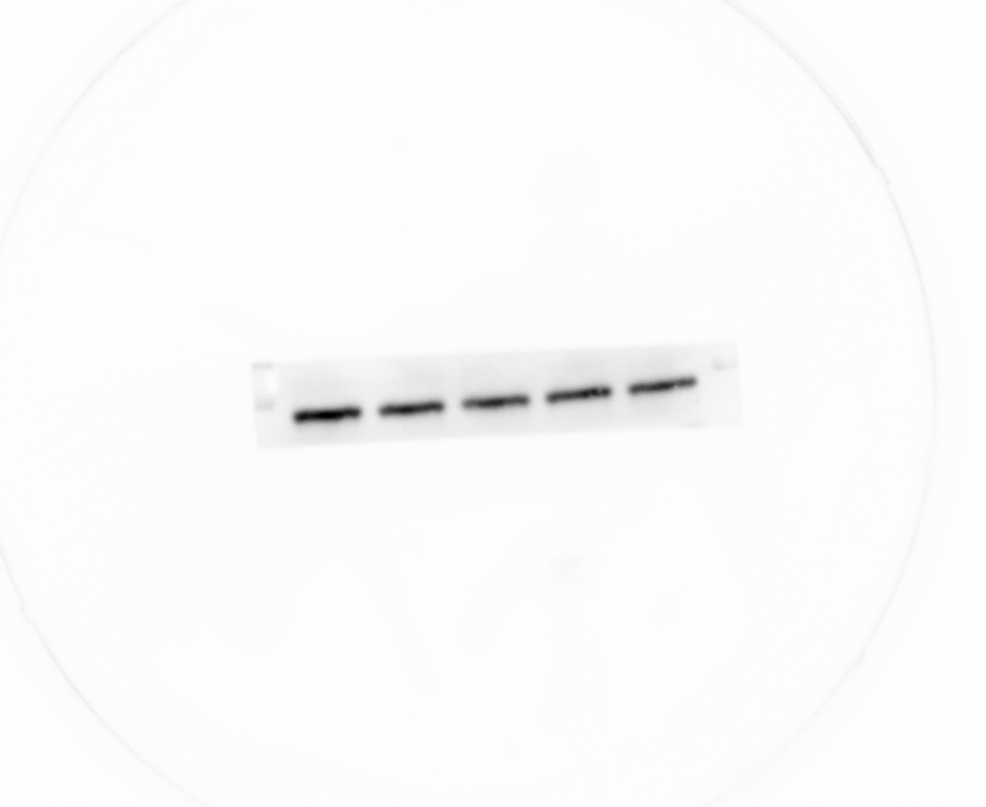
AKT**

**

P-mTOR**

**
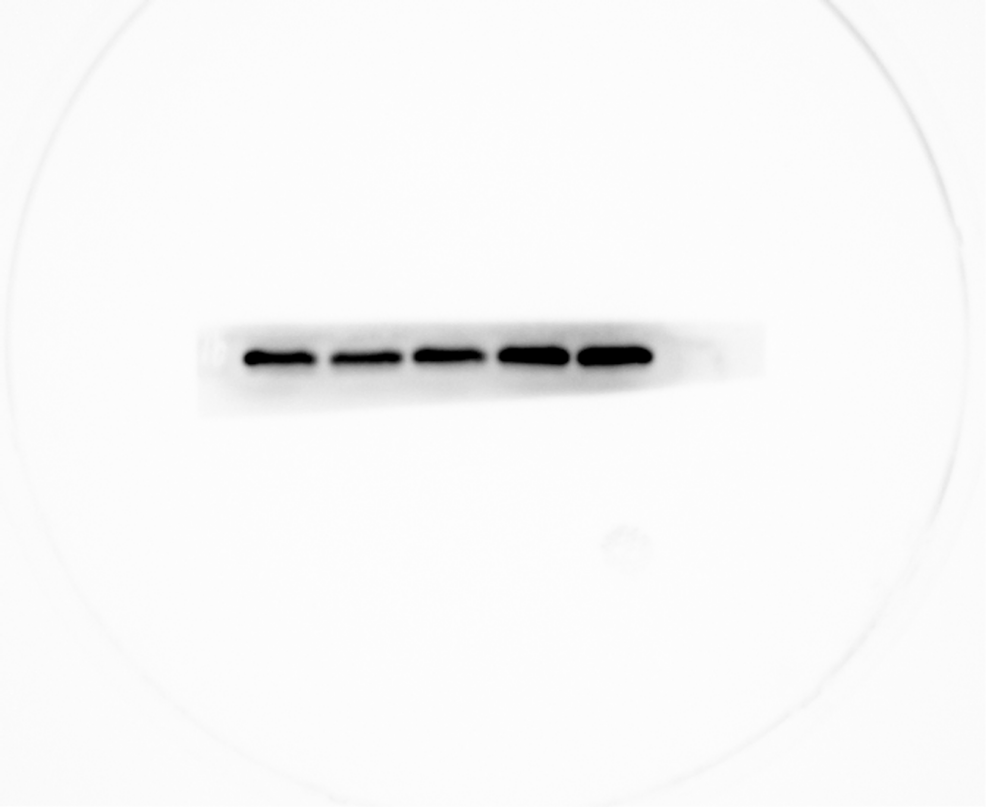
mTOR**

**
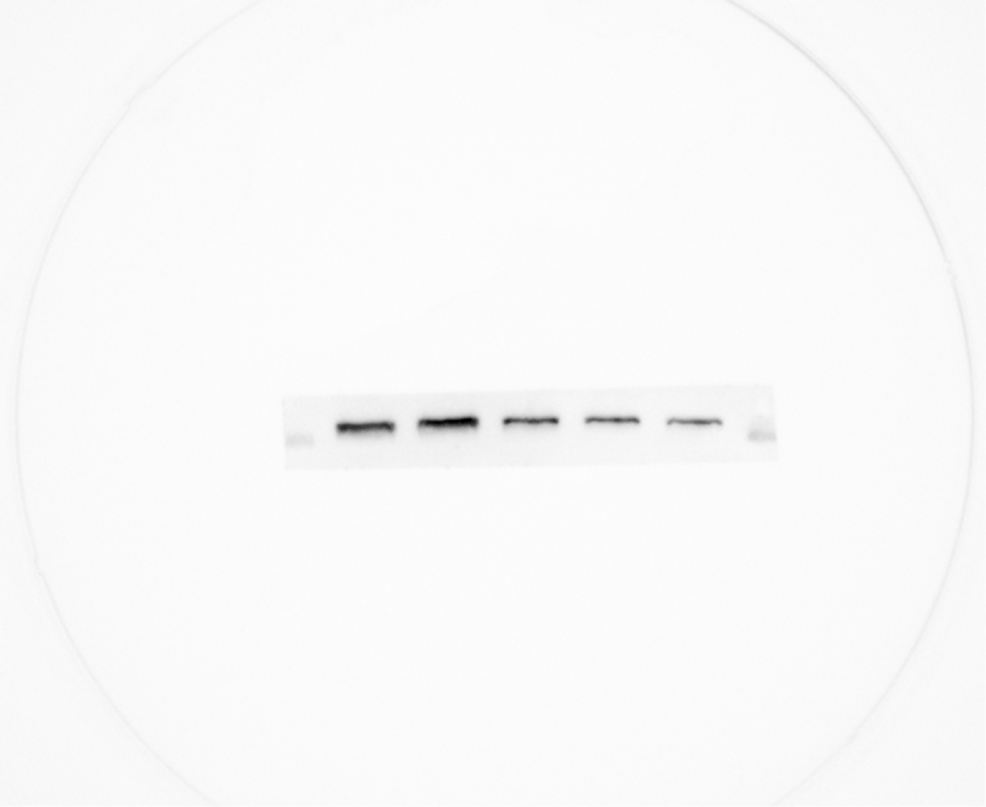
P-S6K**

**
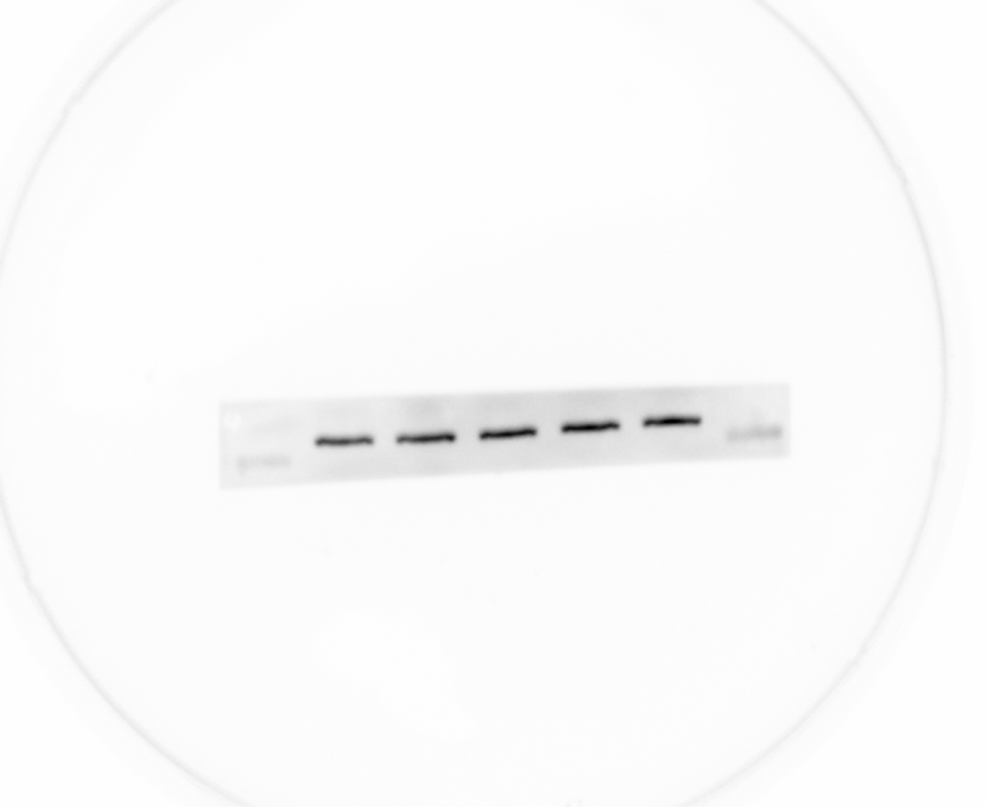
S6K**

**
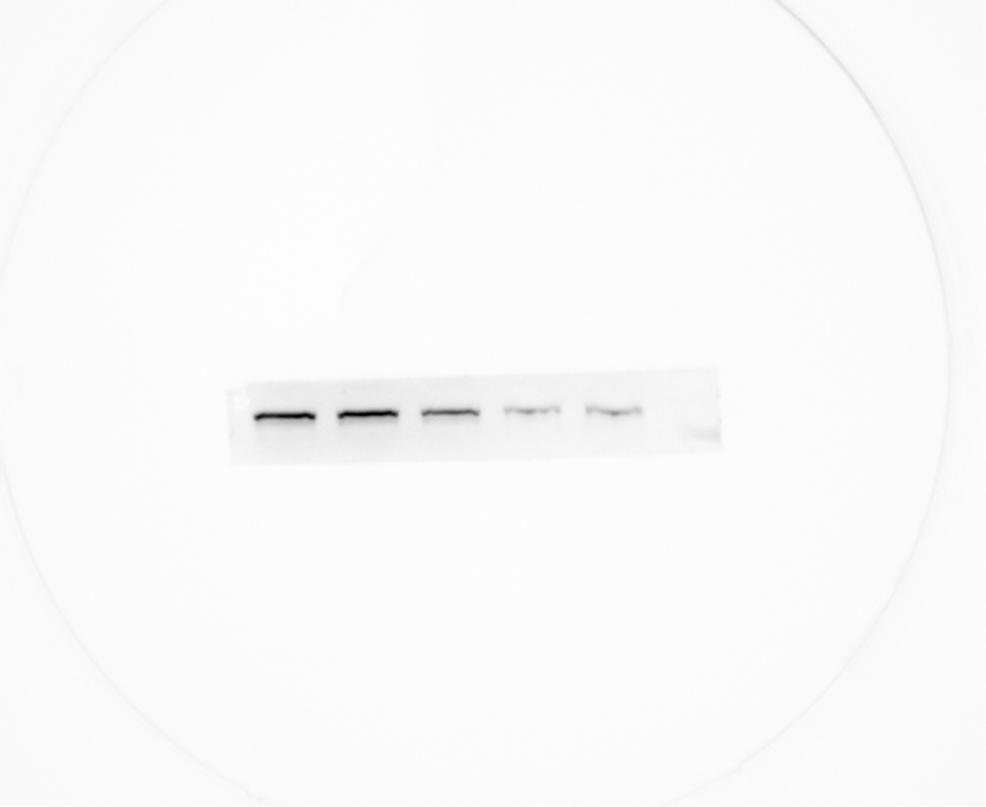
P-4E-BP**

**
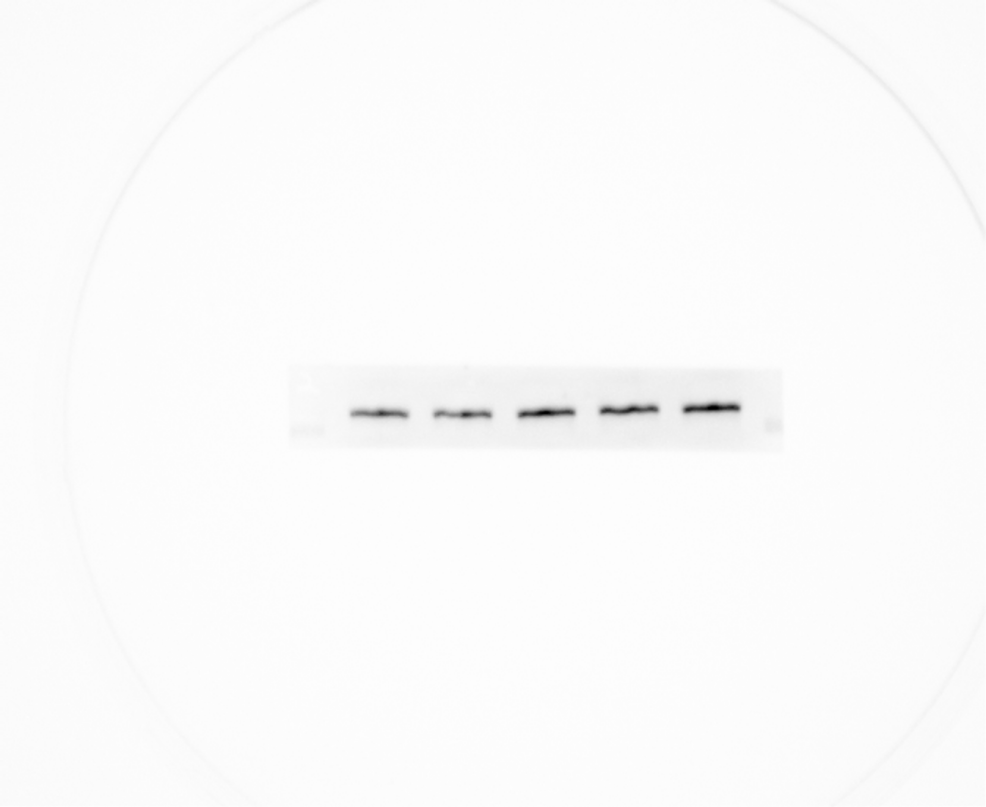
4E-BP**

**
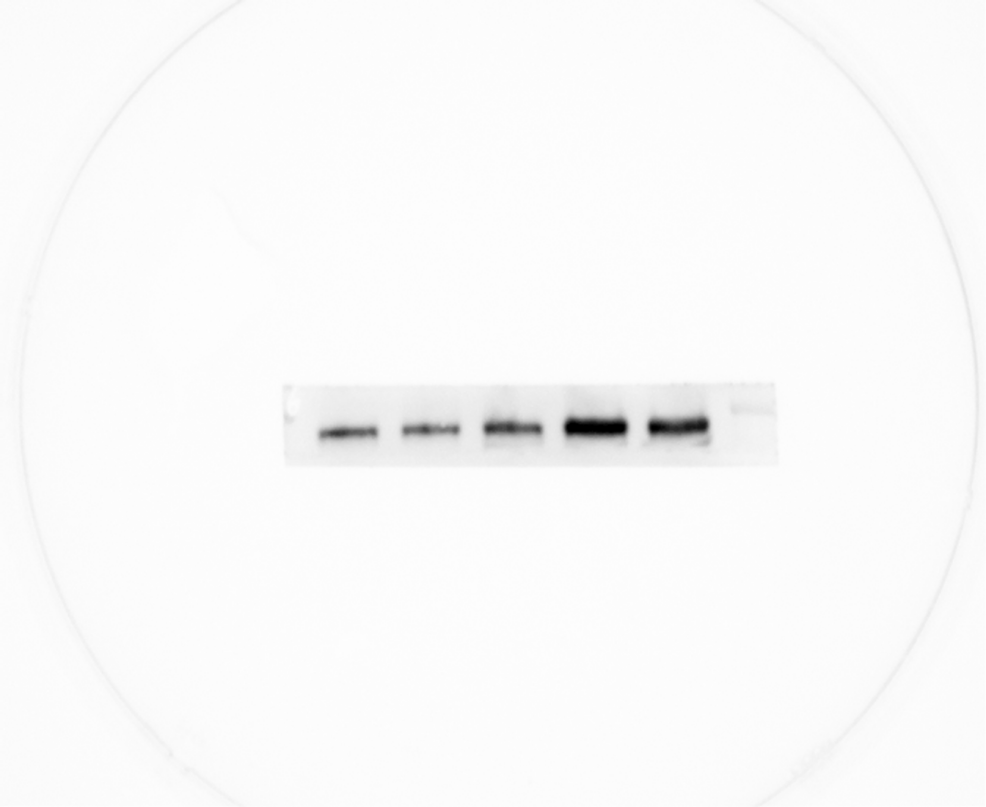
IL-6**

**
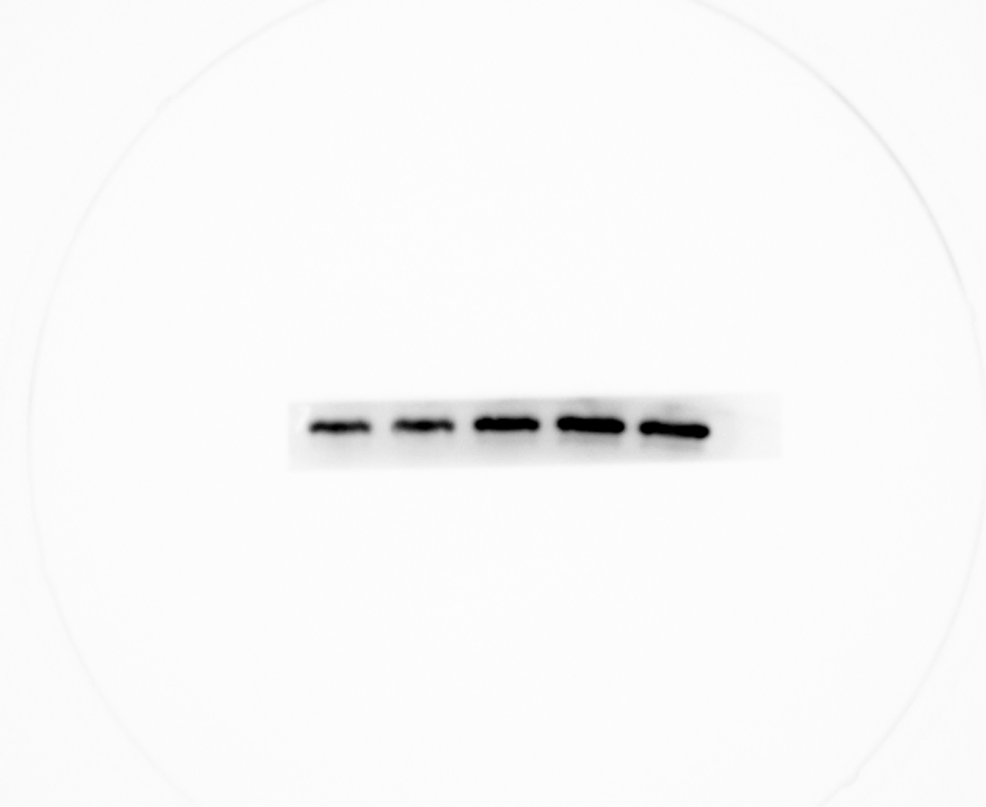
TNF-α**

**
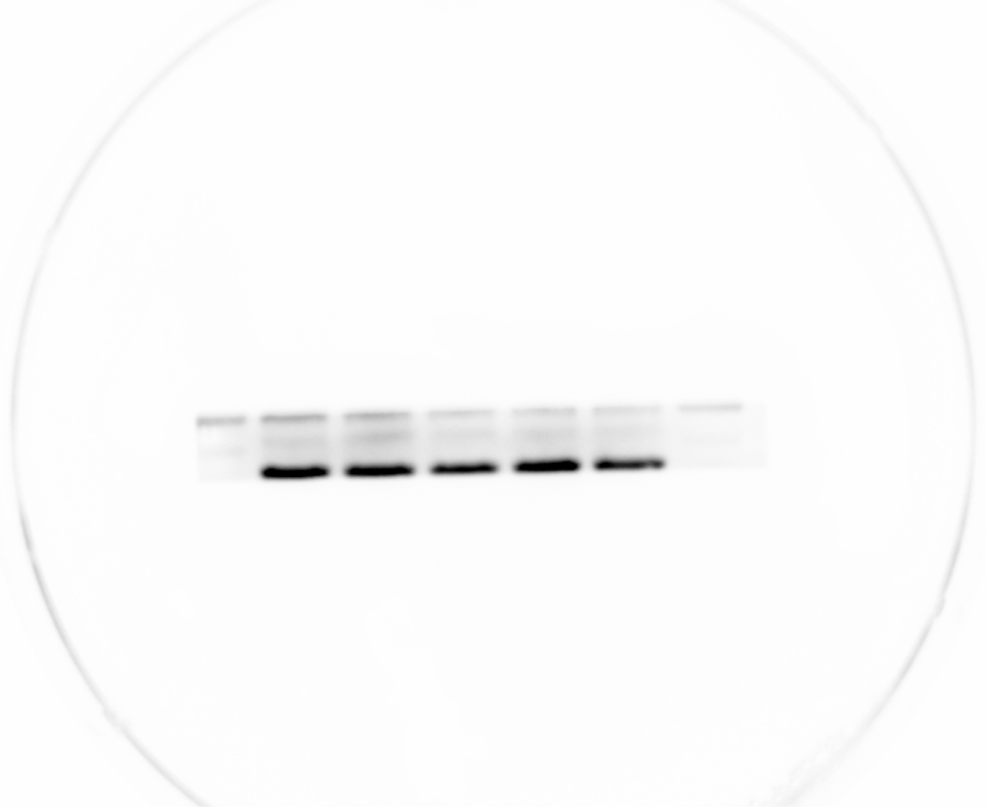
β-actin**

**Fig. 2A liver macrophage**

**

LXRα**

**

ABCA1**

**
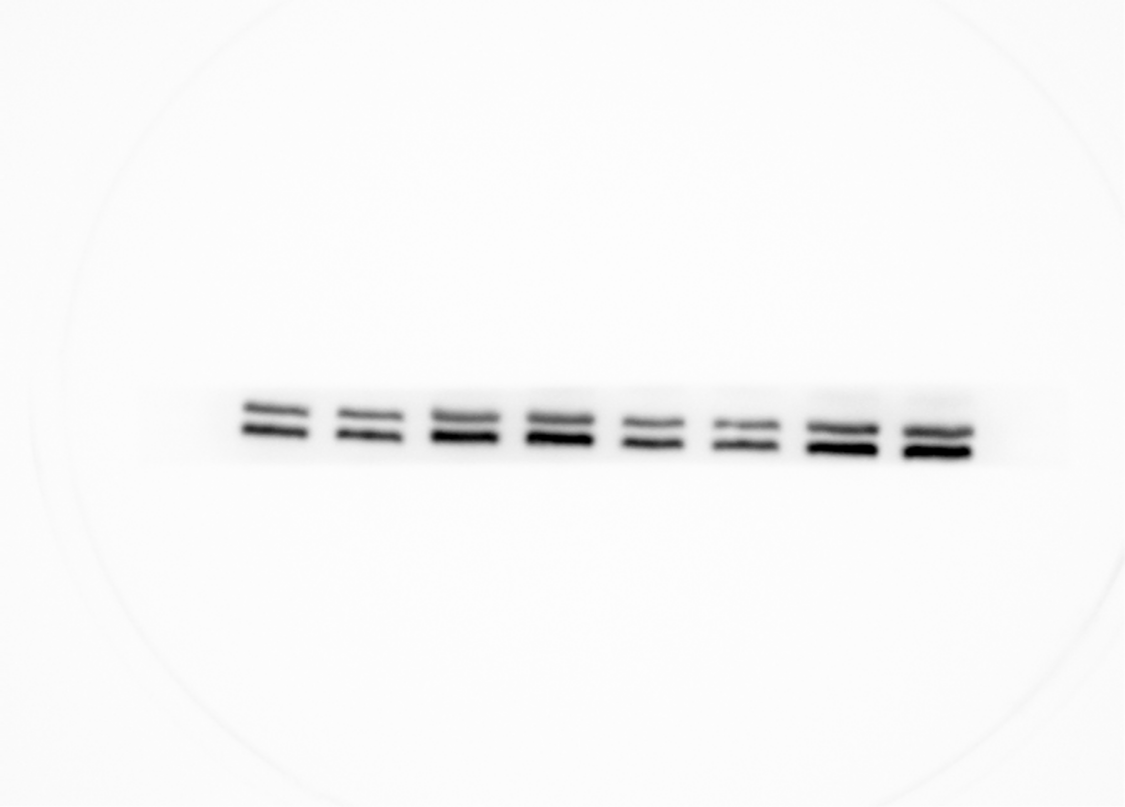
P-ERK1/2**

**
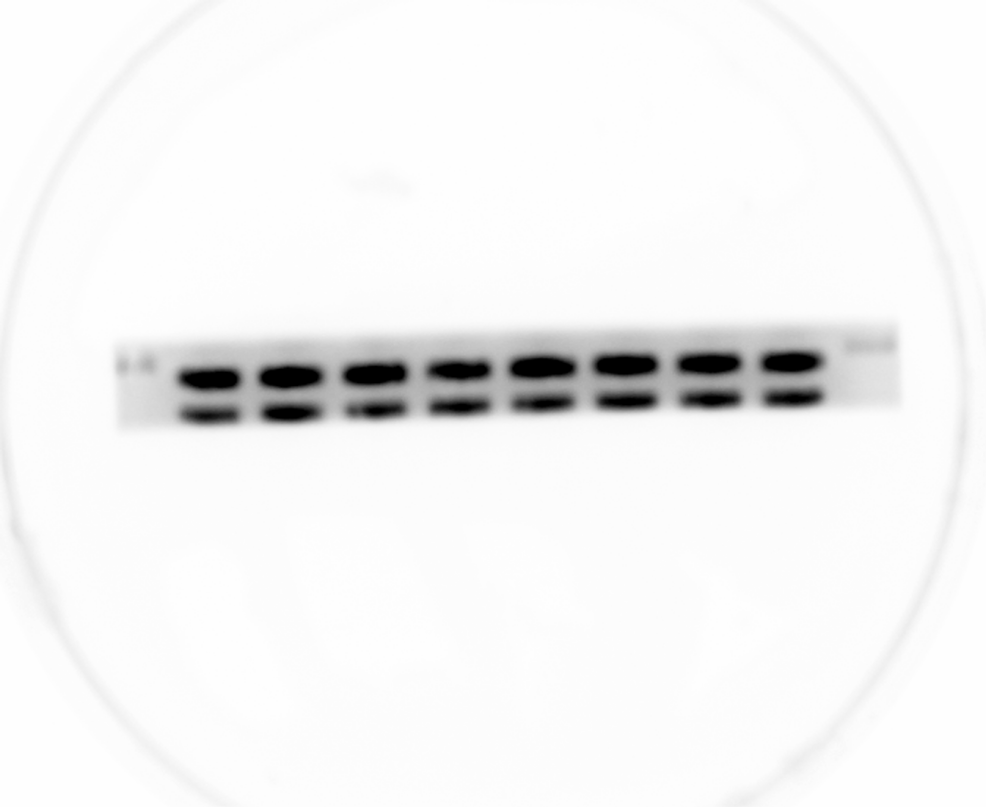
ERK1/2**

**
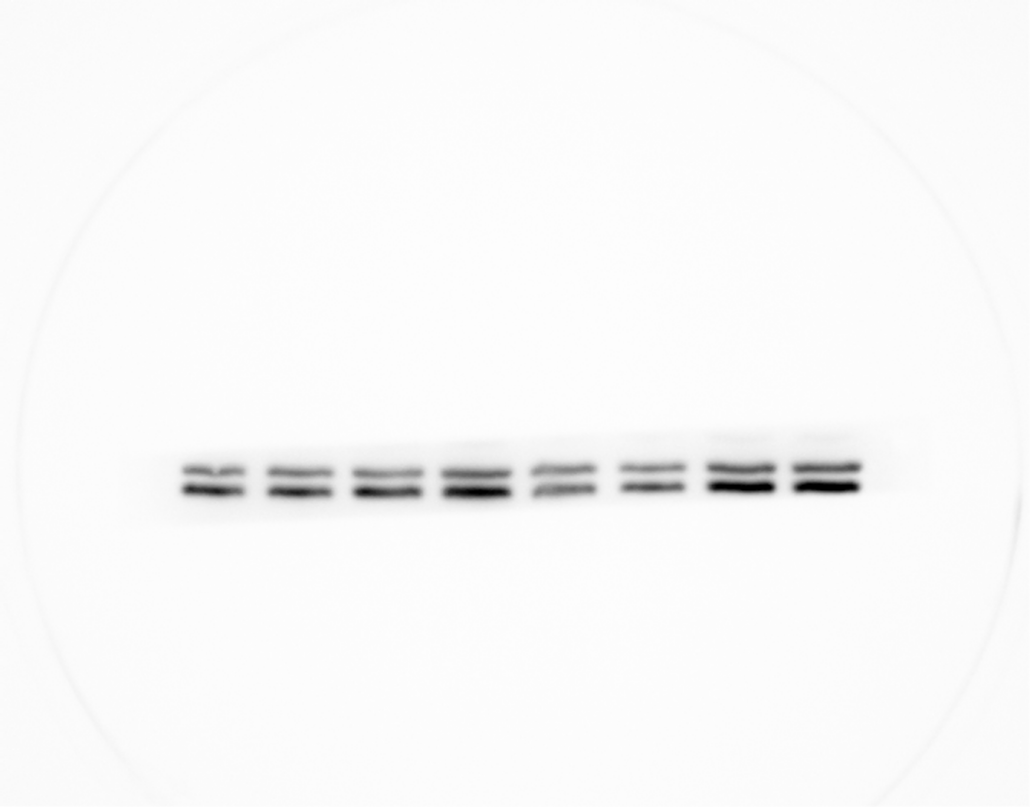
P-JNK**

**
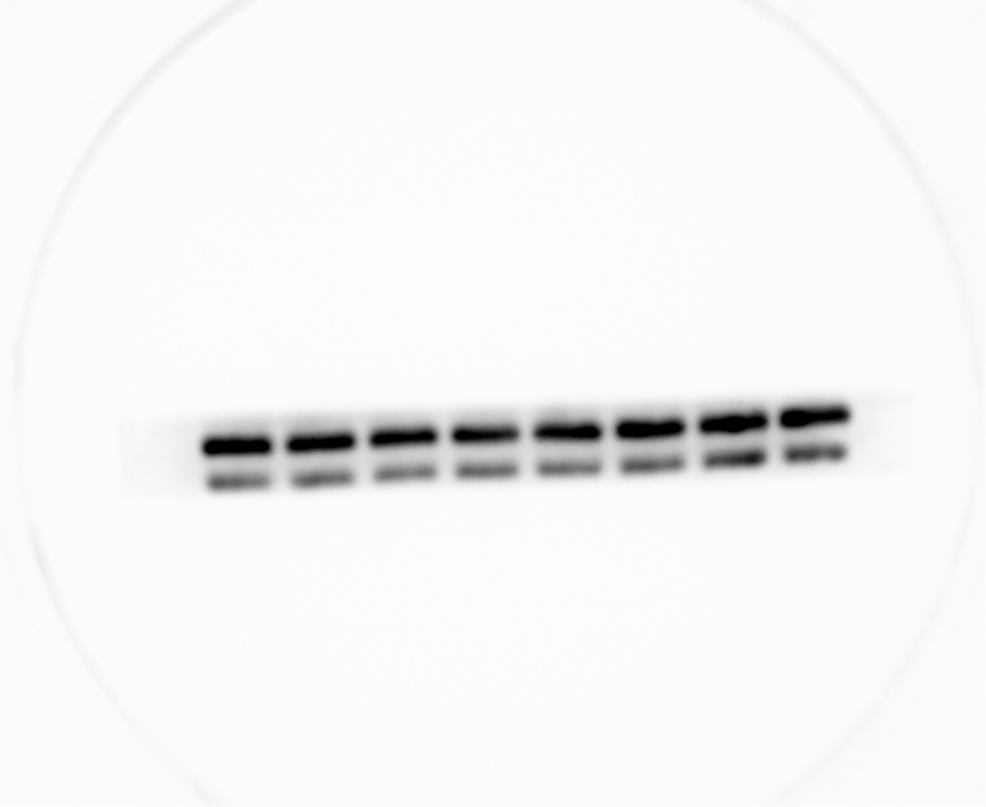
JNK**

**
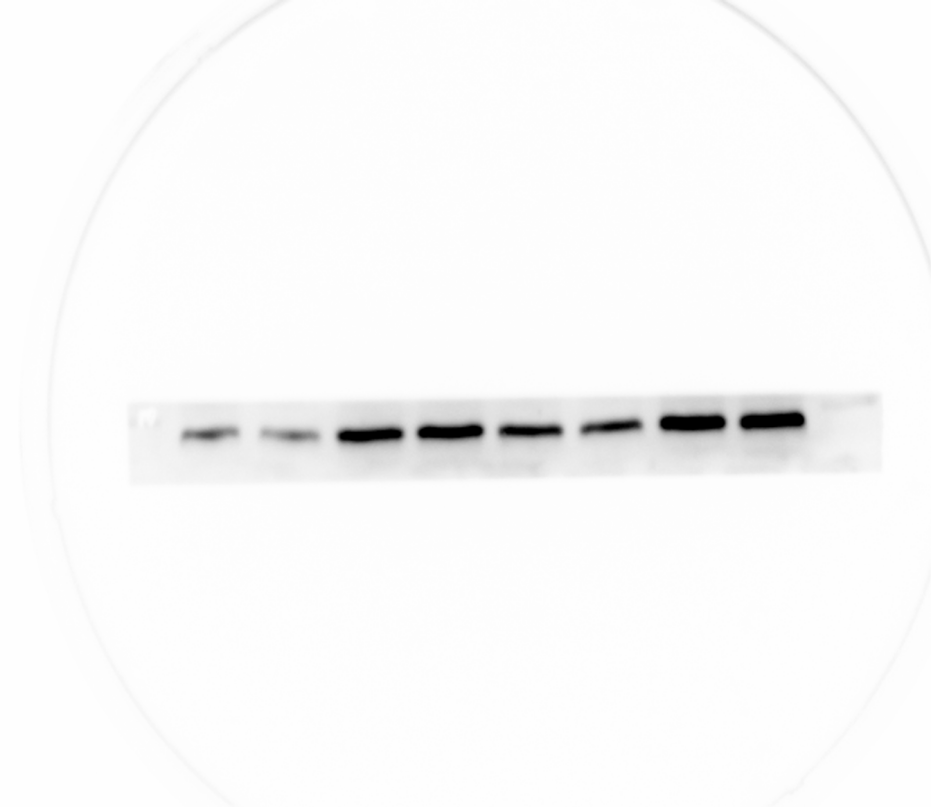
P-P38**

**
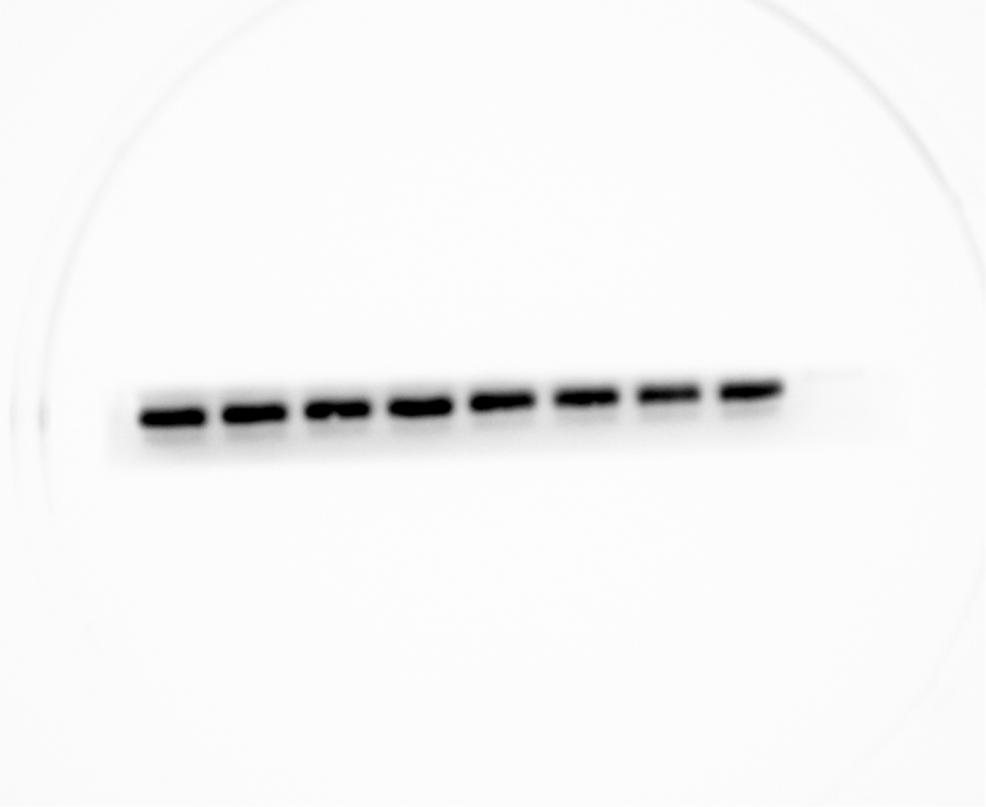
P38**

**
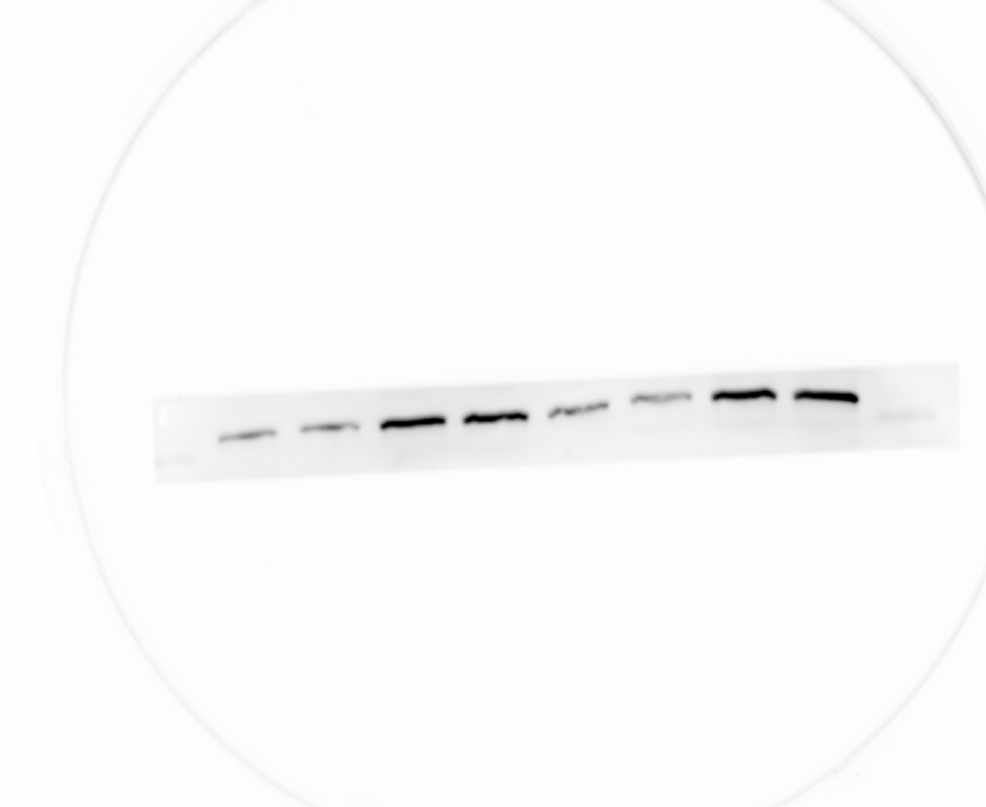
IL-6**

**
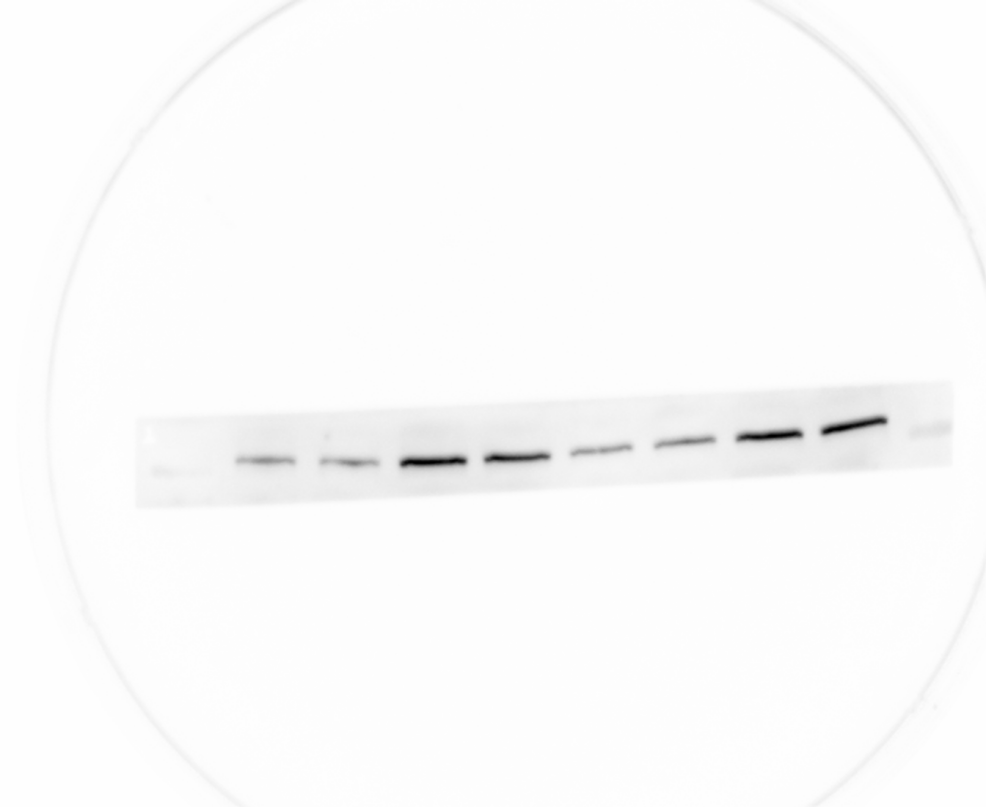
TNF-α**

**
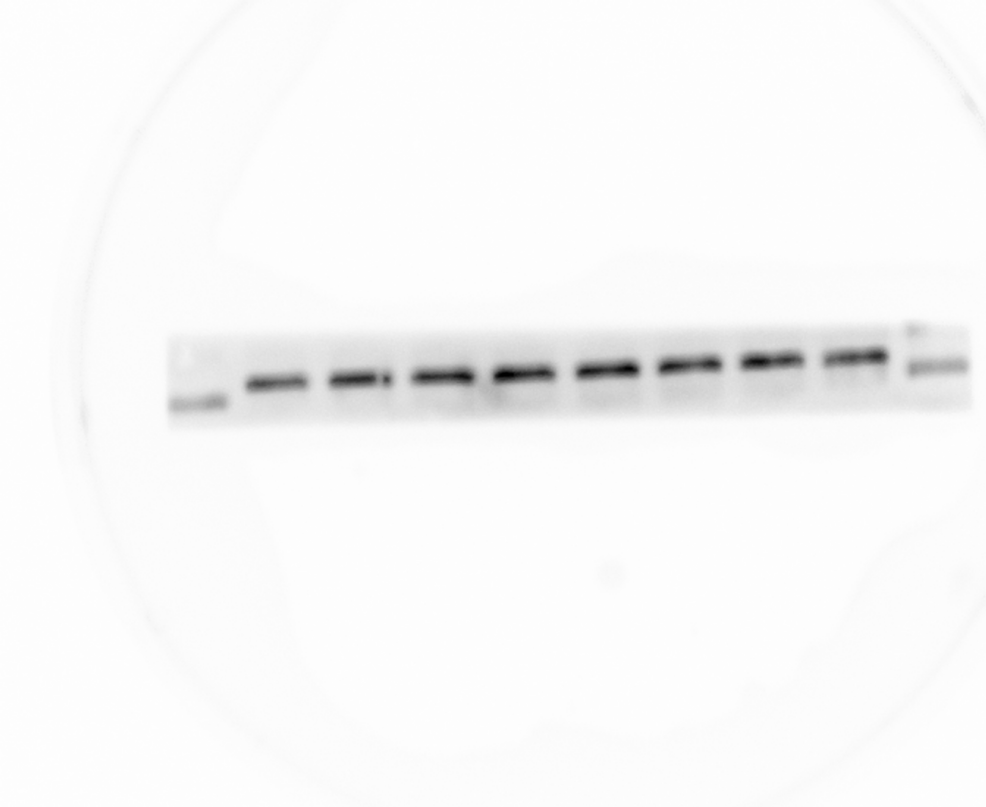
β-actin**

**Fig. 4C liver tissues**

**
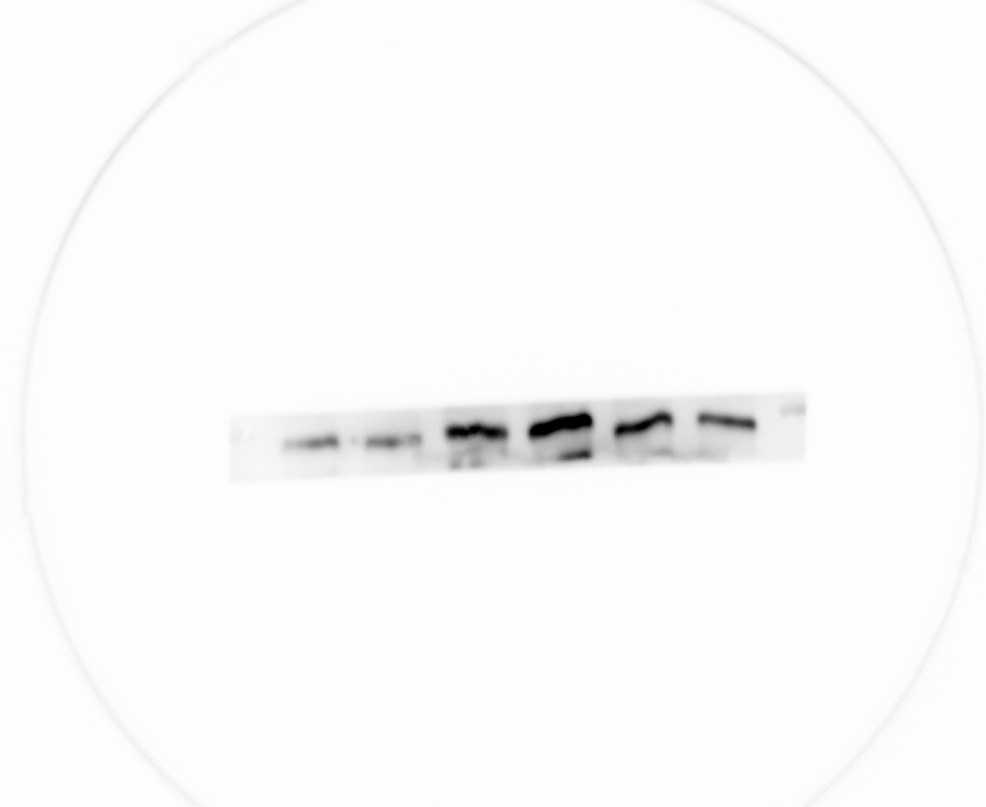
Cleaved Caspase3**

**
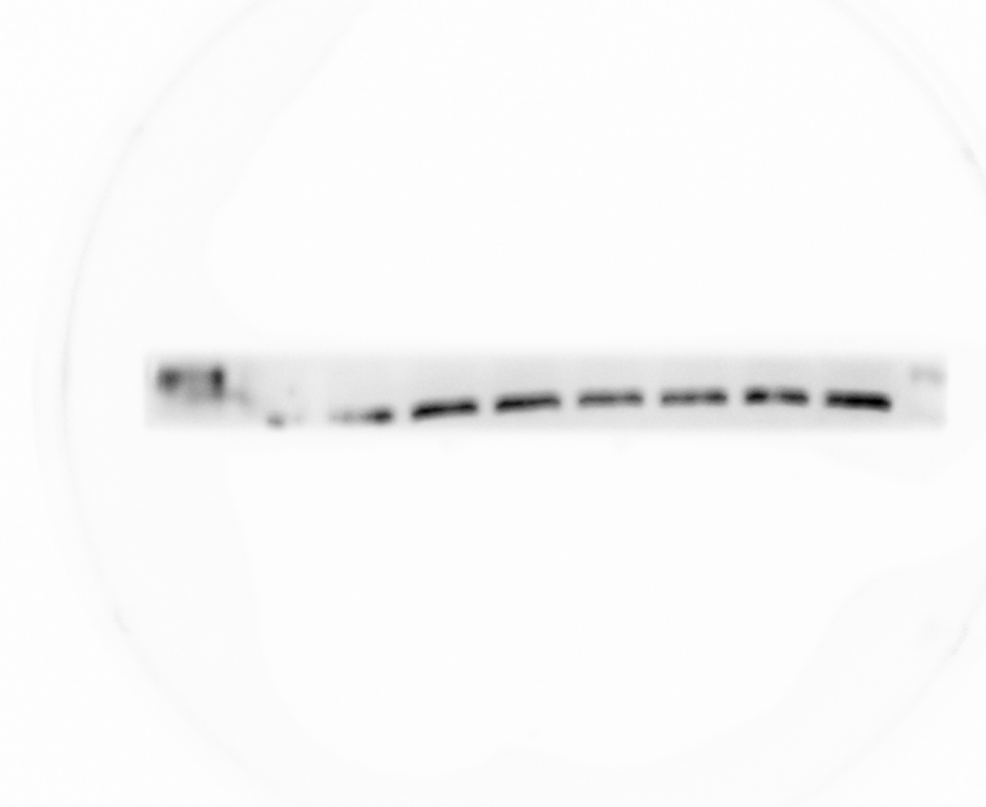
Caspase3**

**
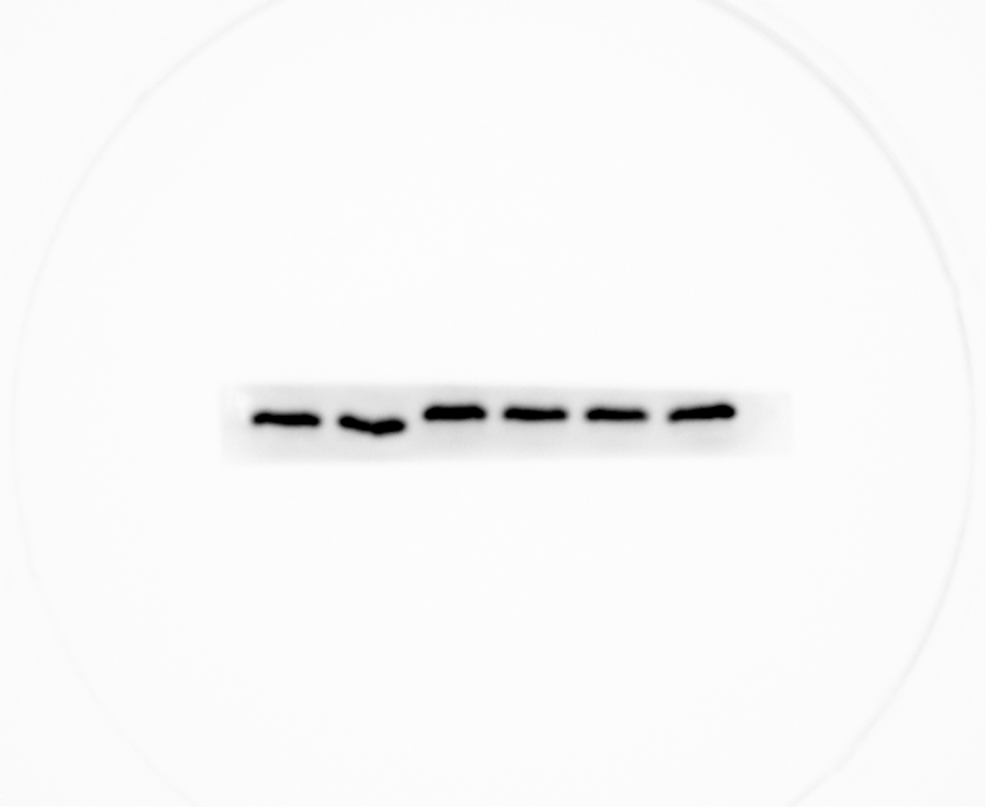
Cleaved Caspase6**

**
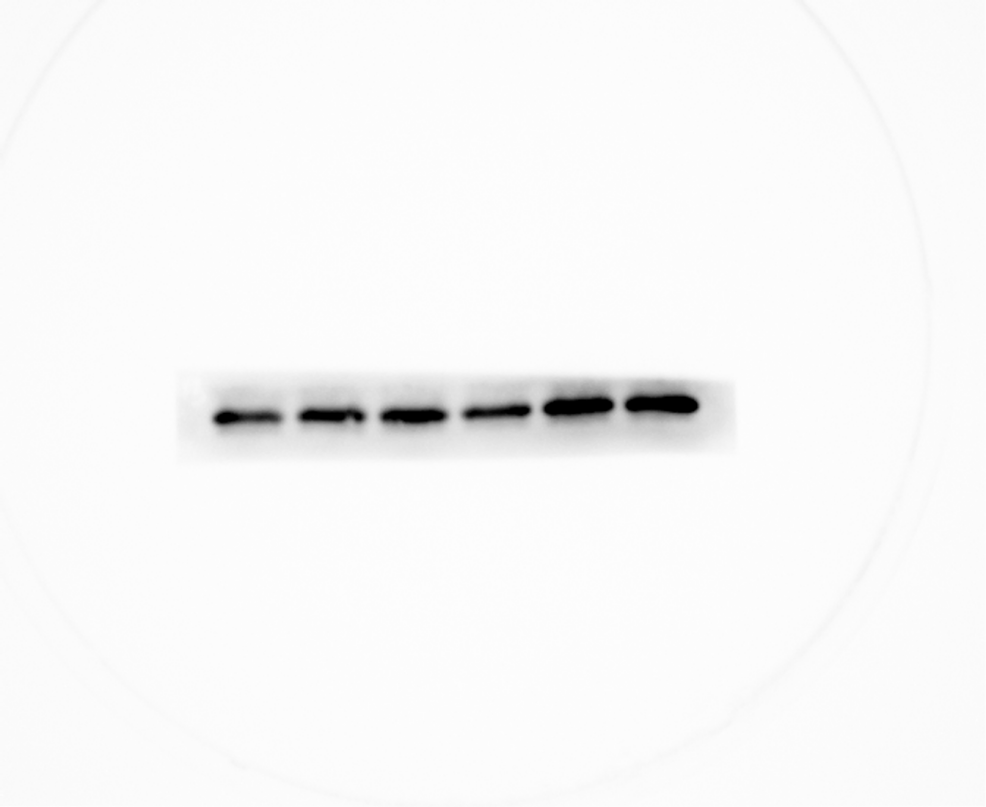
Caspase6**

**
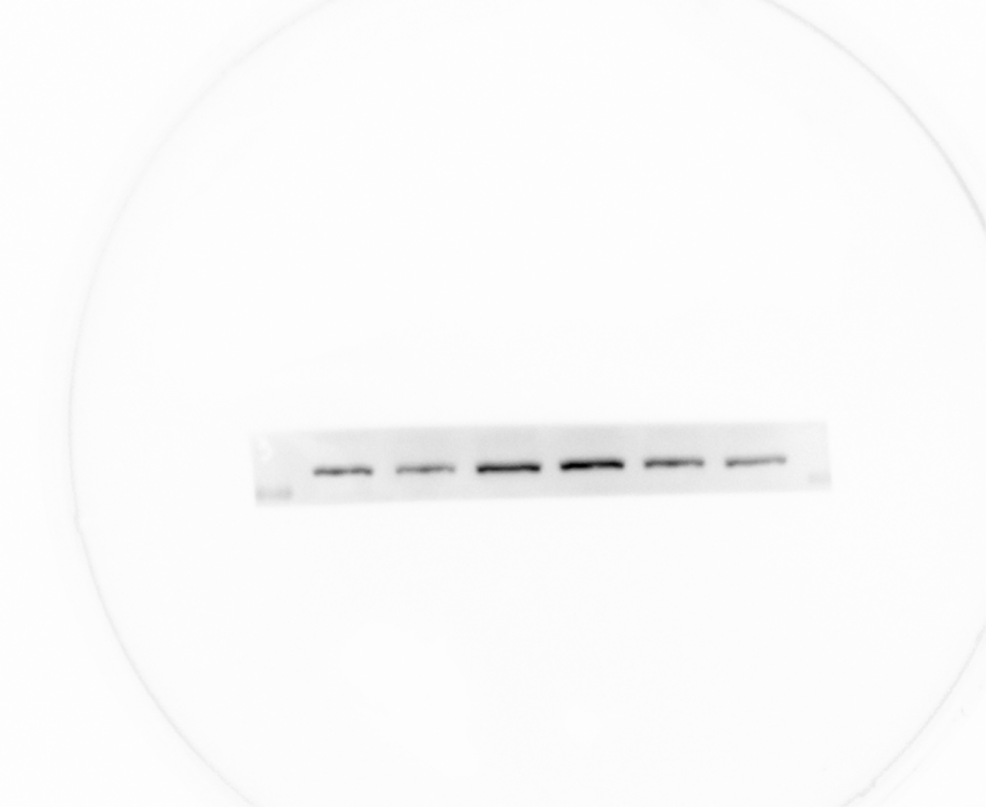
Cleaved Caspase9**

**
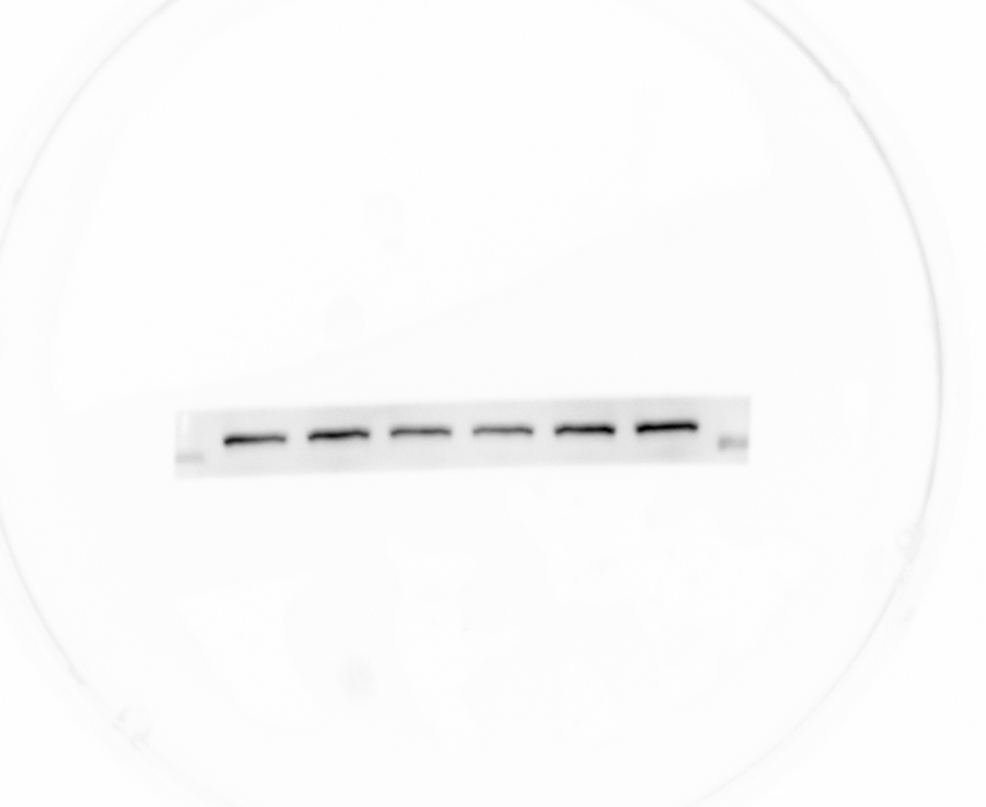
Caspase9**

**
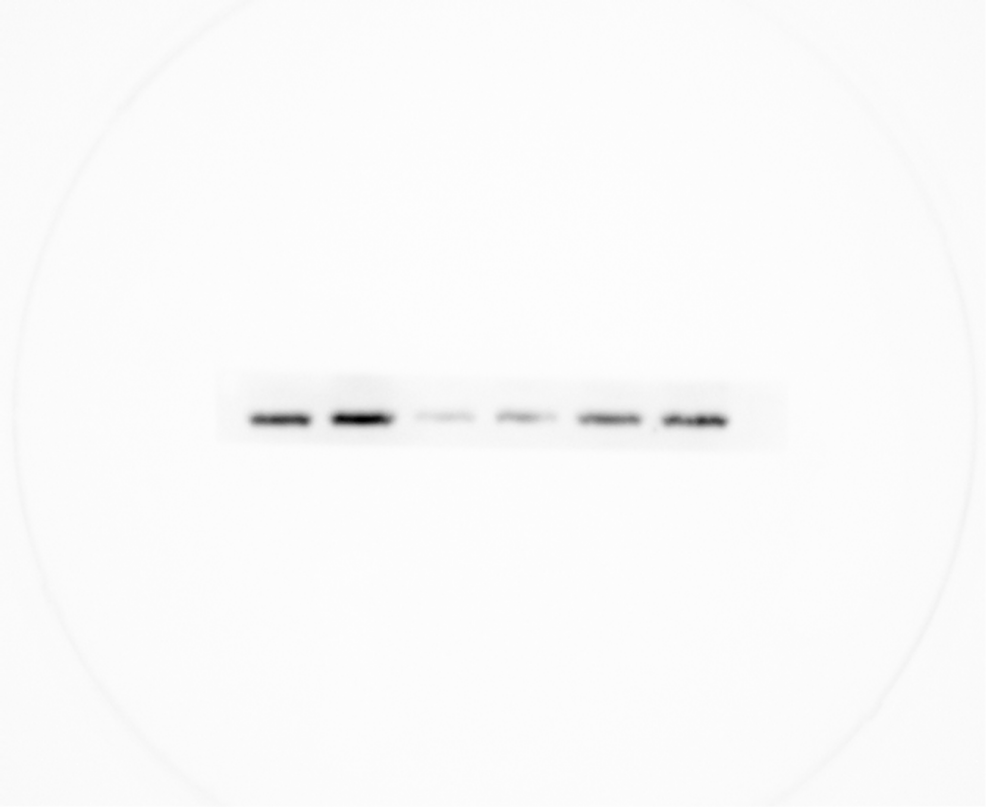
Bcl-2**

**
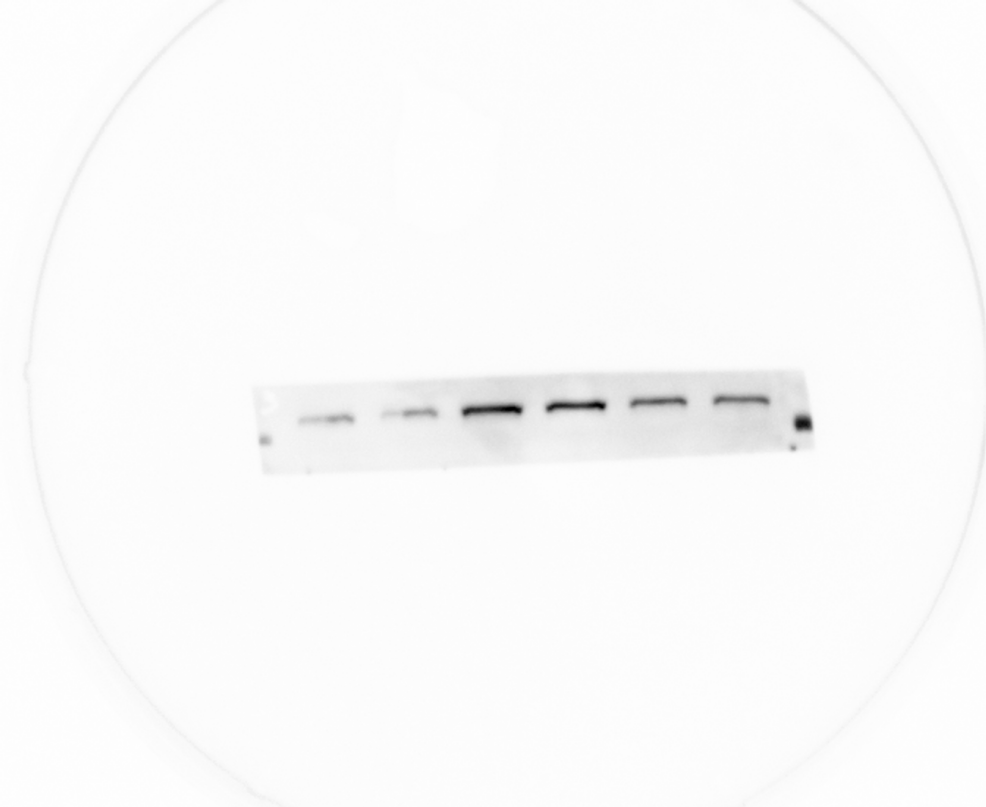
Bax**

**
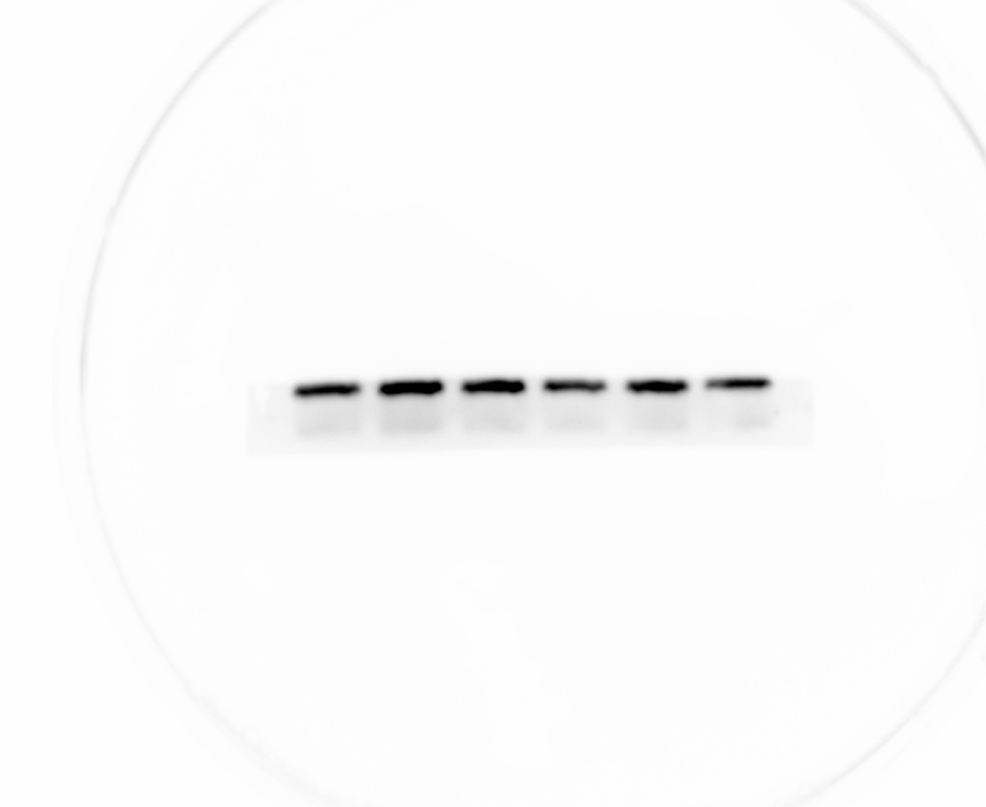
β-actin**

**Fig 5E liver tissues**

**
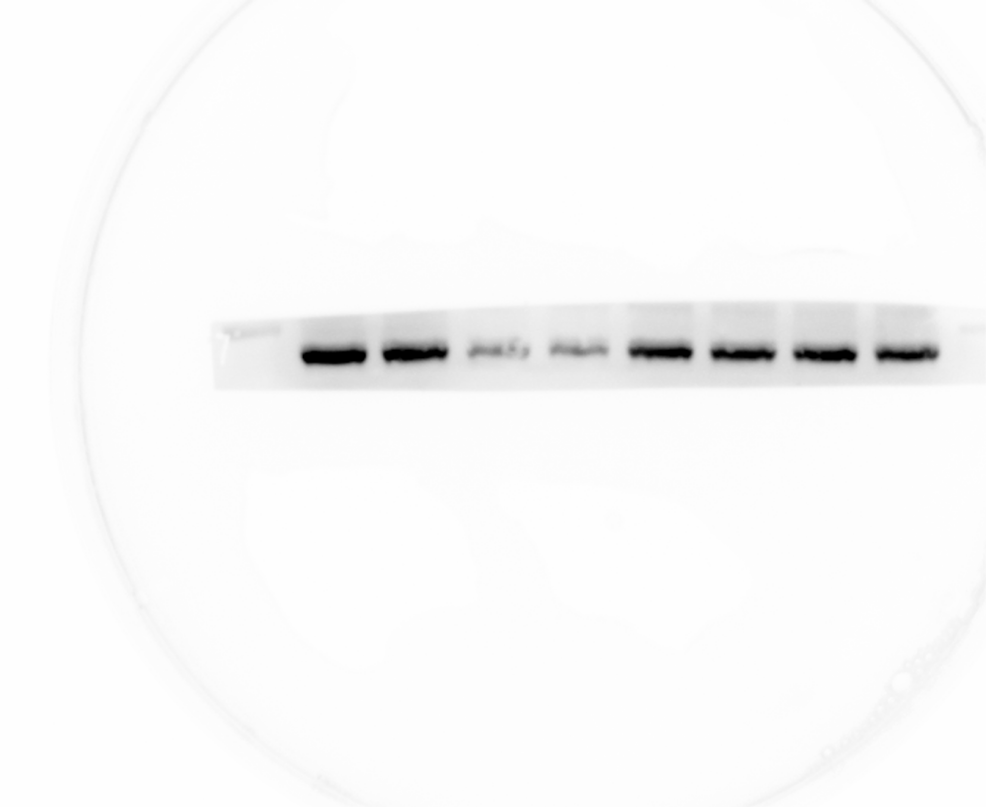
LXRα**

**
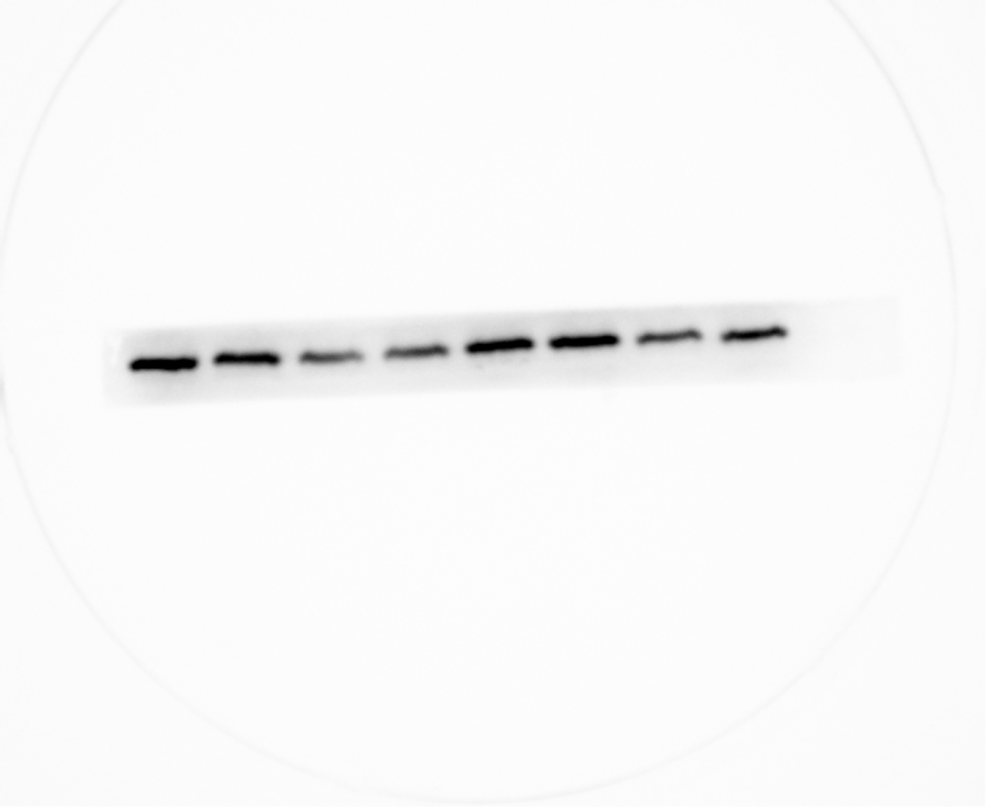
ABCA1**

**
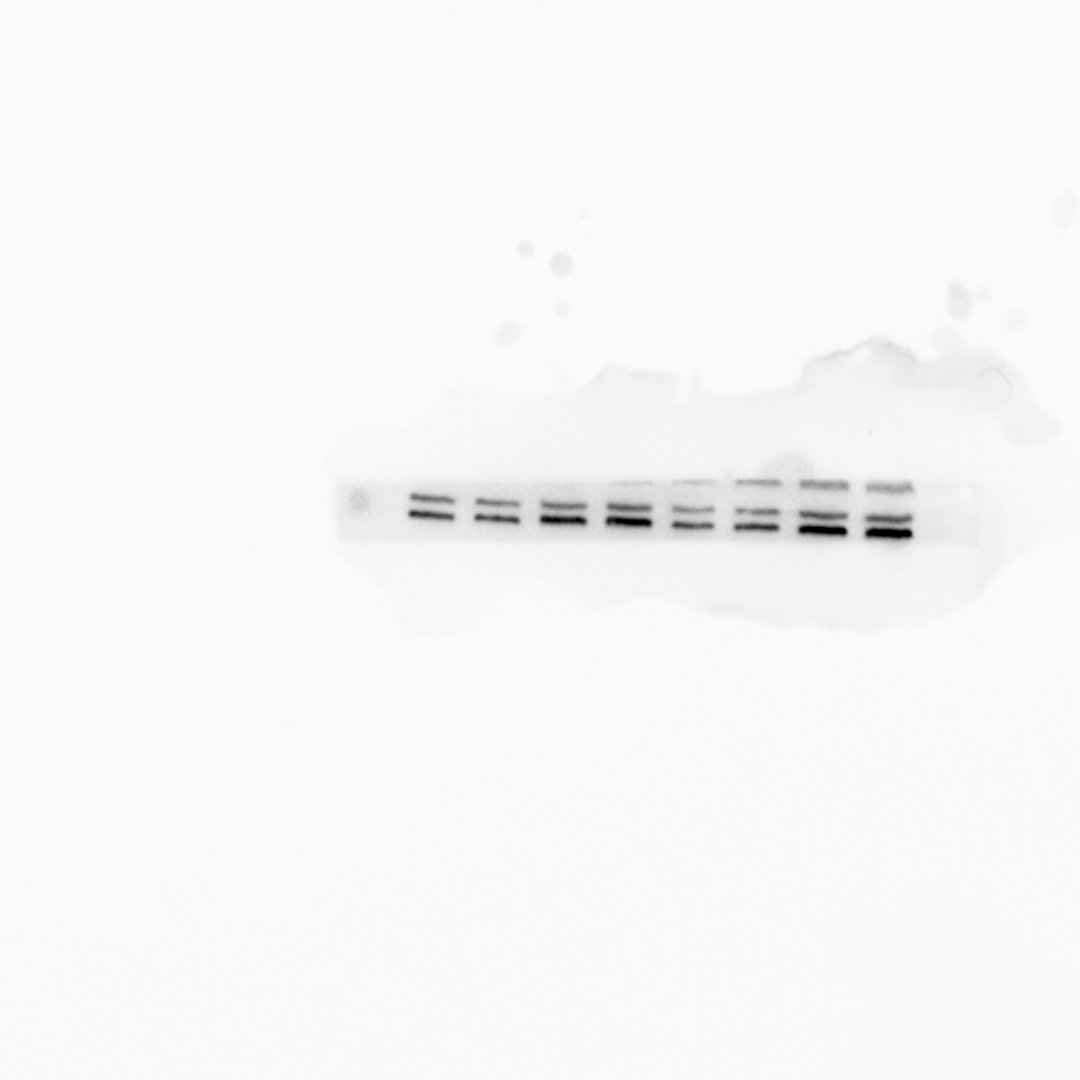
P-ERK1/2**

**

ERK1/2**

**

P-JNK**

**
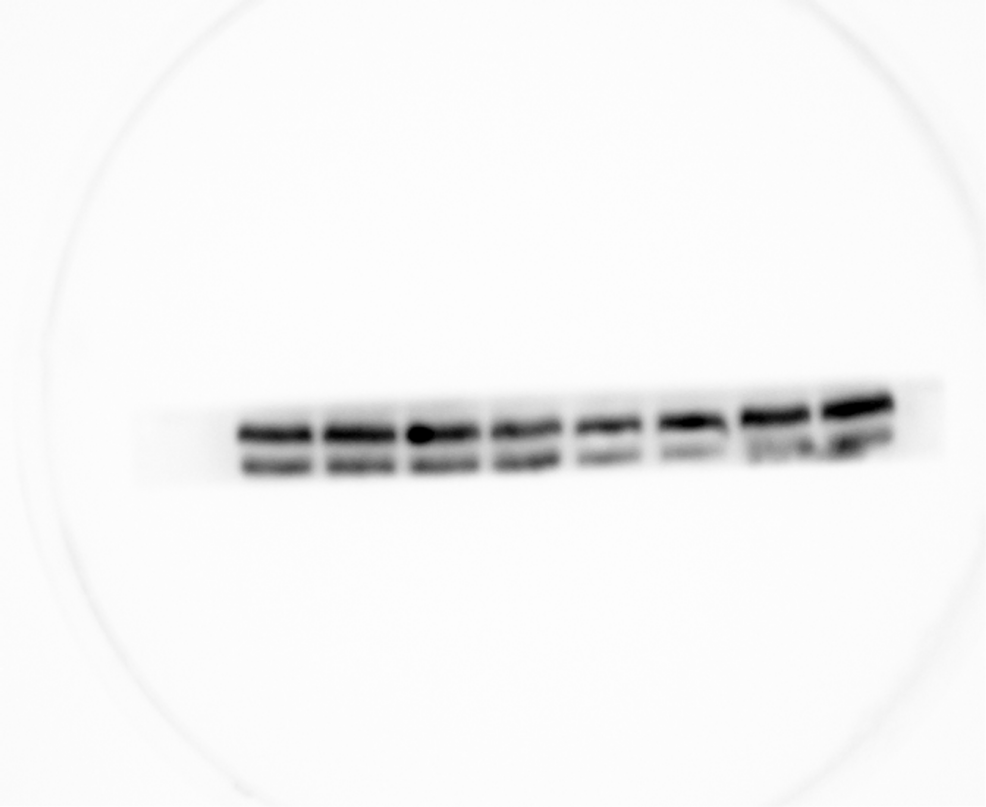
JNK**

**
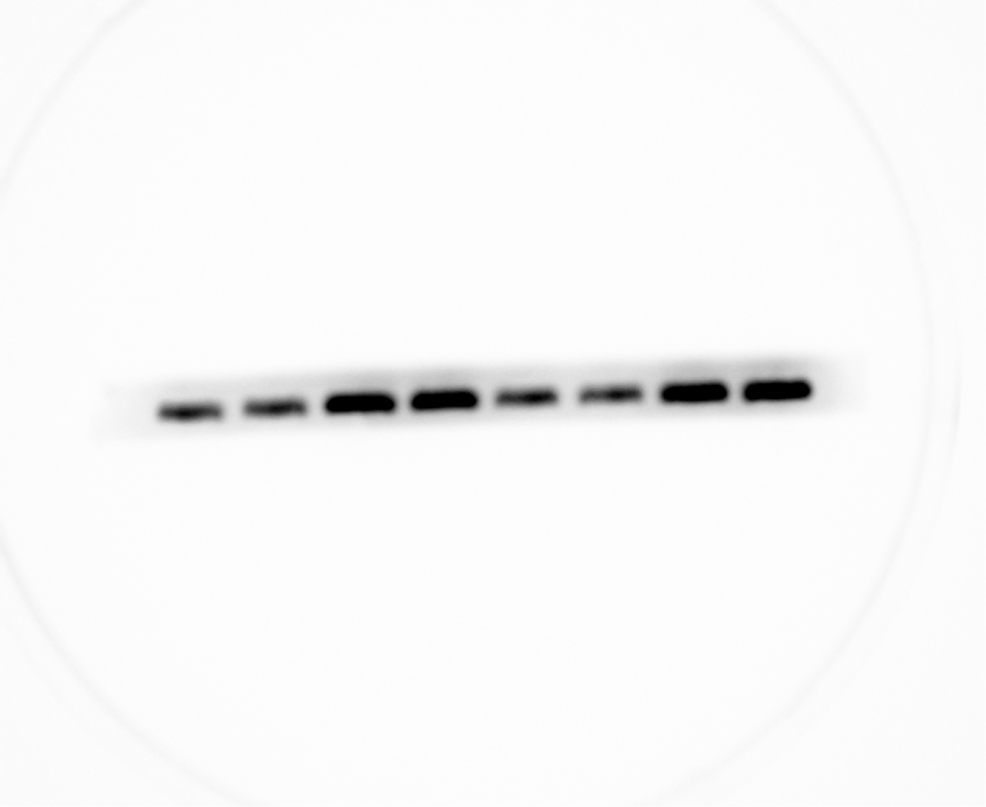
P-P38**

**
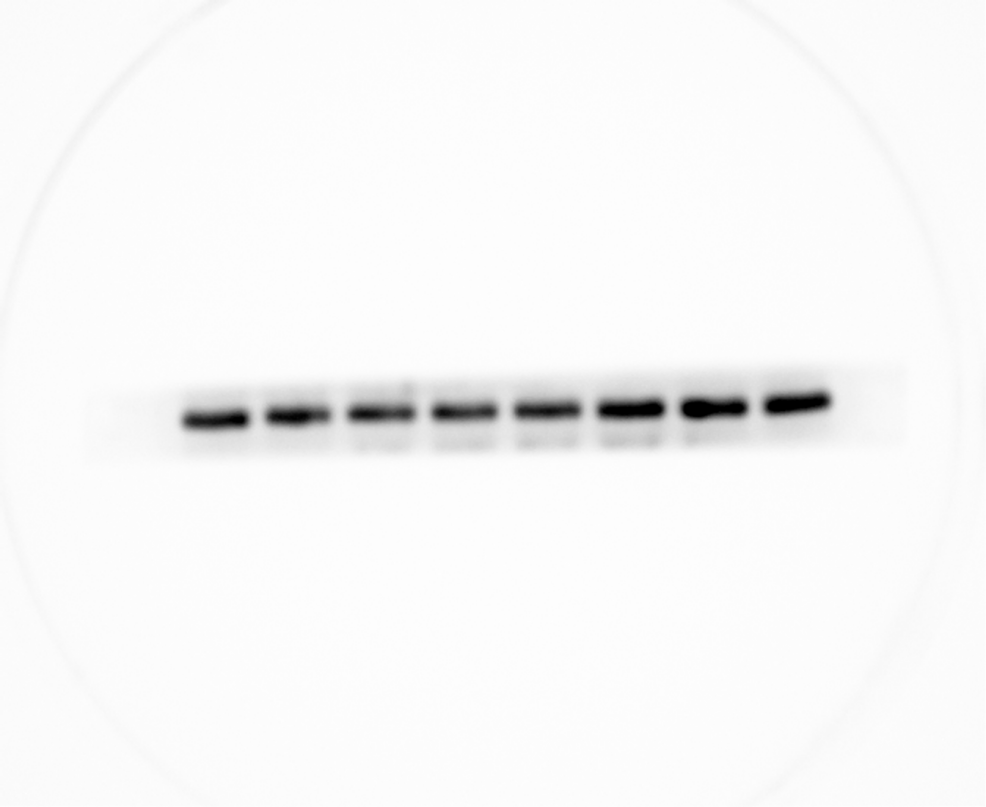
P38**

**
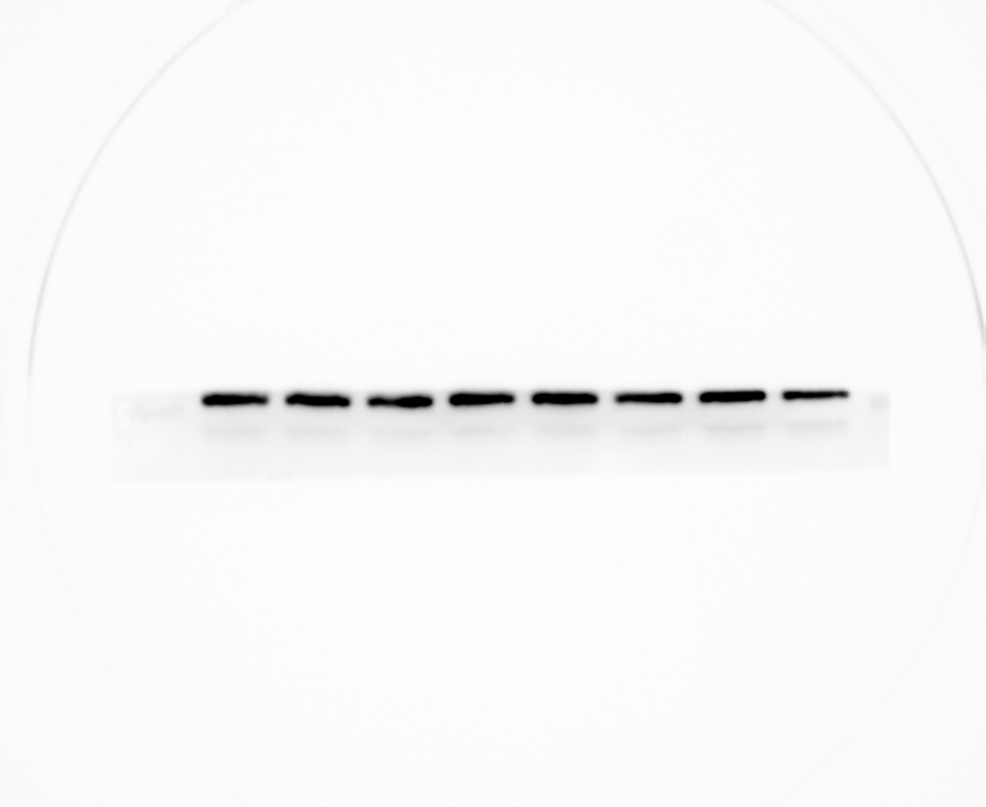
β-actin**

**
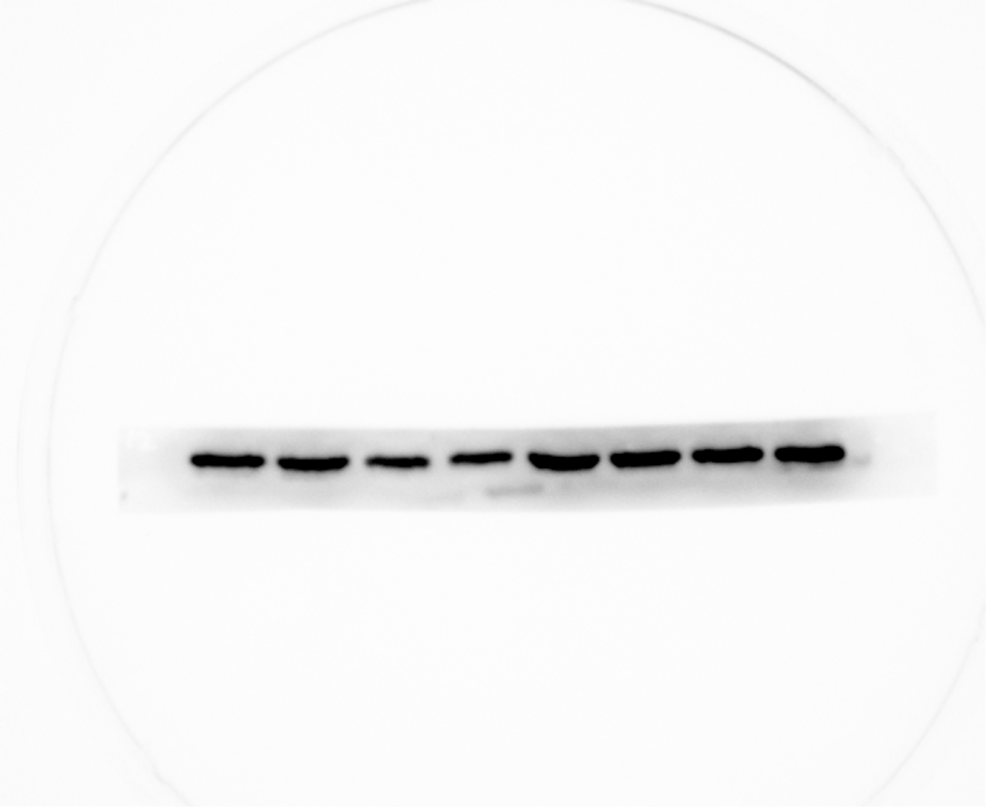
P-mTOR**

**
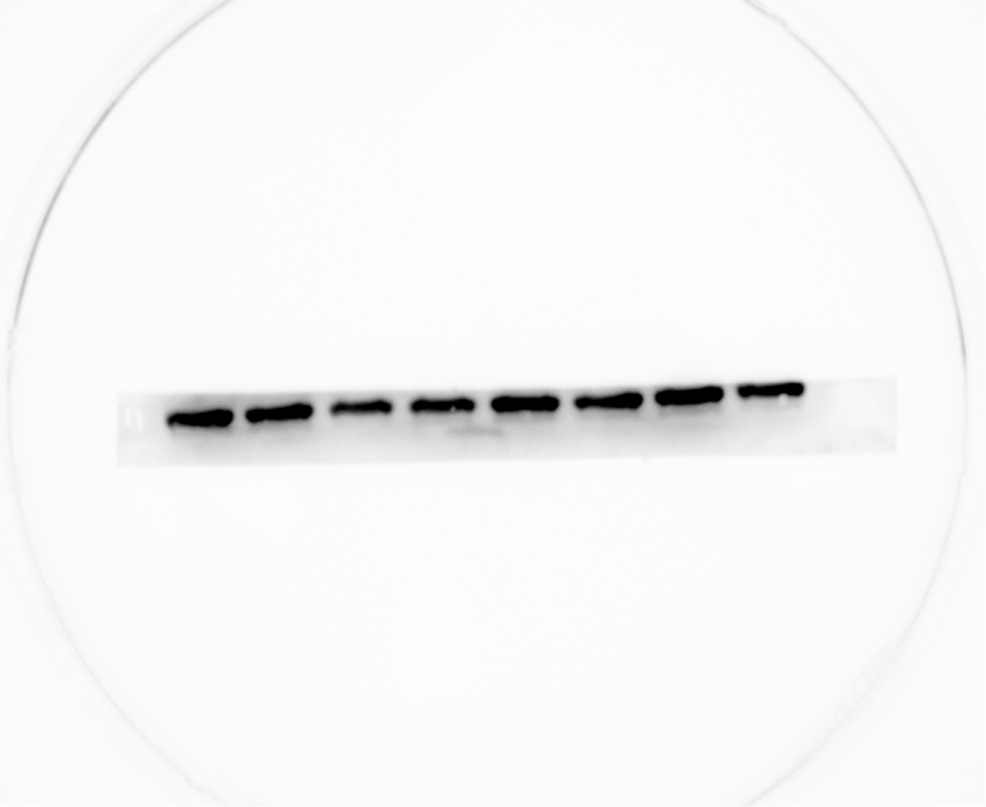
mTOR**

**
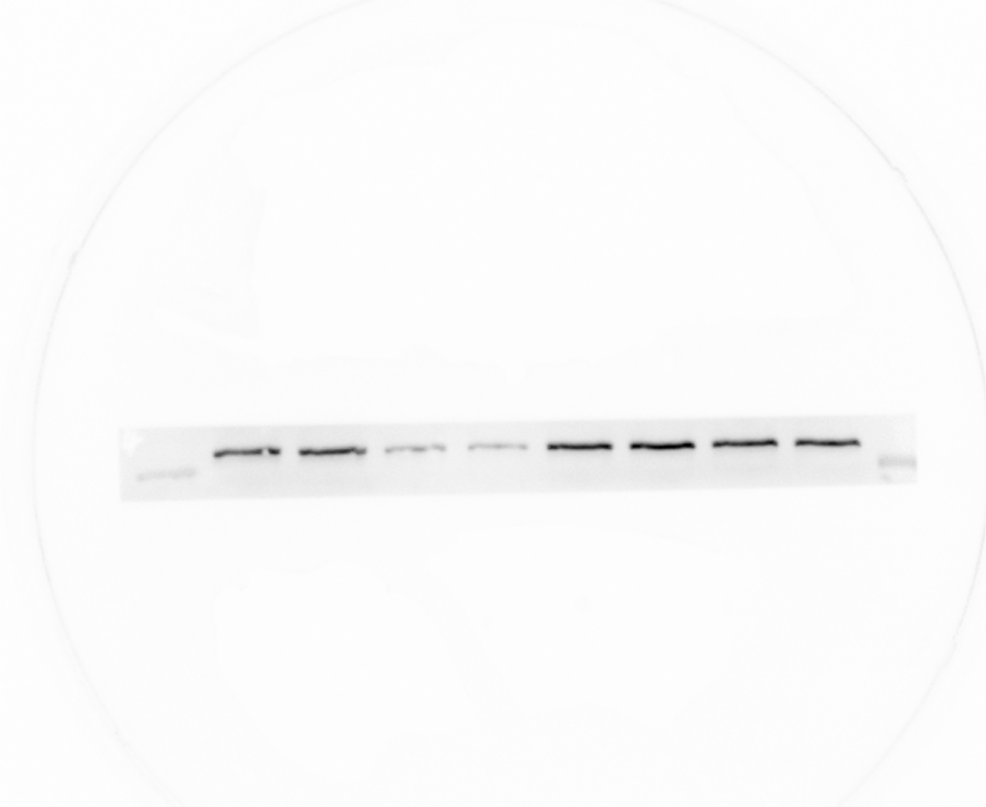
P-S6K**

**
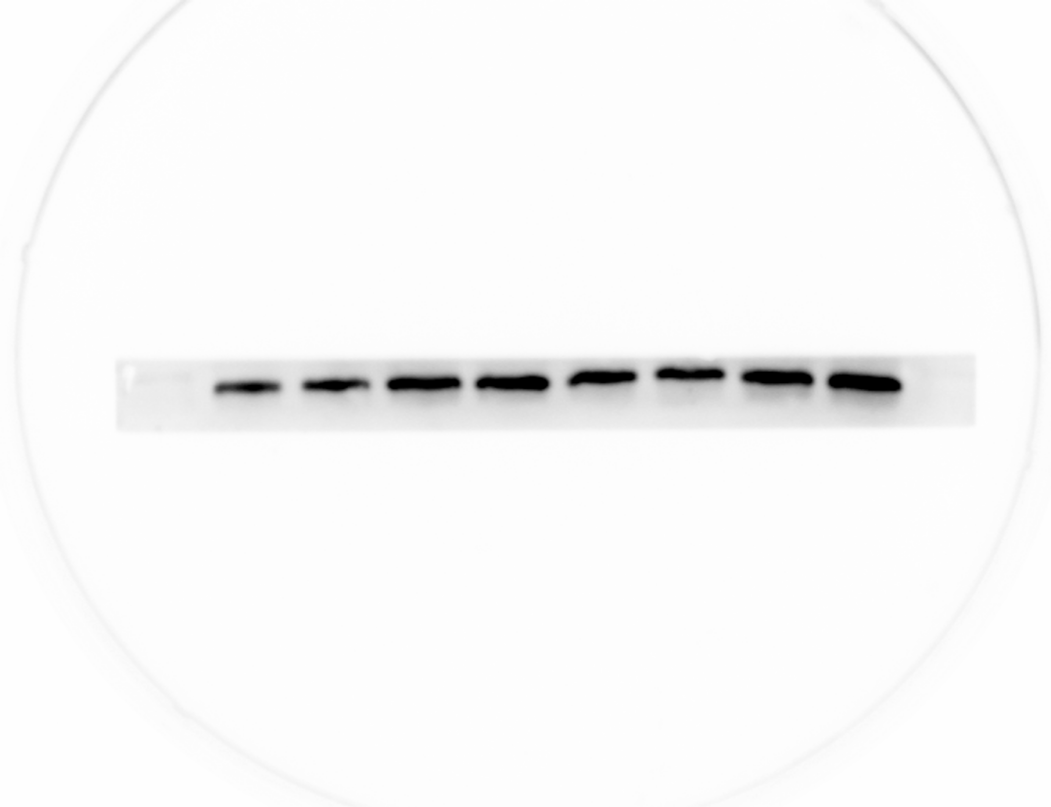
S6K**

**
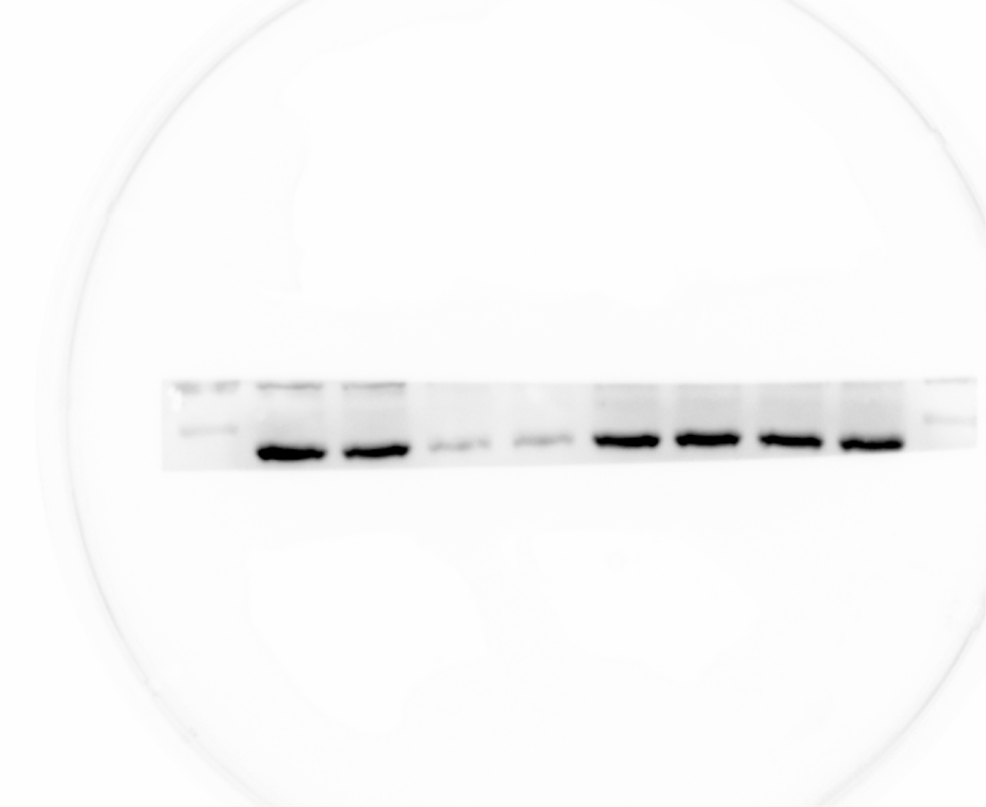
P-4E-BP**

**
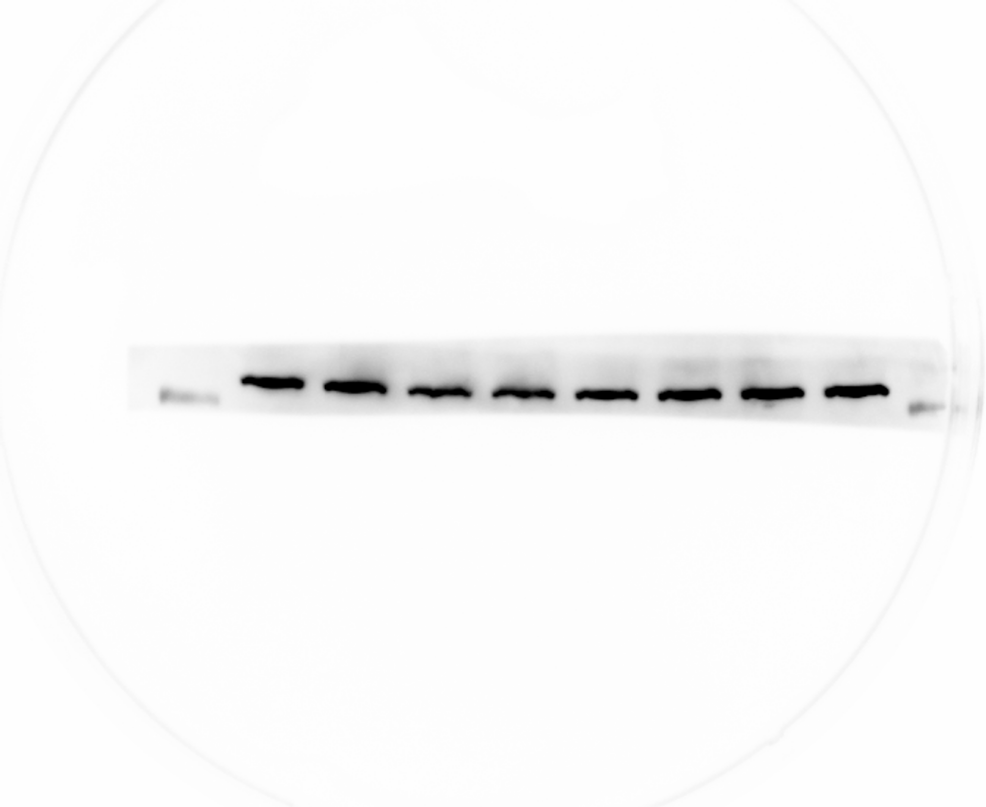
4E-BP**

**
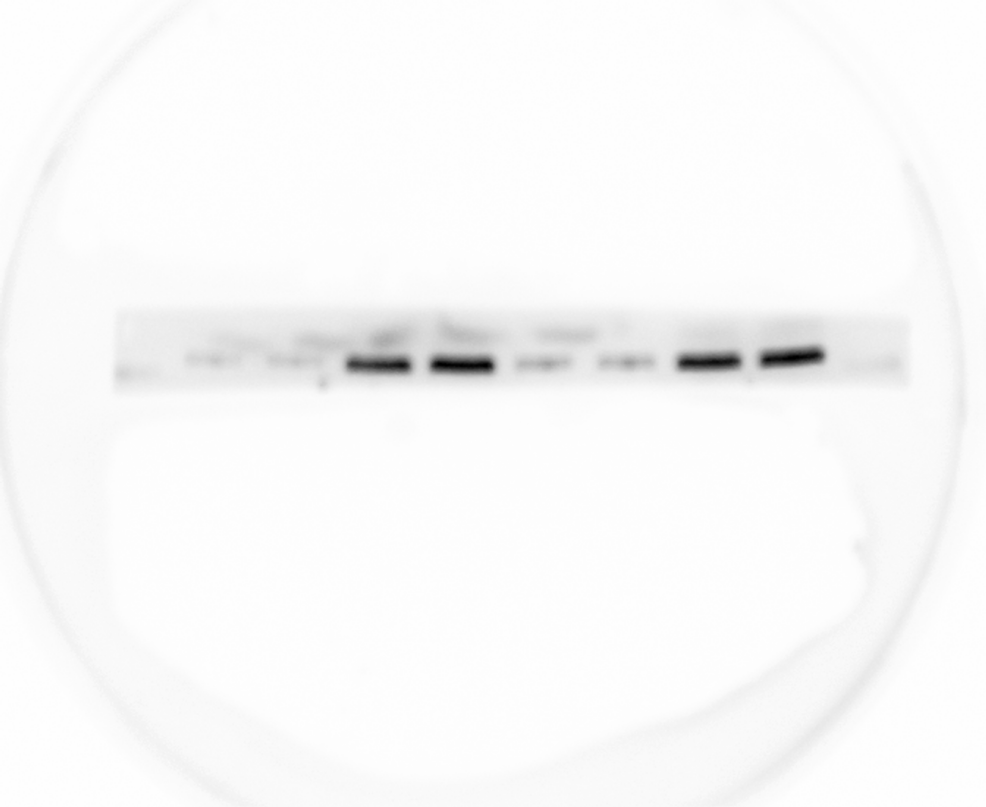
IL-6**

**
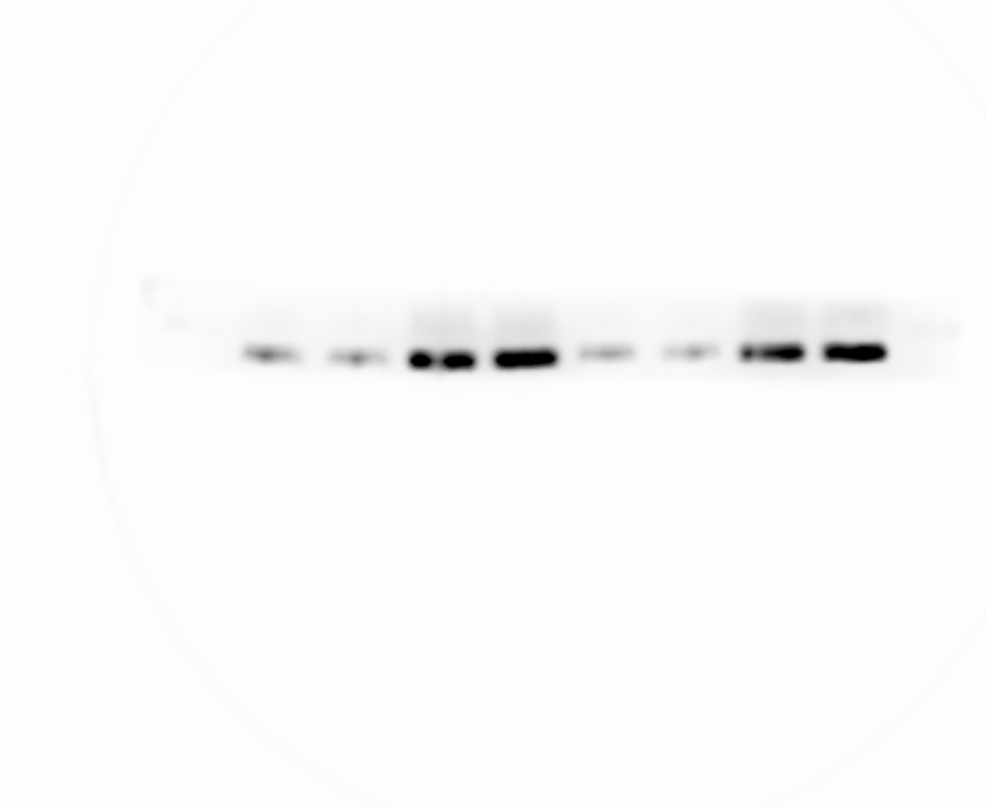
TNF-α**

**
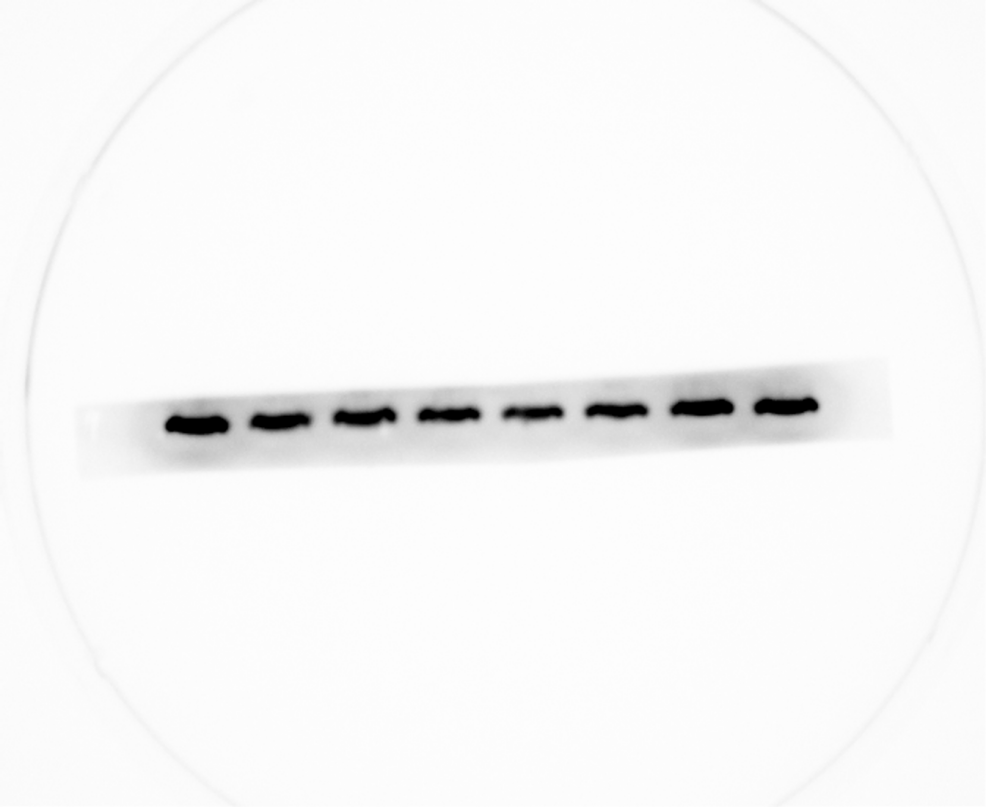
β-actin**

**Fig 6A liver tissues**

**
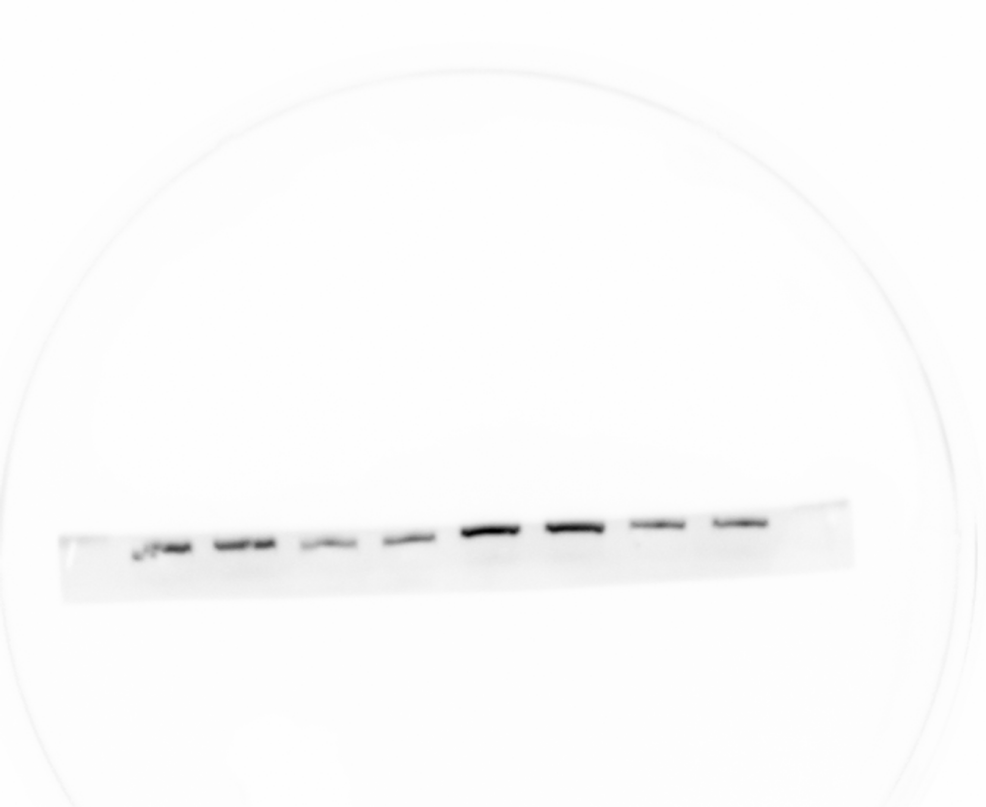
P-PI3K**

**
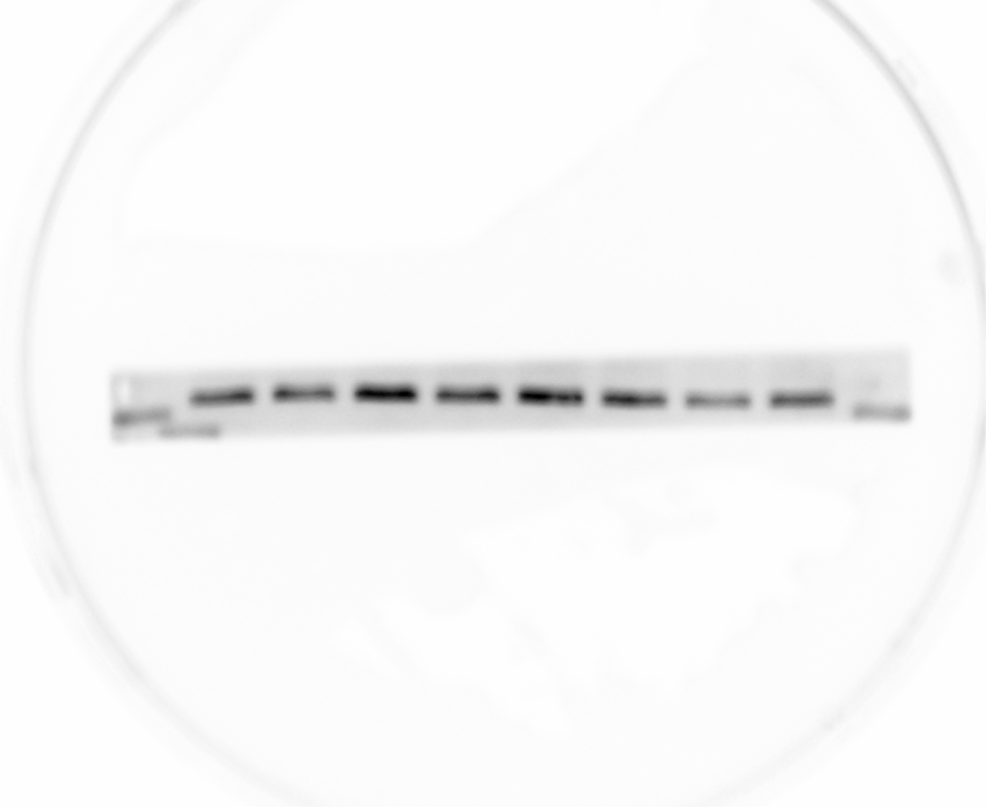
PI3K**

**
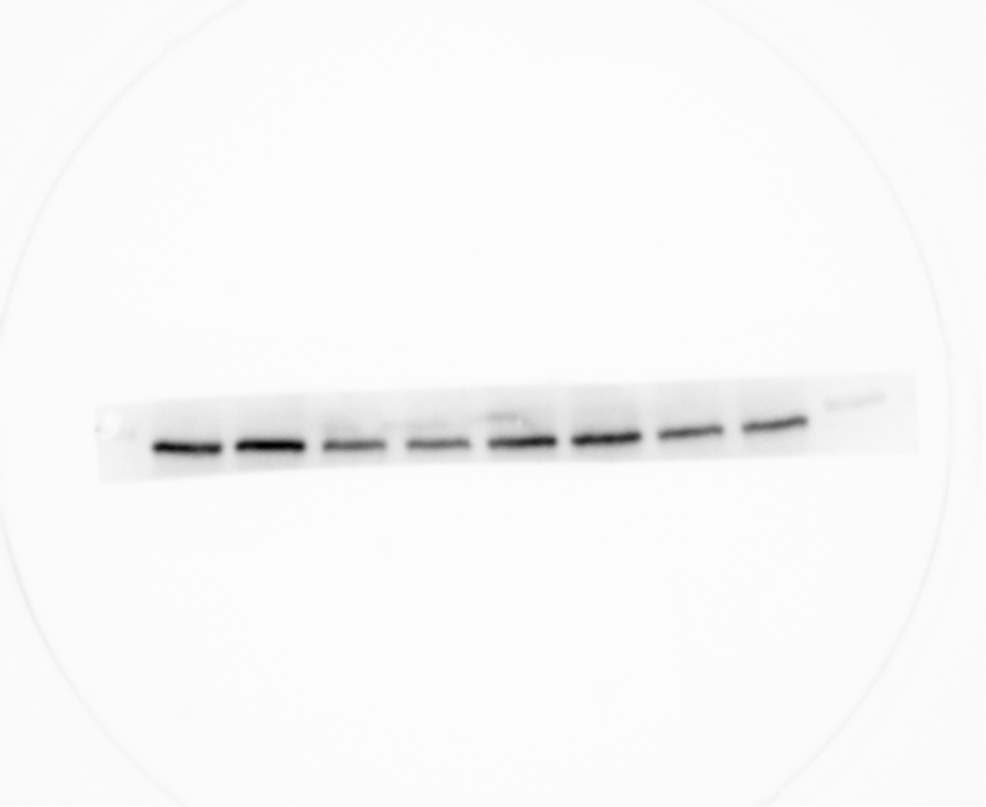
P-AKT**

**
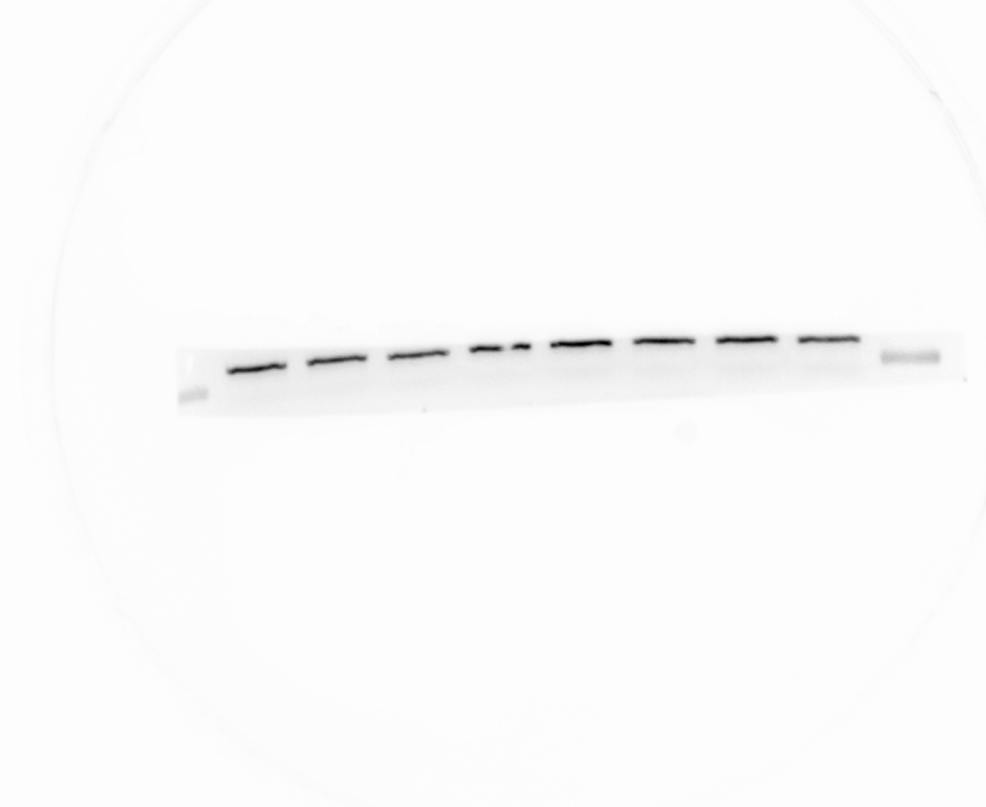
AKT**

**
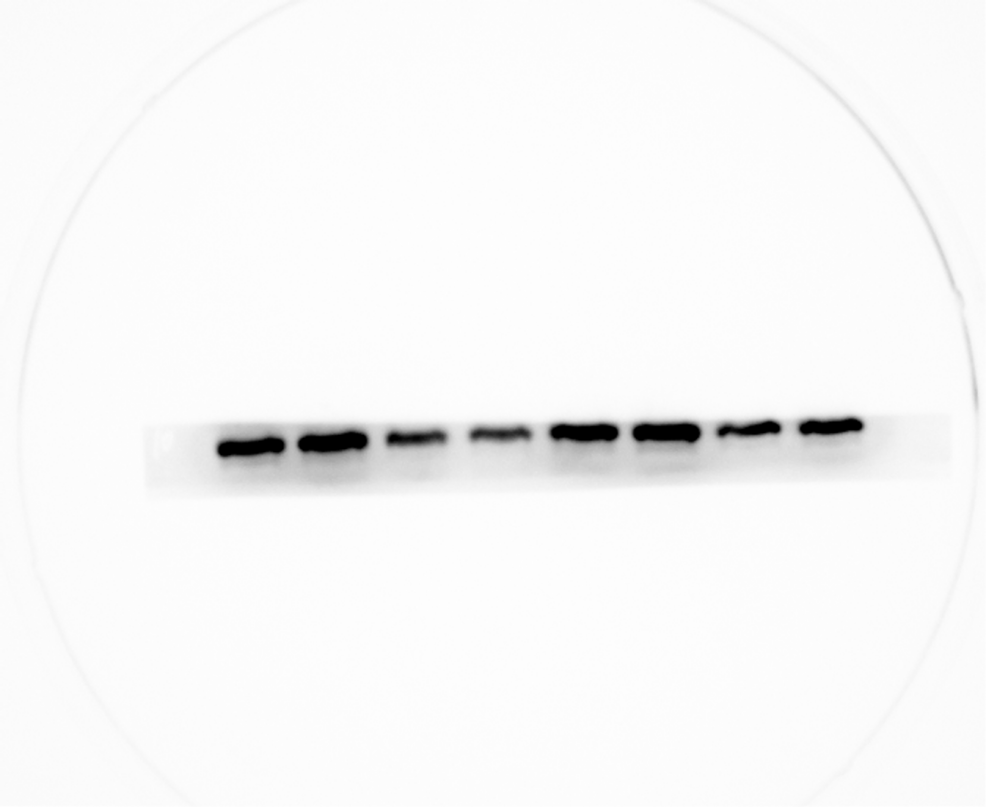
P-mTOR**

**
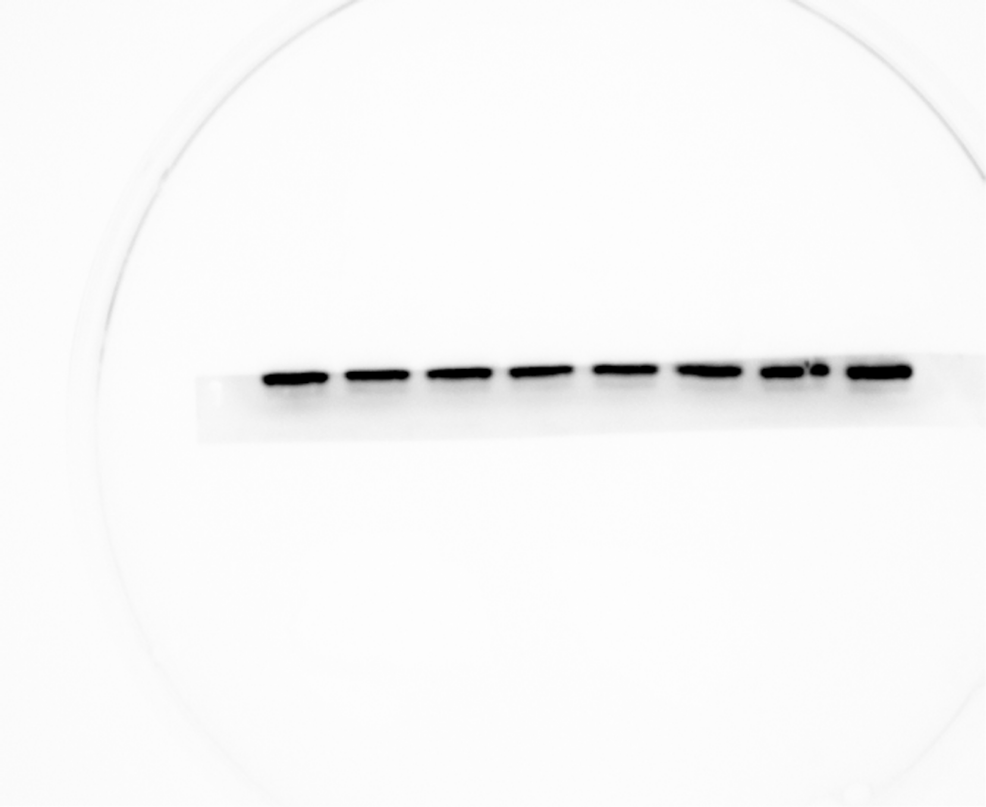
mTOR**

**

P-S6K**

**
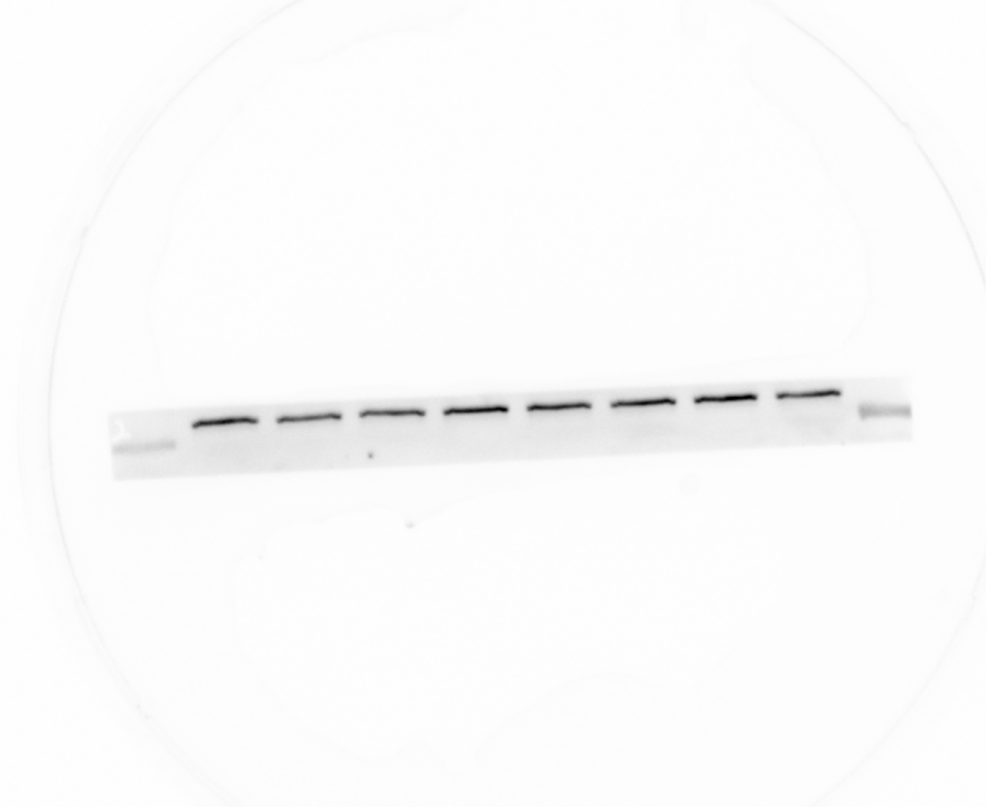
S6K**

**
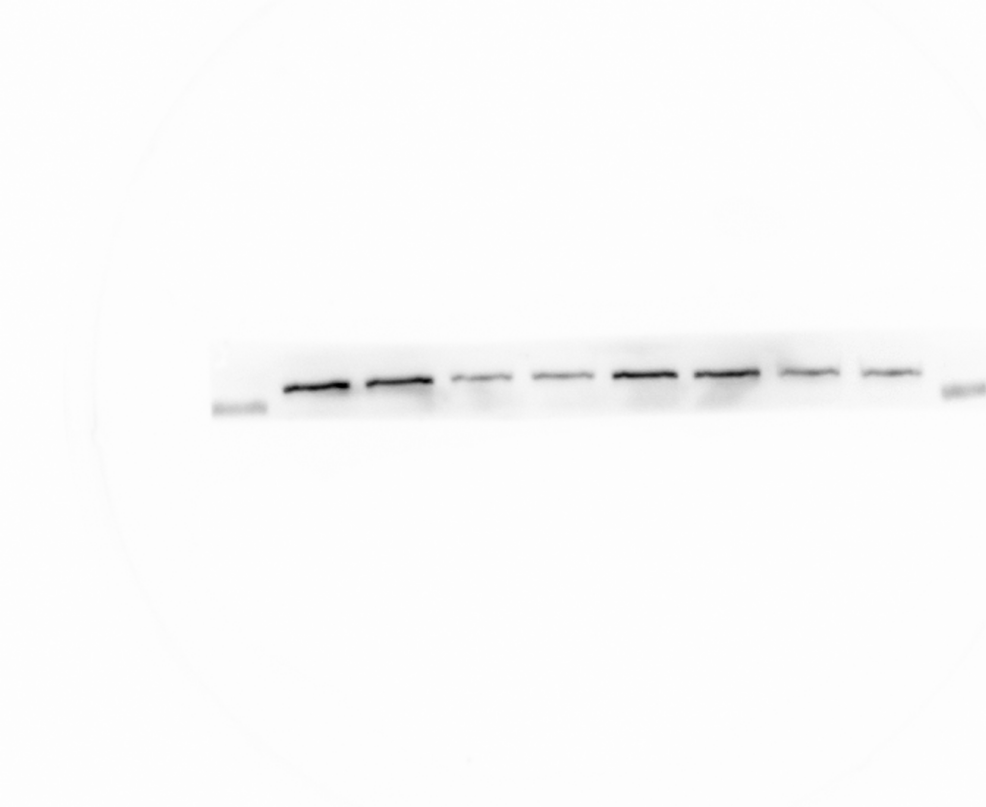
P-4E-BP**

**
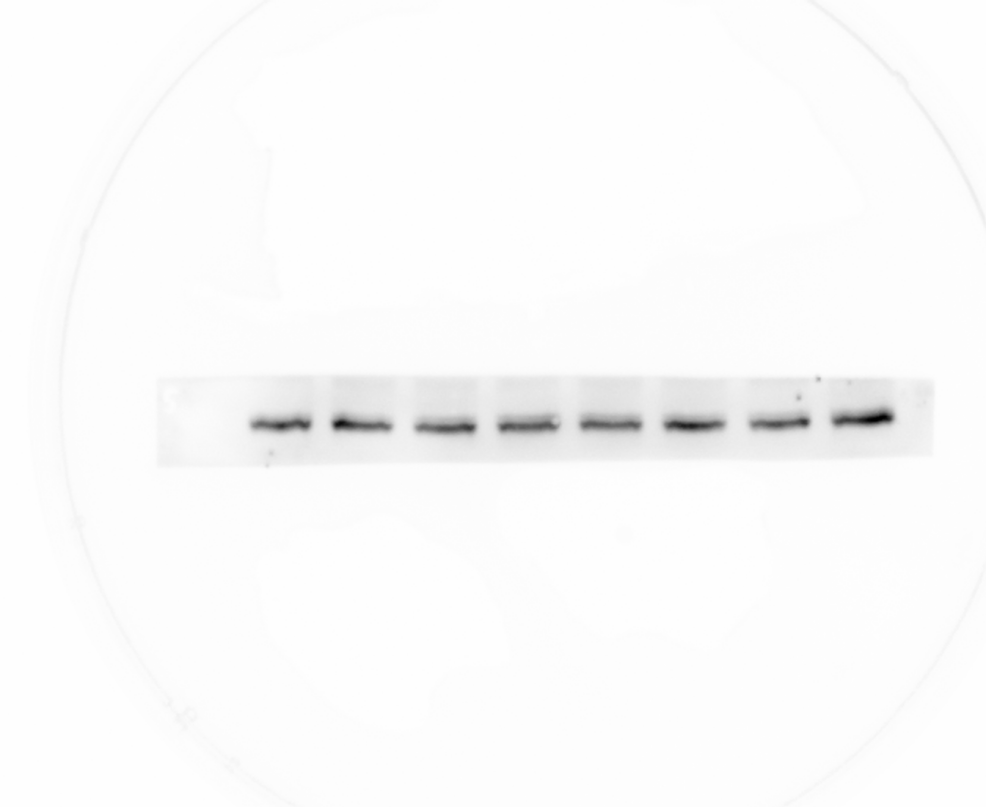
4E-BP**

**
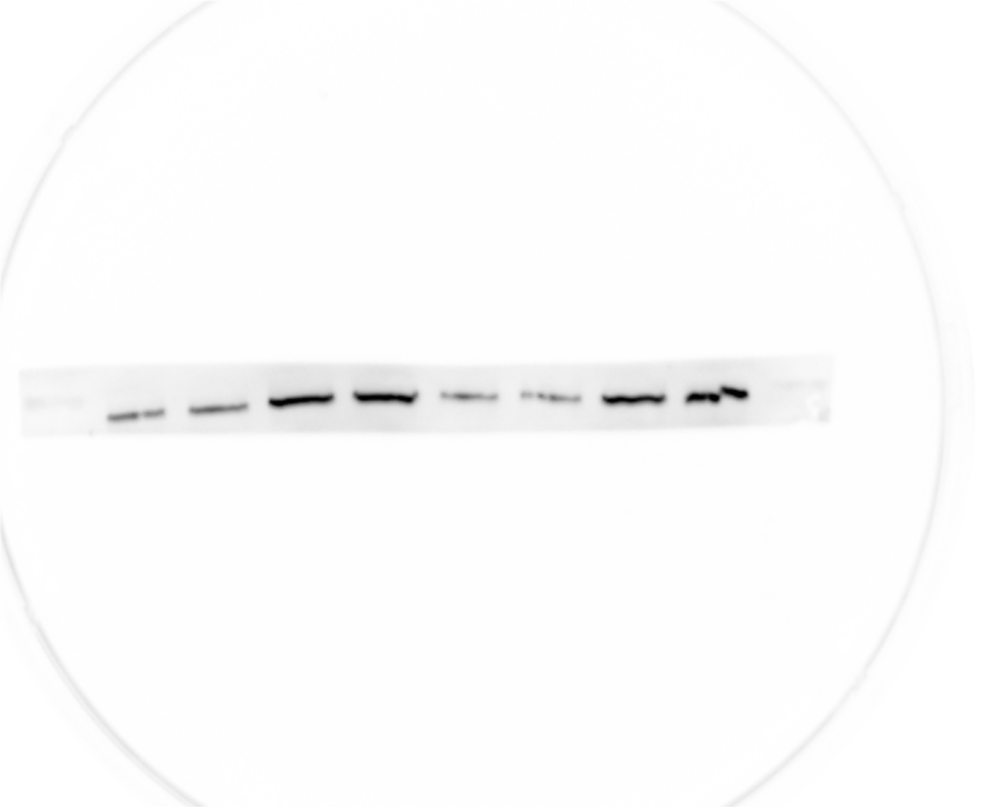
iNOS**

**
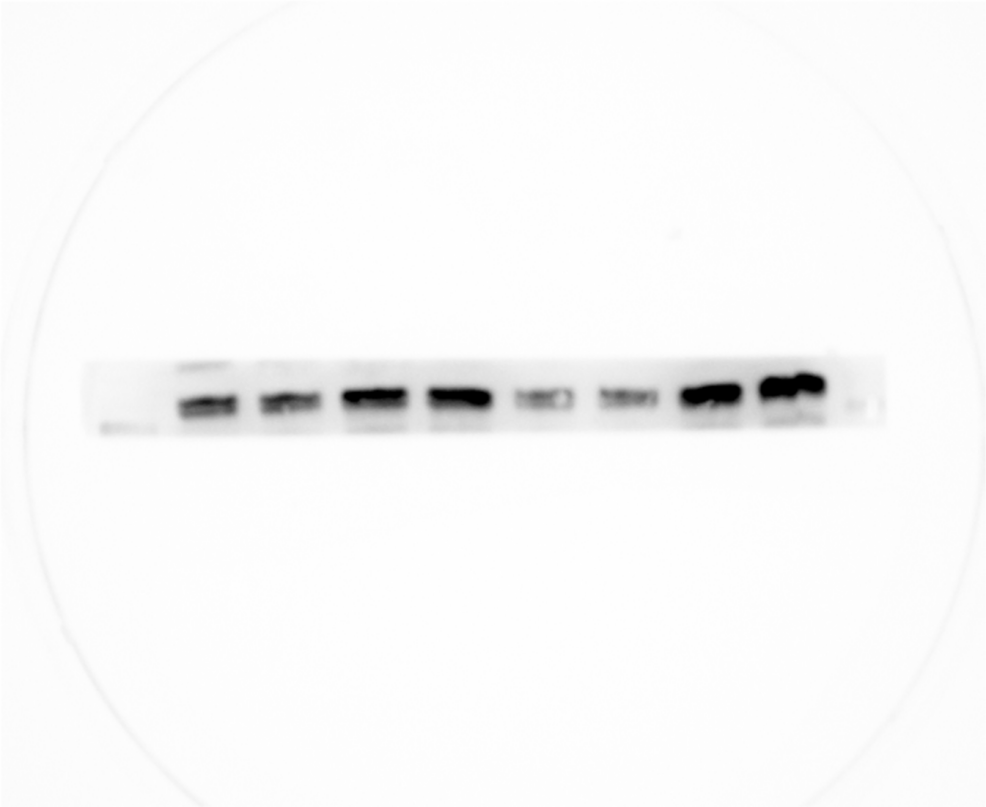
CD86**

**
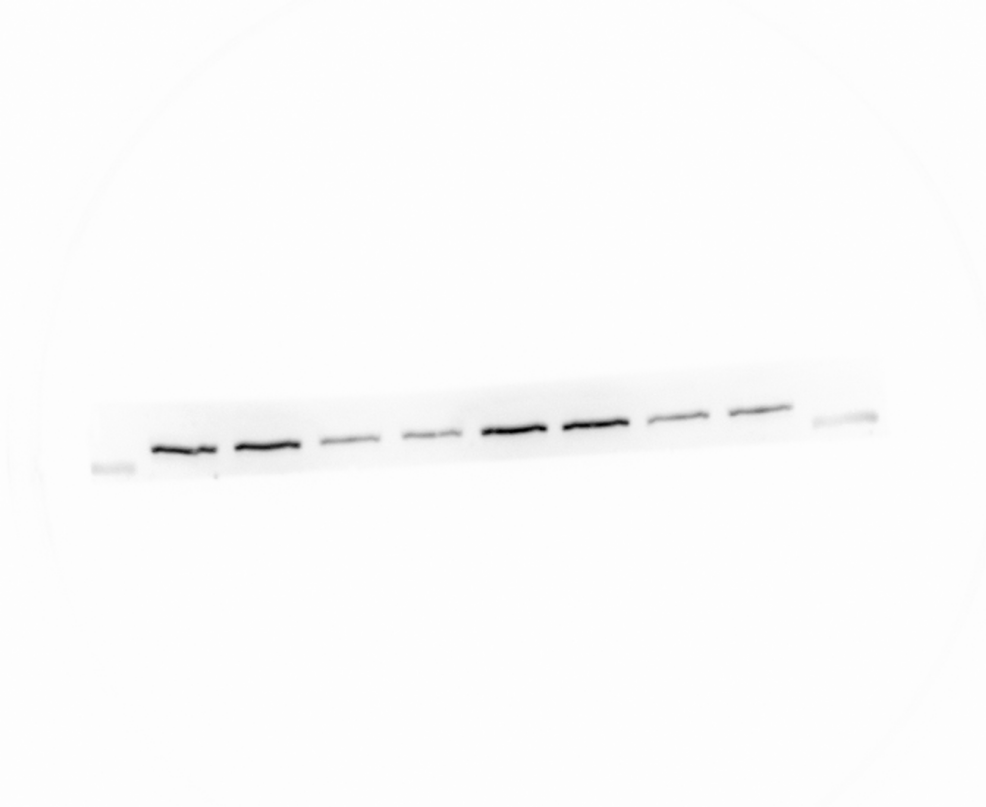
Arg-1**

**
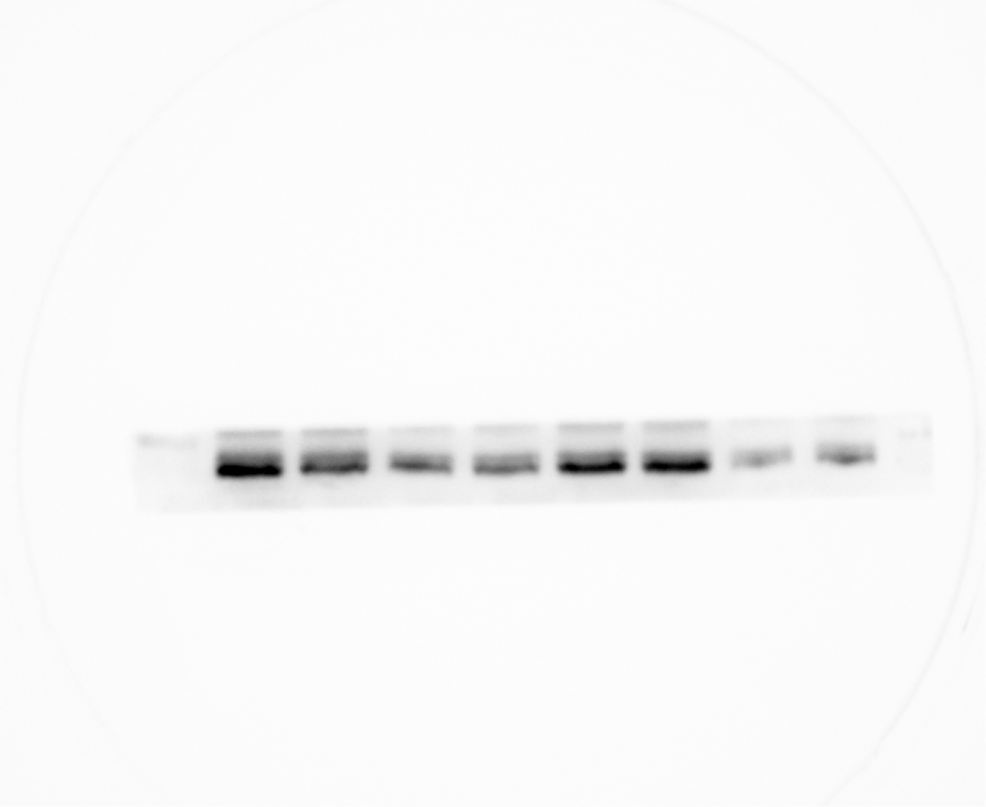
CD206**

**
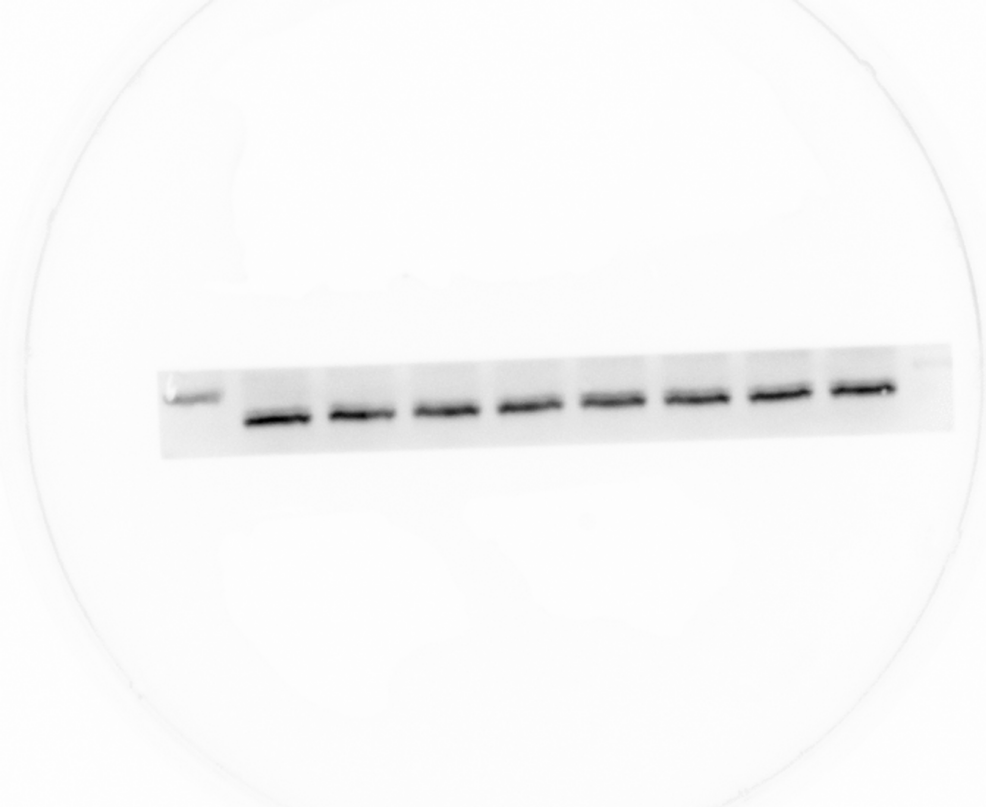
β-actin**
